# A Critical View of the Application of the APEX Software (Aqueous Photochemistry of Environmentally-Occurring Xenobiotics) to Predict Photoreaction Kinetics in Surface Freshwaters

**DOI:** 10.3390/molecules25010009

**Published:** 2019-12-18

**Authors:** Davide Vione

**Affiliations:** Dipartimento di Chimica, Università di Torino, Via Pietro Giuria 5, 10125 Torino, Italy; davide.vione@unito.it; Tel.: +39-011-670-5296

**Keywords:** pollutant fate, freshwater photochemistry, environmental modeling, aquatic environments, photoinduced transformation, sunlit surface waters, climate change

## Abstract

The APEX (aqueous photochemistry of environmentally occurring xenobiotics) software computes the phototransformation kinetics of compounds that occur in sunlit surface waters. It is free software based on Octave, and was originally released in 2014. Since then, APEX has proven to be a remarkably flexible platform, allowing for the addressing of several environmental problems. However, considering APEX as a stand-alone software is not conducive to exploiting its full potentialities. Rather, it is part of a whole ecosystem that encompasses both the software and the laboratory protocols that allow for the measurement of substrate photoreactivity parameters. Coherently with this viewpoint, the present paper shows both how to use APEX, and how to experimentally derive or approximately assess the needed input data. Attention is also given to some issues that might provide obstacles to users, including the extension of APEX beyond the simple systems for which it was initially conceived. In particular, we show how to use APEX to deal with compounds that undergo acid–base equilibria, and with the photochemistry of systems such as stratified lakes, lakes undergoing evaporation, and rivers. Hopefully, this work will provide a reference for the smooth use of one of the most powerful instruments for the modeling of photochemical processes in freshwater environments. All authors have read and agreed to the published version of the manuscript.

## 1. Introduction

Pollutants occurring in natural waters pose a serious threat to aquatic ecosystems and human health, via both recreational activities and the use of irrigation or drinking water. The problems connected with water pollution by organic chemicals would be much more serious than currently experienced, were natural aquatic systems unable to self-depollute. Among the natural self-depollution processes are hydrolysis, redox reactions, and, most notably, bio- and photodegradation [1]. Photodegradation is particularly effective in the case of biorecalcitrant pollutants, and many contaminants of emerging concern emitted by wastewater treatment plants (WWTPs) belong to this class. Indeed, WWTPs tend to select for hardly biodegradable and water-soluble pollutants that are most likely to survive the activated sludge step, without being biodegraded or adsorbed on biosolids [2].

Photoreactions in sunlit surface waters can be divided into direct photolysis and indirect photochemistry. In the former case, sunlight is absorbed by the pollutant itself and the absorption process triggers transformation (e.g., ionization, bond breaking, or reaction with the solvent or with other dissolved molecules). Indirect photoreactions involve the absorption of sunlight by photosensitizers, the main ones in surface waters being chromophoric dissolved organic matter (CDOM), nitrate, and nitrite [3,4]. The irradiation of all the mentioned photosensitizers yields the photoreactive transient ^•^OH (hydroxyl radical), which can produce another transient (CO_3_^•−^, the carbonate radical) upon reaction with inorganic carbon species (HCO_3_^−^ and CO_3_^2−^) [5]. Irradiated CDOM can also produce reactive triplet states (^3^CDOM*), and the latter yield singlet oxygen (^1^O_2_) upon reaction with dissolved O_2_ [3,6]. All the mentioned transient species (^•^OH, CO_3_^•−^, ^3^CDOM*, and ^1^O_2_) are involved to some extent in indirect pollutant phototransformation, depending on the reactivity of the given pollutant towards each transient [7,8]. Although they play a key role in indirect phototransformation of pollutants, the transients are mostly scavenged/quenched by natural water components. The latter include: (i) dissolved organic matter (DOM, either chromophoric or not), HCO_3_^−^ and CO_3_^2−^ in the case of ^•^OH (plus Br^−^, which plays the main role in some saltwaters and in seawater); (ii) DOM again in the case of CO_3_^•−^; (iii) dissolved O_2_ for ^3^CDOM*; and (iv) the water solvent for ^1^O_2_ [7]. The main mentioned formation and scavenging processes are summarized by the reactions below [3,4,5,6,7]:

NO_3_^−^ + hν + H^+^ → ^•^NO_2_ + ^•^OH (1)

NO_2_^−^ + hν + H^+^ → ^•^NO + ^•^OH (2)

CDOM + hν →→→ ^•^OH (3)

CDOM + hν → ^1^CDOM* → ^3^CDOM* (4)

^3^CDOM* + O_2_ → CDOM + ^1^O_2_ (5)

^•^OH + HCO_3_^−^ → H_2_O + CO_3_^•−^ (6)

^•^OH + CO_3_^2−^ → OH^−^ + CO_3_^•−^ (7)

^3^CDOM* + CO_3_^2−^ → CDOM^•−^ + CO_3_^•−^ (8)

^•^OH + DOM → Products (9)

CO_3_^•−^ + DOM → Products (10)

^1^O_2_ + H_2_O → O_2_ + H_2_O (11)

The indirect phototransformation of pollutants is usually a minor quenching pathway for the transient species; thus, their steady-state levels ([^•^OH], [CO_3_^•−^], [^3^CDOM*], and [^1^O_2_]) typically do not depend on the pollutant concentration. This issue means that it is possible to study or model the photochemical functioning of natural waters, independently of the occurrence of pollutant photoreactions [9].

To date, most attention has been focused on the photochemical properties of the single molecules/pollutants by dividing them into “photostable” and “photolabile” ones [10]. In contrast, very little interest has been devoted to the photochemical characterization of natural ecosystems, despite the fact that their photoreactivity can also widely vary. In fact, a photolabile molecule in a poorly photoreactive environment can have the same phototransformation kinetics as a photostable molecule in a photoreactive water body [11]. The lack of attention paid towards the photochemical functioning of the natural aquatic systems is probably due to the difficulty of extrapolating results from laboratory experiments to real outdoor conditions, which has led to an important underestimation of the environmental variability.

The use of photochemical models is a good way to bridge the gap between laboratory results and outdoor conditions [12,13,14,15,16]. Some years ago, a useful tool was released for this purpose, namely the “aqueous photochemistry of environmentally occurring xenobiotics” (APEX) software [17]. The main goal of APEX is to predict the environmental phototransformation of pollutants in environmental waters based on their photoreaction kinetics parameters (absorption spectra, direct photolysis quantum yields, second-order reaction rate constants with transient species). In several cases, it has been possible to validate this approach by comparing the predicted kinetics with known field data of pollutant phototransformation. Validation has been obtained in the cases of carbamazepine [18], ibuprofen [19], diclofenac and naproxen [20], atrazine [21], and 2-methyl-4-chlorophenoxyacetic acid (MCPA) [22], as well as pesticide phototransformation intermediates such as 2,4-dichloro-6-nitrophenol [23], 2-nitro-4-chlorophenol [24], and 3,4-dichloroaniline [25].

After several years of using APEX, a number of clear advantages and disadvantages have come to light. The main advantage is probably the software’s flexibility, which has not only allowed for the modeling of pollutant phototransformation, but has also provided important insights into the photochemical functioning of natural surface waters [26]. Moreover, it has been possible to extend the model applications from the originally foreseen simple systems (stationary and well mixed ones, such as overturning lakes) to more complex environments such as stratified lakes, rivers, and to include additional phenomena such as evaporative water concentration [27]. This remarkable flexibility comes at a price, however, because ease of use has never been the strong point of APEX.

Despite this drawback, research groups located at the Institut de Chimie de Clermont-Ferrand (France) [16] and the Säo Paulo State University (Brasil) [28,29,30] have used APEX with very remarkable results, but the software diffusion took place to a lesser extent than initially hoped for. There have also been some indications that, at the moment, APEX may be more appreciated for its potential implications than actually used [31,32], thereby suggesting that user-friendliness may be an issue. Moreover, APEX strongly relies on the input data and, while plentiful information has been provided on how to use the software, little attention has been paid to how to experimentally obtain such data. The required procedures have been developed as well, but they have been outlined in sparse publications [20,25,33] and this has probably discouraged potential users.

While waiting for a (hopefully) forthcoming “APEX 2.0” version that will make use of a different, more user-friendly platform (e.g., based on Python), the present paper will hopefully be an appropriate guide to both APEX and the overall ecosystem in which it works. It is unfortunately not possible to install APEX and use it without reading this paper and the guide (*Readme.pdf*, which can be found in the .zip file of the Supplementary Materials). However, a full explanation of both the software functioning and the environment in which APEX should be used, will hopefully provide a powerful and flexible tool with which to study both the photochemical functioning of surface waters and the behavior of pollutants inside them. The contents of this publication are organized as follows:

- The main manuscript provides a general introduction to the software, its capabilities, and the way to use it. It does not replace the user’s guide, but it gives an initial taste of how the software works. Attention is also given to the experimental procedures that can be used to obtain the input data. These procedures are described in detail, as are the tips and tricks that enable modeling to be extended to stratified lakes and to rivers.
- *Readme.pdf* is the user’s guide, which explains how to install the software (which is provided in the .zip file of the Supplementary Materials), lists the equations of the photochemical model, and provides all the needed details on how to use APEX.

As an attempt to help users, input data concerning 44 water pollutants that have already been studied in recent years are also provided. A list of the available molecules and how to use the relevant data is given in the *Readme.pdf* file (see Supplementary Materials).

## 2. What APEX Is, What APEX Does

APEX is a tool to predict the phototransformation kinetics of pollutants in surface waters, and to study the photochemical behavior of sunlit aquatic systems. It is not standalone software, because it is based on the free Octave package [17]. New Octave versions can be downloaded at https://www.gnu.org/software/octave/. The Octave version for Windows that I personally use, and on which the software has been extensively tested, is the non-new 3.2.4 one. Finding this version online might not be totally straightforward, so it is provided in the form of a compressed file (*3.2.4_gcc-4.4.0.zip*) in the Supplementary Materials. My advice is to try this version first (see the *readme.pdf* file in the Supplementary Materials for detailed installation instructions), and to download a newer version only if the Octave 3.2.4 provided here does not work.

APEX was initially conceived to predict the phototransformation kinetics of pollutants in sunlit surface waters. The typical target at an early stage was to derive a 3D plot of the half-life time of a pollutant as a function of the water parameters that have the most impact on it, i.e., water depth and the dissolved organic carbon (DOC) [21]. The APEX results can be obtained directly in the form of a 3D plot (*Plotgraph* function) or in tabular form (*Savetable* function, which produces *.csv* files). The *Savetable* output data can be rearranged (e.g., to carry out additional calculations) and used as the basis for plots drawn with other software packages. A straightforward way to open and modify the output *.csv* files is to use Excel^®^ or equivalent software, paying attention to the column data separation options (the correct one is “comma separation” or equivalent).

APEX needs input data that can be divided into two different groups, namely environment-dependent variables and substrate-dependent variables. The former are the sunlight spectrum, the water absorption spectrum, water chemistry (which includes the photochemically most important parameters: [NO_3_^−^], [NO_2_^−^], [HCO_3_^−^], [CO_3_^2−^] and the DOC), water depth, and the quantum yields for the generation of ^•^OH, ^1^O_2_, and ^3^CDOM* from irradiated CDOM. The variables that depend on the substrate/pollutant are its absorption spectrum, the direct photolysis quantum yield, and the second-order reaction rate constants with ^•^OH, CO_3_^•−^, ^1^O_2_, and ^3^CDOM*. It is also possible to take into account the formation of a given intermediate, and in this case the relevant input variables are the intermediate formation yields from the substrate upon direct photolysis and reaction with ^•^OH, CO_3_^•−^, ^1^O_2_, and ^3^CDOM*.

**Figure 1:**
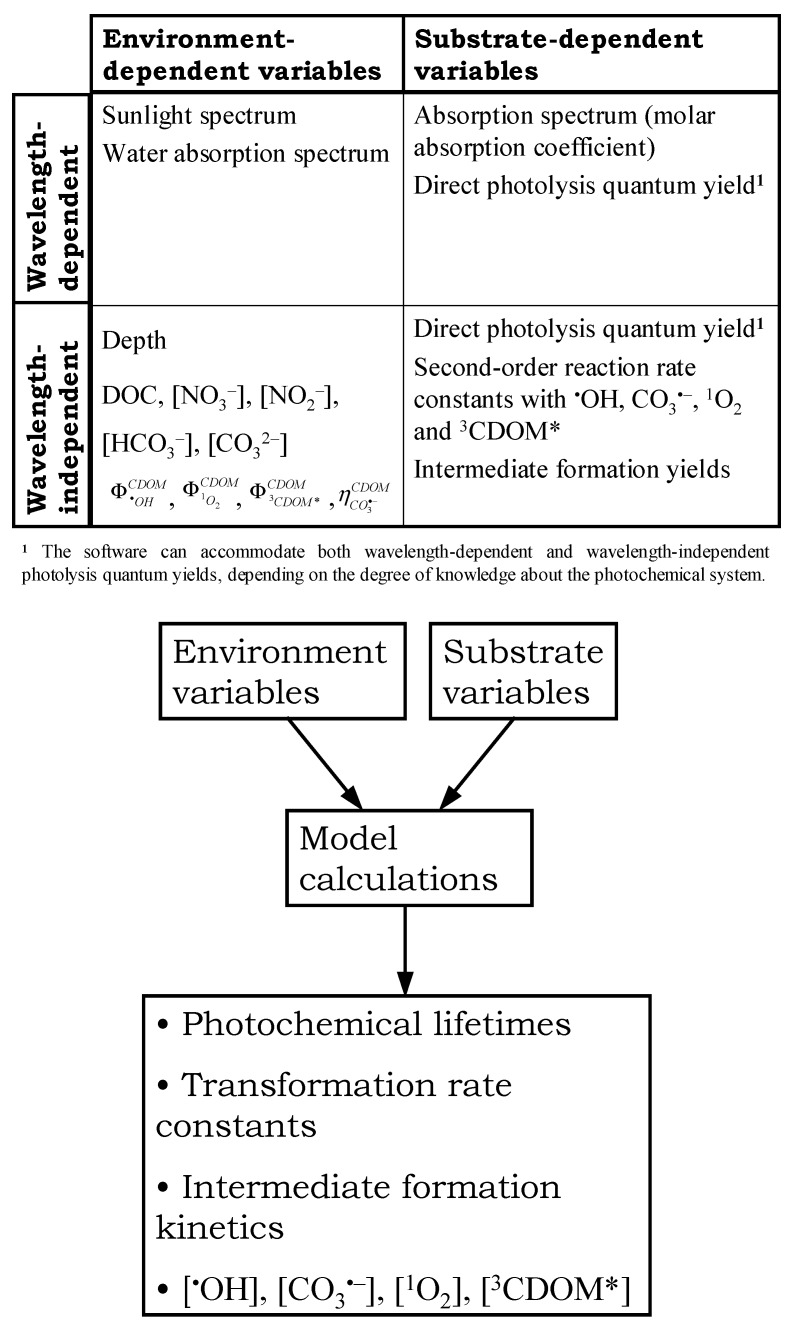
**Top:** Schematic of the input variables of the “aqueous photochemistry of environmentally occurring xenobiotics” APEX software, divided according to both the context they refer to (environment vs. molecule/substrate) and the wavelength dependence. **Bottom:** general block scheme that depicts how the APEX software works. The “substrate variables” can be neglected if one is only interested in the photochemical functioning of the environment. Note that $\Phi_{•OH}^{CDOM}$, $\Phi_{1^{}O_{2}}^{CDOM}$ and $\Phi_{{}^{3}CDOM*}^{CDOM}$ are the formation quantum yields of, respectively, ^•^OH, ^1^O_2_ and ^3^CDOM* from irradiated CDOM, while $\eta_{CO_{3}^{•-}}^{CDOM}$ describes the formation of CO_3_^•−^ from ^3^CDOM*.

It is noteworthy to observe that some of the above input variables are wavelength-dependent, while others are not. Therefore, a further subdivision into subclasses can be introduced as shown in Figure 1. This subdivision is mirrored in the way input data are introduced into the software, because the wavelength-dependent variables are accommodated in the *molecule.csv* input files (*molecule.csv* means that, by default, the file has the name of the substrate, e.g., *Ibuprofen.csv* or *Acetaminophen.csv*). In contrast, the wavelength-independent quantities are introduced in the *Plotgraph* or the *Savetable* functions.

All the wavelength-independent quantities can be introduced either as definite numerical values or as variables within a given range. Two such variables (X and Y) should be defined for each run, but it is also possible to restrict the variation range to a single numerical value if useful (i.e., to use a variable as if it were a constant). Based on these input data, the software computes a range of output variables. They are: (i) half-life times of the given molecule/pollutant, as well as the corresponding first-order degradation rate constants (relative to each single photochemical pathway, or to all the pathways pooled together); (ii) steady-state concentrations of ^•^OH, CO_3_^•−^, ^1^O_2_, and ^3^CDOM*; (iii) formation rate constants of an intermediate; and (iv) fractions of substrate transformation or intermediate formation accounted for by each pathway. It should be noted that the time unit in the output file is a sunny summer (fair-weather) day, equivalent to 15 July at 45° N latitude. This can be modified when changing the input sunlight spectrum in the *molecule.csv* file. In contrast, the steady-state values [^•^OH], [CO_3_^•−^], [^1^O_2_], and [^3^CDOM*] refer to a sunlight UV irradiance (290–400 nm) of 22 W m^−2^ on top of the water surface.

It is important to remark that the experimentally derived input parameter $\Phi_{{}^{•}OH}^{CDOM}$ = 3 × 10^−5^ (formation quantum yield of ^•^OH by irradiated CDOM), present as a default value in APEX, takes into account the photogeneration of ^•^OH by Fe species, including the photo-Fenton reactions [34,35]. Indeed, Fe photochemistry in surface waters largely depends on the behavior of the Fe–DOM complexes, while inorganic Fe species (and especially Fe colloids [36]) play a secondary role. A very precise knowledge of Fe speciation would thus be needed to predict the details of Fe-induced photoreactions, but this detailed knowledge is usually not available. Therefore, within APEX, Fe photochemistry leading to ^•^OH is accounted for within CDOM photoreactions.

Another issue to be considered is that the energy of photons is usually much higher than the thermal vibrational energy. For this reason, photoinduced reactions show lower temperature dependence compared with other, non-photoinduced processes [37], and the temperature effect is not taken into account here. Still, if second-order reaction rate constants are measured at different temperature values and found to be significantly different, they can be used as input data in different runs to assess a temperature effect. It is also of note that if the concentration of a pollutant is low enough, which is typically the case in surface waters, its photoreaction kinetics are independent of the concentration itself. For this purpose, APEX uses a default concentration [S]_o_ = 10^−8^ mol·L^−1^ (inserted in the *apex.m* calculation file) that is low enough to meet the above requirement.

Extensive use of the calculation engine first (the never publicly released Turbo Pascal-based version dates back to 2009), and of the full software later, has shown that APEX can be a powerful tool to model the photochemistry of aquatic systems and of the pollutants occurring in them. However, its potential can be exploited in full only if APEX is conceived as part of a whole environment, where kinetic measurements (to provide the input data) and photochemical modeling operate together [28,29,30]. Most photochemical degradation experiments carried out in the laboratory, even on real water samples, have the important drawback that it is not easy to extrapolate results obtained in the centimeters-deep water columns of photoreactors to the depths of several meters that are typical of natural water bodies [38]. This is the main reason why most experimental approaches tend to systematically overestimate direct photolysis over indirect photochemistry, thereby providing a distorted view of the way natural aquatic systems work. To my knowledge, APEX is the only tool that allows for easy extrapolation of laboratory results to the real environment, when both direct photolysis and indirect photochemistry are concerned. However, APEX requires second-order reaction rate constants as input data, and most laboratory experiments that can be conceived simply do not allow for these data to be obtained. This is the reason why, in addition to explaining how the software works, this paper also includes a detailed explanation of how experiments should be carried out to derive meaningful input data.

APEX was initially conceived to treat well-mixed and stationary systems, such as overturning lakes. Therefore, the default calculation scenario concerns a well-mixed system of this kind. However, expert users could also employ APEX to successfully carry out calculations in stratified lakes and in rivers, also taking into account the effect of pH [27]. The tips and tricks to carry out these calculations are provided in dedicated sections (Section 5 and Section 6).

## 3. Input Data

The APEX software requires as input data some quantities that depend on the environment and others that depend on the molecule. Moreover, there are two different places where input data can be inserted, namely the *Savetable* and *Plotgraph* files (to be used in alternative), as well as the *molecule.csv* spreadsheet(s). As preliminary remark, consideration should be made of the use of “−1” and “−2” in *Savetable/Plotgraph* versus *molecule.csv*. In the *molecule.csv* file, sometimes one may not want the software to read some columns, because priority should be given to calculations carried out on data reported elsewhere. This is especially true of the columns *phi* (wavelength trend of the photolysis quantum yield) and *Aw* (water absorption spectrum). To tell the software to ignore these columns, all their numerical values should be made equal to −1.

The meaning of −1 and −2 is totally different in *Savetable/Plotgraph*. Here, −1 is the way to define an X variable (e.g., by typing “CNO3 = −1;”, one defines nitrate as an X variable), while −2 defines a Y variable (e.g., “kP_OH = −2;” defines the ^•^OH reaction rate constant as a Y variable). Note that all the quantities present in *Savetable/Plotgraph* (concentration values of water parameters, water depth, second-order reaction rate constants, photolysis quantum yield, intermediate formation yields) can be defined as X or Y variables.

### 3.1. Environment-Dependent Variables

They may be inserted into either *Savetable/Plotgraph*, or *molecule.csv*.

#### 3.1.1. Savetable/Plotgraph

The environmental variables to be inserted into *Savetable/Plotgraph* are water depth and the concentration values of nitrate (mol L^−1^), nitrite (mol L^−1^), bicarbonate (mol L^−1^), carbonate (mol L^−1^), and DOC or NPOC (dissolved organic carbon or non-purgeable organic carbon, which are different notations to indicate the same parameter, units of mg_C_·L^−1^). The way such files look is shown in the user’s guide (*readme.pdf* Section 6, see Supplementary Materials).

In some cases, the value of the TOC may be available (total organic carbon, sum of DOC and POC, i.e., particulate organic carbon) instead of the DOC. In this case, the following observations apply: (i) organic matter in surface waters is prevalently found in its dissolved form, thus TOC ≈ DOC as a first approximation [39]; and (ii) the photochemistry of surface freshwaters is mainly a chemistry of dissolved species, with a small to negligible contribution from suspended matter [36,40]. Thus, in this context, the TOC is a decent approximation for the DOC.

The units of mg_C_·L^−1^ are the generally accepted measure units for organic matter. In contrast, in the case of nitrate and nitrite, it is customary to find data in units of $mg_{NO_{3}^{-}}\;L^{-1}$, $mg_{NO_{2}^{-}}\;L^{-1}$, or $mg_{N}\;L^{-1}$ (the latter also indicated as nitrate-N or nitrite-N). However, APEX needs data in molarity to carry out light-absorption calculations in a straightforward (Lambert–Beer-based) way. The conversion between different analytical units and the required mol·L^−1^ ones has to take into account the molecular weights of NO_3_^−^ (62,000 $mg_{NO_{3}^{-}}\;mol^{-1}$) and NO_2_^−^ (46,000 $mg_{NO_{2}^{-}}\;mol^{-1}$), as well as the equivalent weight of N (14,000 $mg_{N}\;mol^{-1}$).

The case of HCO_3_^−^ and CO_3_^2−^ may be more complex, because their concentration data are often not available (unless dedicated water titration is carried out, which is relatively rare). In contrast, alkalinity (*Alk*) and pH are more commonly found [26]. The procedure to derive [HCO_3_^−^] and [CO_3_^2−^] from *Alk* and pH is based on the thermodynamic theory of equilibria in aqueous solution [41,42]. It is explained in detail in the *Readme.pdf* file (Supplementary Materials, paragraph 3.10).

#### 3.1.2. Molecule.csv

The environmental variables that depend on the wavelength may be inserted into the *molecule.csv* input file if one needs to replace the default values (see Section 5 of *readme.pdf* in Supplementary Materials for insight as to how this kind of files looks; there is a different file for each molecule to be studied). The relevant variables are the sunlight spectrum (*p0sun*; the default spectrum is related to 45° N, and units are Einstein cm^−2^·s^−1^·nm^−1^) and the measured water absorbance (*Aw*, referred to 1 cm optical path length, i.e., cm^−1^ units). Note that if one needs to make calculations for mid-latitude conditions and does not want to use a specific water absorption spectrum, the values of *p0sun* and *Aw* present in the default *molecule.csv* files provided here work well. In this case, they do not need to be modified.

The default time unit used in APEX is a fair-weather day in mid-July at 45° N latitude, and the day–night cycle is taken into account. The APEX package can make a (approximated) post-calculation assessment of the seasonality of photochemical parameters (*APEX_season.xls* spreadsheet, see *readme.pdf* for this), but the default sunlight spectrum used in *molecule.csv* is referenced to the month of July at 45°N (at 9:00 or 15:00 solar time). This issue should be taken into account when modifying the default sunlight spectrum to consider, for example, different latitudes in calculations (which can be done successfully, see Reference [43]). Note that one dedicated APEX calculation must be carried out for each given latitude value, because it is not possible to define the latitude as a variable.

If no input value of *Aw* is given (i.e., the *Aw* column of *molecule.csv* has a −1 value overall, as in the default files provided), APEX computes the water absorption spectrum on the basis of the DOC (NPOC) value using the following formula [34] (note that the measure unit of A_1_(λ) is [cm^−1^]):

(12) $$A_{1}(\lambda )=0.45\times DOC\times \exp^{\left(-0.015\times \lambda \right)}$$

This approach is particularly useful if one wants to assess the environmental behavior of a compound as a function of varying DOC without having a particular environment in mind (or if, for a given water environment, the DOC value is available but the water absorption spectrum has not been measured). For the approach based on Equation (12) to be followed, the whole *Aw* column in the *molecule.csv* file should have the standard value −1.

In other cases, however, one might wish to assess the photochemical behavior of a compound in a particular environment, for which the absorbance of the water surface layer is available. The use of actual absorbance understandably increases the accuracy of the calculations. In this case, instead of −1, the *Aw* column should contain the experimental absorbance values measured at different wavelengths (note that in *molecule.csv* the wavelengths are not given in 1 nm increments, because the typically available sunlight spectra do not respect this requirement) over a 1 cm optical path length (or divided by the used path length in cm: the important issue is that cm^−1^ units are used for *Aw*). If the software finds different data than −1 in the *Aw* column of *molecule.csv*, these data are used with priority for light-absorption calculations.

### 3.2. Substrate-Related Variables

#### 3.2.1. Savetable/Plotgraph

The molecular variables that are required as input data in APEX, and that should be inserted within *Savetable/Plotgraph*, deal with photoreaction kinetics. In particular, they are the second-order reaction rate constants (units of L·mol^−1^·s^−1^) of the molecule with ^•^OH, CO_3_^•−^, ^1^O_2_, and ^3^CDOM*, the direct photolysis quantum yield (mol Einstein^−1^ units; because 1 Einstein = 1 mole of photons, it is not mistaken to consider it unitless), as well as (optional) the formation yields of an intermediate upon direct photolysis and upon reaction of the initial substrate with ^•^OH, CO_3_^•−^, ^1^O_2_, and ^3^CDOM*.

One comment should concern the direct photolysis quantum yield, because very often only one wavelength-averaged value is available. The latter can be inserted into *Savetable/Plotgraph* (*fi_P* variable) as a single numerical datum (e.g., “*fi_P* = 0.33;”). In some other cases, a full series of wavelength-dependent data may be available, which should be inserted into the *molecule.csv* file (*phi* column), where a wavelength trend can be easily arranged. 

In theory, a more straightforward option would have been to only foresee the insertion of the quantum yield value in *molecule.csv*, either as a constant value for all the wavelengths or as a wavelength trend. However, we judged it important to retain the possibility of defining the photolysis quantum yield as a X or Y variable in *Savetable/Plotgraph*. This possibility becomes very useful when the exact quantum yield is not known, but one still wants to see whether or not the direct photolysis process is significant compared to other photoreactions, making the quantum yield vary over a wide range of values [38] (this is actually an instance of the priority often given in APEX to flexibility over user-friendliness).

In practice, with the photolysis quantum yield one has the following options:

1. Insert the known wavelength trend in *molecule.csv* (column *phi*); in this case, the software will ignore the value of *fi_P* provided in *Savetable/Plotgraph*;
2. Insert the known constant value of *fi_P* (suppose it is 0.33, as for ibuprofen) in *Savetable/Plotgraph*; to make the software read this value with priority, the *phi* column in *molecule.csv* should be made equal to −1; note that the same result can be obtained by inserting the above constant value (0.33) in the whole *phi* column of *molecule.csv*, which will be read with priority;
3. Define the quantum yield as a variable (X, “*fi_P* = −1”, or Y, “*fi_P* = −2”) within *Savetable/Plotgraph*. In this case, it is mandatory to have −1 in the whole *phi* column of *molecule.csv*.

Similarly to the quantum yield, for all the parameters it is possible to give specific numerical values (e.g., “*kP_CO3* = 1e8;”, which sets $k_{P+CO_{3}^{•-}}$ = 10^8^ L·mol^−1^·s^−1^) or to define them as variables (e.g., “*kP_CO3* = −2;” to define $k_{P+CO_{3}^{•-}}$ as Y variable).

Concerning the intermediate formation yields, APEX can compute the formation kinetics of an intermediate if the relevant formation yields are known. An intermediate formation yield in a given photoreaction pathway is basically the ratio between the initial formation rate of the intermediate and the initial degradation rate of the substrate (vide infra for suggestions on the way to experimentally determine this). For instance, the formation yields of the toxic intermediate 4-isobutylacetophenone from the non-steroidal anti-inflammatory drug ibuprofen are as follows: 25% by direct photolysis, 2.3% upon ^•^OH reaction, 31% upon reaction with ^3^CDOM*, and no formation upon reaction with ^1^O_2_ or CO_3_^•−^. The yields to be introduced in APEX are fractions and not percentages; thus, the input data should read as follows: “*y_OH* = 0.023; *y_CO3* = 0; *y_1O2* = 0; *y_3DOM* = 0.31; *y_Phot* = 0.25;” (see *readme.pdf*, Supplementary Materials, paragraph 6.1.3).

The data input for the *Savetable* function is now over. The remaining default numerical values (formation quantum yields of ^•^OH, ^1^O_2_, and ^3^CDOM* from irradiated CDOM, formation parameter for CO_3_^•−^ from irradiated CDOM and CO_3_^2−^) should be replaced only if reliable substitutes are available (which means that a campaign of water-sample irradiation has been carried out on purpose). In many cases, the default values can be used as such.

In the case of *Plotgraph*, after selection of the X and Y variables, one should still choose which of the 36 possible output variables should be the Z value of the 3D plot. Possibilities include half-life times and first-order rate constants (pathway-specific, or including all the photochemical pathways together); steady-state concentrations ([^•^OH], [CO_3_^•−^], [^1^O_2_], [^3^CDOM*]) under a sunlight UV irradiance of 22 W·m^−2^; fractions of substrate transformation and intermediate formation accounted for by the different photoreaction pathways; and the relative roles of NO_3_^−^, NO_2_^−^, and CDOM as ^•^OH sources. The way this selection interface looks in *Plotgraph* is shown in *readme.pdf*, Supplementary Materials, paragraph 6.1.4. In the case of the *Savetable* function, such a selection is not necessary, because all the relevant data are always displayed in the output file. 

#### 3.2.2. Molecule.csv

As already explained, this file is used to arrange wavelength-dependent parameters. The typical one is the absorption spectrum of the compound (molar absorption coefficient, units of L mol^−1^ cm^−1^), which should be reported in the *EP* column (see Section 5 of *readme.pdf* in Supplementary Materials). Basically, it is the content of this column that customizes each *molecule.csv* file. As explained in Section 3.2.1, the direct photolysis quantum yield may be placed in the *phi* column of the file, but this is not the only option. Note that all the 44 default *molecule.csv* files provided here (see the “Compounds” folder of the Supplementary Materials) contain a wavelength-averaged value of the direct photolysis quantum yield in their relevant *phi* columns.

#### 3.2.3. Final Considerations Concerning the Input Data

The above text explains how to introduce the data concerning molecular variables into APEX, but it does not tell how to obtain these data. For all molecules for which APEX calculations have been carried out by the author’s group, input values (measured photolysis quantum yields and second-order reaction rate constants, see the file *readme.pdf*) and input files (of the kind *molecule.csv*) are provided as Supplementary Materials (there is a total of 44 such files). For some other molecules, data can be found in the literature (especially those concerning photolysis quantum yields and ^•^OH reaction rate constants). There is also a possibility to obtain the reaction rate constants with ^•^OH, ^1^O_2_, CO_3_^•−^, and ^3^CDOM* with a quantitative structure-activity relationship (QSAR) approach or known correlations, but the same is much more difficult for the photolysis quantum yields. In all other cases, the relevant parameters have to be measured experimentally. The following sections explain how to measure the photochemical kinetics parameters required by APEX (with two alternative approaches to determine the reaction rate constants) and how to approximately assess some of these values using QSAR.

## 4. Experimental Measurement of Photoreaction Parameters

It should be preliminarily observed that the paragraphs below were written with the use of liquid chromatography as analytical technique in mind, to monitor the degradation of the target pollutant, and UV-Vis spectrophotometry only to measure absorption spectra for light-absorption calculations. While different techniques can be and have actually been used (e.g., plate count for bacteria [44]), they are not necessarily validated or recommended. For instance, when using UV-Vis spectrophotometry to monitor the time evolution of a substrate (e.g., a dye), care should be taken to avoid spectral interference [45]. Therefore, full UV-Vis spectra should be taken instead of, for instance, carrying out single-wavelength monitoring.

Note also that in the following pages, definite concentration values (of photosensitizers, substrates and so on) will be reported in several cases. These values are only indicative, and are mentioned here because they have been found to work well under laboratory conditions that, however, cannot be necessarily replicated exactly in a different context. Therefore, corrections and optimizations will usually be needed. The reported conditions are thus only a starting point, and hopefully a useful time-saving suggestion for experimenters, but there is absolutely no guarantee that everything will work on the first attempt.

### 4.1. Direct Photolysis Quantum Yield

The direct photolysis of a compound can be assessed by monitoring its transformation under (natural or simulated) sunlight or under environmental UV radiation (λ > 300 nm). The photolysis quantum yield Φ is the ratio between the initial transformation rate of the compound (*R*_o_, units of mol·L^−1^·s^−1^) and its absorbed photon flux (*P*_a_, units of Einstein L^−1^·s^−1^) [46]. The absorbed photon flux can be calculated by knowing the incident spectral photon flux density *p*°(λ) of the light source, the optical path length *b* inside the photoreactor, the molar absorption coefficient ε(λ) of the compound undergoing the direct photolysis, and its concentration *c* (e.g., 20 µmol·L^−1^) [46]:

(13) $$P_{a}=\int_{\lambda_{1}}^{\lambda_{2}}p{}^{\circ}(\lambda )\;\;[1-10^{-\varepsilon (\lambda )\;b\;c}]\;d\lambda$$

The numerical integration should be carried out over the wavelength interval [λ_1_,λ_2_] where the emission spectrum of the lamp (*p*°(λ)) and the absorption spectrum of the compound (ε(λ)) overlap. The values of ε(λ) can be determined spectrophotometrically, while the determination of *p*°(λ) requires the combination of a fiber-optic spectrophotometric measurement with chemical actinometry (I often use the 2-nitrobenzaldehyde method [47], which is described in detail in the Supplementary Material of Marchisio et al. [48], but several alternatives are available. See, for instance, Kuhn et al. [49] for a review on chemical actinometry). An important consideration should be made when measuring absorption spectra in the context of direct photolysis quantum yield experiments; the usual tendency is to take the spectrum of a solution at a substrate concentration value that allows for a UV peak (often, a peak in the UVC region) to nicely stay on scale. In this way, a nice-looking absorption spectrum can be obtained. Unfortunately, however, one very often sees the absorbance decrease almost to zero when approaching the UVB region, where the absorption spectrum may get confounded with the background noise. With such data in hand, it may be difficult to tell exactly whether the substrate is unable to absorb sunlight, or whether its absorbance is just very low. The difference between the two scenarios may not be trivial; for instance, ibuprofen has very low (but non-negligible) absorbance above 290 nm which, combined with a high photolysis quantum yield (Φ = 0.33), ensures that the direct photolysis can account for over 50% of its phototransformation in some environmental conditions [19]. In order to exactly measure absorption above 290 nm, one should take the spectrum of a solution that is as concentrated as possible (in the case of ibuprofen, at least 0.01 mol·L^−1^ at circumneutral pH). In this case, any UVC absorption peak(s) may be totally saturated, but the environmental UV absorption can be properly highlighted.

Another issue is that the initial degradation rate *R*_o_ should take into account phototransformation alone. Dark experiments are thus needed to determine the hydrolysis kinetics and possible biotransformation (although unlikely in laboratory solutions), which should be subtracted from the measured phototransformation rate. The calculation of the initial reaction rates requires the knowledge of the time evolution of the substrate (*C*_t_ = concentration at time *t*, *C*_o_ = initial concentration, *k* = kinetic constant), in a first-order kinetic approximation ($C_{t}=C_{o}\;e^{-k\;t}$) or in a zero-order one (*C*_t_ = *C*_o_ (1 – *k t*)), followed in either case by *R*_o_ = *k C*_o_. When both *R*_o_ and *P*_a_ are known, one can calculate the direct photolysis quantum yield as $\Phi =R_{o}\;P_{a}^{-1}$.

### 4.2. Measurement of Second-Order Reaction Rate Constants without Using Reference Compounds

When using this approach, it is first necessary to photochemically produce the different reactive transient species. Recommended sources are H_2_O_2_ for ^•^OH, NO_3_^−^ + HCO_3_^−^/CO_3_^2−^ for CO_3_^•−^, Rose Bengal for ^1^O_2_, and 4-carboxybenzophenone (CDOM proxy) for ^3^CDOM* [20]. The choice of H_2_O_2_ as ^•^OH source is motivated by the fact that alternative sources (NO_3_^−^, NO_2_^−^) yield ^•^OH together with several other interfering species. If, for any reason, H_2_O_2_ cannot be used (e.g., in experiments of photoinactivation of bacteria or viruses, for which H_2_O_2_ is toxic [43,44]), NO_3_^−^ is a better choice than NO_2_^−^.

#### 4.2.1. Reaction with ^•^OH

The determination of the second-order reaction rate constant of a substrate *S* with ^•^OH should make use of an ^•^OH scavenger of known reactivity. It is not necessary to monitor the time evolution of the scavenger, which can be an alcohol such as methanol, ethanol, 2-propanol, or t-butanol. The latter two compounds are the most frequently used, presumably because of the low reactivity of the secondary radicals (interfering species) arising from the oxidation of the relevant alcohols by ^•^OH. The kinetic system should take into account the formation of ^•^OH by H_2_O_2_ photolysis (if possible under UVB radiation, which is experimentally more convenient than UVC), as well as the reactions of ^•^OH with H_2_O_2_, the added alcohol, and the substrate *S* [50]:

(14) $$H_{2}O_{2}\;+\;h\nu \;\to \;2{\;}^{•}\mathrm{OH}\;[R_{•\mathrm{OH}}[$$

H_2_O_2_ + ^•^OH → HO_2_^•^ + H_2_O  [*k*_15_ = 2.7 × 10^7^ L·mol^−1^·s^−1^] (15)

Alcohol + ^•^OH → Products  [*k*_16_] (16)

*S* + ^•^OH → Products  [*k*_17_] (17)

where $R_{•\mathrm{OH}}$ is the formation rate of ^•^OH upon photolysis of H_2_O_2_. Upon application of the steady-state approximation to ^•^OH, one gets the following trend for the initial transformation rate of *S*, *R*_S_:

(18) $$R_{S}=R_{•\mathrm{OH}}\frac{k_{17}[S]}{k_{15}[H_{2}O_{2}]+k_{16}[Alcohol]+k_{17}[S]}$$

The most straightforward operation is to study the transformation of *S* at constant [S] and [H_2_O_2_], letting the initial concentration of the alcohol vary. Suggested concentration values are 20 µmol·L^−1^ for *S*, and H_2_O_2_ fixed somewhere between 10^−3^ and 10^−2^ mol·L^−1^, letting the alcohol vary in different irradiation experiments, from 0 to 0.01 or 0.1 mol·L^−1^ (the higher the *k*_17_, the more concentrated should be the alcohol to inhibit the degradation of *S*). Note that the added alcohol at the highest concentration value should cause *R*_S_ to decrease by at least 50% compared to the case where no alcohol is present. By plotting the experimental data of *R*_S_ as a function of [Alcohol], one can use Equation (18) as a fit function with $R_{•\mathrm{OH}}$ and *k*_17_ as fit parameters (*k*_15_, *k*_16_, [S], and [H_2_O_2_] are known; note that *k*_16_ = 9.7 × 10^8^ L·mol^−1^·s^−1^ for methanol, 1.9 × 10^9^ L·mol^−1^·s^−1^ for both ethanol and 2-propanol, and 6.0 × 10^8^ L·mol^−1^·s^−1^ for t-butanol [50]). The two fit parameters are mutually orthogonal; thus, the fit result is univocal. As an alternative, and especially if one lacks a non-linear fit procedure, Equation (18) can be linearized as follows:

(19) $$\frac{1}{R_{S}}=\frac{1}{R_{•\mathrm{OH}}}+\frac{k_{15}[H_{2}O_{2}]+k_{16}[Alcohol]}{R_{•\mathrm{OH}}\;k_{17}[S]}=\left(\frac{1}{R_{•\mathrm{OH}}}+\frac{k_{15}[H_{2}O_{2}]}{R_{•\mathrm{OH}}\;k_{17}[S]}\right)+\frac{k_{16}[Alcohol]}{R_{•\mathrm{OH}}\;k_{17}[S]}$$

In this case, one should plot 1/*R*_S_ vs. [Alcohol] and make a linear fit, deriving *k*_17_ from the slope and intercept of the fit line.

#### 4.2.2. Reaction with CO_3_^•−^

In the case of the carbonate radical, it is not possible to determine the second-order reaction rate constant without competition kinetics. However, it is possible to carry out a screening study by which to see whether the CO_3_^•−^ reaction could be important in the photodegradation of the given compound in natural waters. The rationale is that CO_3_^•−^ can be generated upon irradiation of nitrate in the presence of bicarbonate [51]:

NO_3_^−^ + hν → [O^•−^ + ^•^NO_2_]_cage_ (20)

[O^•−^ + ^•^NO_2_]_cage_ → NO_3_^−^ (21)

[O^•−^ + ^•^NO_2_]_cage_ + H^+^ → ^•^OH + ^•^NO_2_ (22)

HCO_3_^−^ + ^•^OH → CO_3_^•−^ + H_2_O (23)

CO_3_^2−^ + ^•^OH → CO_3_^•−^ + OH^−^ (24)

HCO_3_^−^ + [O^•−^ + ^•^NO_2_]_cage_ → CO_3_^•−^ + OH^−^ + ^•^NO_2_ (25)

CO_3_^2−^ + [O^•−^ + ^•^NO_2_]_cage_ + H_2_O → CO_3_^•−^ + 2 OH^−^ + ^•^NO_2_ (26)

The photolysis of nitrate yields O^•−^ + ^•^NO_2_, which are initially contained in a solvent cage, i.e., surrounded by water molecules. The two radical species can either undergo in-cage (geminate) recombination to nitrate (Equation (21)), or diffuse into the solvent bulk, where they can react with other species (bulk recombination of ^•^OH + ^•^NO_2_ is very unlikely). It has been shown that in the presence of nitrate alone under irradiation, more that 75% of O^•−^ + ^•^NO_2_ undergoes geminate recombination, while only 25% diffuses into the solution bulk [52]. The addition of excess scavengers (in this case, HCO_3_^−^ + CO_3_^2−^) can trigger the consumption of both the bulk species (especially ^•^OH) and, most notably, the in-cage ones, thereby inhibiting geminate recombination and increasing the overall formation yield of radicals (^•^OH + ^•^NO_2_ + CO_3_^•−^). For instance, excess alcohol addition as an ^•^OH scavenger, coupled with monitoring of the alcohol transformation products as ^•^OH probes, can result in an over-prediction of the nitrate photolysis quantum yield [53].

The addition of bicarbonate salts at sufficiently high concentration values can also inhibit geminate recombination, because of the scavenging of cage O^•−^ (reactions 25,26). This process enhances the production of CO_3_^•−^ and, because of the lower geminate recombination of [O^•−^ + ^•^NO_2_]_cage_, the overall yield of ^•^OH + CO_3_^•−^ with nitrate + bicarbonate (usually with a prevalence of CO_3_^•−^, because most ^•^OH is scavenged in either the cage or the bulk) is higher than the ^•^OH yield with nitrate alone [51].

The above phenomenon can have two consequences, depending on the reactivity of a substrate with ^•^OH vs. CO_3_^•−^: if the substrate reacts only with ^•^OH, with poor or no CO_3_^•−^ reactivity, its degradation is inhibited by bicarbonate (note that less ^•^OH occurs with nitrate + bicarbonate compared to nitrate alone). If the CO_3_^•−^ reactivity is significant, the addition of bicarbonate enhances degradation because of the increased production of ^•^OH + CO_3_^•−^ compared to the case of nitrate alone [51]. However, the interpretation of the experimental results may be a bit less straightforward because: (i) bicarbonate addition to nitrate increases pH, and some compounds might undergo a reactivity change with pH, e.g., because of deprotonation; moreover, (ii) direct photolysis may be important, which could complicate the analysis of the bicarbonate trend. Indeed, in some cases the CO_3_^•−^ reaction might not show up well enough against the background of direct phototransformation.

To tackle the above possible biases, three series of experiments should be carried out [19]:

1. Irradiation of the substrate (e.g., at 20 μmol L^−1^) in the presence of nitrate (e.g., 10^−2^ mol·L^−1^) and different HCO_3_^−^ values (e.g., 0 to 10^−2^ mol·L^−1^).
2. Irradiation of the substrate with nitrate at the same concentrations as before, but with a phosphate buffer (H_2_PO_4_^−^ + HPO_4_^2−^) instead of bicarbonate. The phosphate buffer should have the same total concentration as the bicarbonate salt, and the same pH within 0.2–0.3 units.
3. Irradiation of the substrate at the usual concentration without nitrate, but with the same bicarbonate concentrations as in series (1) (direct photolysis check).

If the substrate degradation rates in series (3) are sufficiently lower than those in (1) and (2), then the direct photolysis is not so important and will not mask the other photochemical pathways. In this case, two possible scenarios can be in effect [51]:

(a) Transformation with bicarbonate is faster than that with phosphate: in this case, the CO_3_^•−^ reaction could be important and should be investigated, e.g., by measuring the reaction rate constant with competition kinetics (vide infra);

(b) Transformation with bicarbonate is slower than that with phosphate: in this case, the CO_3_^•−^ reaction is likely to be negligible in surface-water conditions.

#### 4.2.3. Reaction with ^1^O_2_

The generation of ^1^O_2_ can make use of Rose Bengal (RB) under irradiation as a suitable photosensitizer. The recommended RB concentration is somewhere around 10 µmol L^−1^. This dye absorbs visible light; thus, selective irradiation can conveniently use a yellow lamp that will minimize the probability of directly photolyzing the substrate *S*. The resulting kinetic system is quite simple (ISC = inter-system crossing, while superscripts ^1^ and ^3^ indicate singlet and triplet excited states, respectively) [54]:

(27) $$\mathrm{RB}\;+\;h\nu \;\to {\;}^{1}\mathrm{RB}*\;\overset{ISC}{\to }{\;}^{3}\mathrm{RB}*\;\overset{O_{2}}{\to }\;\mathrm{RB}\;+{\;}^{1}O_{2}\;[{R_{1}}_{O_{2}}]$$

^1^O_2_ → O_2_  [*k*_28_ = 2.5 × 10^5^ s^−1^] (28)

*S* + ^1^O_2_ → Products  [*k*_29_] (29)

Upon application of the steady-state approximation to ^1^O_2_, one gets the following equation:

(30) $$R_{S}={R_{1}}_{O_{2}}\frac{k_{29}[S]}{k_{28}+k_{29}[S]}$$

where *R*_S_ is again the initial transformation rate of S. If [S] is low enough, the above equation can be linearized as follows: $R_{S}={R_{1}}_{O_{2}}k_{29}k_{28}^{-1}[S]$. From the linear plot of *R*_S_ vs. [S] one can derive *k*_29_ because *k*_28_ is known, provided that the value of ${R_{1}}_{O_{2}}$ is also known. To determine ${R_{1}}_{O_{2}}$, it is recommended to use furfuryl alcohol (FFA) as a probe and carry out another irradiation experiment with the same RB concentration and irradiation set-up as before. In this case, the kinetic system is made up of Equations (27) and (28) plus the following [55]:

FFA + ^1^O_2_ → Products [*k*_31_ = 1 × 10^8^ L·mol^−1^·s^−1^] (31)

By applying again the steady-state approximation to ^1^O_2_, the initial degradation rate of FFA gets the following expression, which allows for ${R_{1}}_{O_{2}}$ to be determined:

(32) $$R_{FFA}={R_{1}}_{O_{2}}\frac{k_{31}[FFA]}{k_{28}+k_{31}[FFA]}\;\Rightarrow \;{R_{1}}_{O_{2}}=R_{FFA}\frac{k_{28}+k_{31}[FFA]}{k_{31}[FFA]}$$

The plot *R*_S_ = *m* [S], which has a linear form if [S] is low enough, and where *m* is the line slope, can then be arranged as follows:

(33) $$m=R_{FFA}\frac{k_{29}(k_{28}+k_{31}[FFA])}{k_{28}k_{31}[FFA]}\;\Rightarrow \;k_{29}=m\frac{k_{28}k_{31}[FFA]}{R_{FFA}(k_{28}+k_{31}[FFA])}$$

It is thus possible to derive *k*_29_ based on known parameters.

#### 4.2.4. Reaction with ^3^CDOM*

The determination of the second-order reaction rate constant with reactive triplet states is conceptually more complicated, because CDOM is not a definite chemical species, and the same holds for ^3^CDOM*. Dissolved compounds interact with triplet states with a mix of physical quenching and chemical reactivity, both of which consume ^3^CDOM* (usually yielding CDOM, CDOM^•−^, or CDOMH^•^ depending on the process). However, these processes do not necessarily modify the compounds themselves. The quenching rate constant describes the sum of physical quenching and actual reactions, and it can be determined by laser flash photolysis using either proxy molecules (the triplet states of which simulate the behavior of ^3^CDOM*) or, more recently, actual CDOM [56,57]. Unfortunately, the quenching rate constants are only upper limits for the reaction rate constants that are involved in the actual transformation of the substrates.

An alternative strategy to laser flash photolysis is the use of irradiation experiments with CDOM proxy molecules. Here, the conditions are more stringent compared to the laser flash photolysis experiments, because the photochemistry and photophysics of the proxy molecule have to be very well known (indeed, liquid chromatography tells nothing about the triplet-state kinetics). Anthraquinone-2-sulfonate (AQ2S) was the first proxy molecule to be proposed, due to its well-known photophysics and straightforward triplet-state reactivity without side reactions such as the generation of ^•^OH or ^1^O_2_ [58]. Unfortunately, the triplet state ^3^AQ2S* is considerably more reactive than typical ^3^CDOM*; therefore, the use of AQ2S produces overestimations of triplet-sensitized reactions in the environment. More recently, 4-carboxybenzophenone (CBBP) has been proposed as a proxy for irradiation experiments, because the reactivity of ^3^CBBP* is more similar to that of ^3^CDOM* [20]. The procedure to be used for CBBP experiments is described below [59].

Solutions containing CBBP (suggested concentration around 50–100 µmol L^−1^) and the substrate *S* (variable concentration values in different experiments, typically below 100 µmol L^−1^) are irradiated under a UVA lamp. The initial degradation rates of *S*, *R*_S_’, are then calculated for different initial values of [S]. The direct photolysis of *S* is checked by irradiating it alone (i.e., without CBBP) under the same lamp and at the same concentration values as before, obtaining the initial photolysis rates *R*_S_”. For each *S* concentration value, the “genuine” triplet-sensitized transformation rate is determined as *R*_S_ = *R*_S_’ − χ *R*_S_”, where χ is the ratio between the photon flux absorbed by *S* in a CBBP solution, compared to the photon flux absorbed in a solution that contains *S* alone (χ takes into account the light-screening effect of CBBP on *S*). Note that if *S* does not undergo direct photolysis under UVA radiation, one simply has *R*_S_ = *R*_S_’. Otherwise:

(34) $$\chi =\frac{\int_{\lambda_{1}}^{\lambda_{2}}\left(p{}^{\circ}(\lambda )\frac{\varepsilon_{S}(\lambda )[S]}{\varepsilon_{S}(\lambda )[S]+\varepsilon_{CBBP}(\lambda )[CBBP]}[1-10^{-b\left\{\varepsilon_{S}(\lambda )[S]+\varepsilon_{CBBP}(\lambda )[CBBP]\right\}}]\right)\;d\lambda }{\int_{\lambda_{1}}^{\lambda_{2}}\left(p{}^{\circ}(\lambda )[1-10^{-b\varepsilon_{S}(\lambda )[S]}]\right)\;d\lambda }$$

where *p*°(λ) is the lamp spectral photon flux density, the concentrations [S] and [CBBP] refer to the initial values, ε is a molar absorption coefficient, *b* the optical path length in the irradiated photoreactor, and [λ_1_,λ_2_] is the wavelength interval where the emission spectrum of the lamp overlaps with the absorption spectrum of S.

In the solution containing CBBP and *S*, CBBP undergoes excitation to ^3^CBBP* that can [60]: (i) undergo thermal deactivation (which includes physical quenching by *S* and reaction-less encounters with dissolved O_2_); (ii) react with O_2_ to produce ^1^O_2_, which could contribute to *S* degradation (by comparison, AQ2S has the advantage of not producing ^1^O_2_ when irradiated); (iii) react with *S* and promote its transformation. The experiments are carried out at different initial values of [S] and, if [S] is low enough, a linear plot is obtained of *R*_S_ = *m* [S] (which means that *S* at low concentration is a negligible ^3^CBBP* quencher). The application of the full kinetic model (see [60] for details) yields the following expression for the reaction rate constant between *S* and ^3^CBBP* ($k_{S+{}^{3}CBBP*}$, units of L·mol^−1^·s^−1^):

(35) $$k_{S+{}^{3}CBBP*}=k\prime \;\left(\frac{m}{P_{a,CBBP}}-\frac{0.68\;S_{\Delta }\;k_{29}}{k_{28}}\right)=6\times 10^{5}\frac{m}{P_{a,CBBP}}-0.75\;\;k_{29}$$

where *k*’ = 6 × 10^5^ s^−1^ is the first-order inactivation rate constant of ^3^CBBP* in aerated solution, *S*_Δ_ = 0.46 is the ^1^O_2_ yield between ^3^CBBP* and O_2_ (which means that 46% of the ^3^CDOM* + O_2_ encounters end up in CDOM + ^1^O_2_, and 54% in CDOM + O_2_), while *k*_28_, *k*_29_ respectively describe ^1^O_2_ inactivation and its reaction with *S*, as per the previous section [60]. *P*_a,CBBP_ is the photon flux absorbed by CBBP, the calculation of which should take into account the possible competition for lamp irradiance between *S* and CBBP. The general equation is the following:

(36) $$P_{a,CBBP}=\int_{\lambda_{1}}^{\lambda_{2}}\left(p{}^{\circ}(\lambda )\frac{\varepsilon_{CBBP}(\lambda )[CBBP]}{\varepsilon_{S}(\lambda )[S]+\varepsilon_{CBBP}(\lambda )[CBBP]}[1-10^{-b\left\{\varepsilon_{S}(\lambda )[S]+\varepsilon_{CBBP}(\lambda )[CBBP]\right\}}]\right)\;d\lambda$$

If (ε_S_(λ) × [S]) « (ε_CBBP_(λ) × [CBBP]), the above equation is simplified as follows:

(37) $$P_{a,CBBP}=\int_{\lambda_{1}}^{\lambda_{2}}\left(p{}^{\circ}(\lambda )[1-10^{-b\varepsilon_{CBBP}(\lambda )[CBBP]}]\right)\;d\lambda$$

**Back-reduction processes.** The triplet-state reactivity might not end up like this, however. Canonica and co-workers have in fact shown that the antioxidant (mostly phenolic) moieties of DOM can inhibit the triplet-sensitized degradation of contaminants. The mechanism of action concerns the primary intermediates generated by the electron-transfer or H-abstraction oxidation of a substrate *S* by ^3^CDOM*. These intermediates (S^•+^ or (S-H)^•^, the latter representing *S* minus a hydrogen atom) can be reduced back to *S* by the DOM phenolic moieties (Ph–OH), following the reaction scheme below [61,62,63]:
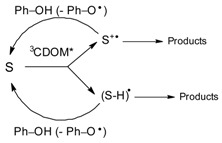
(38)

Assume *R*_S_° as the triplet-sensitized transformation rate *of S* without antioxidants, *R*_S_ the rate in the presence of antioxidants, [Ph–OH] the concentration of phenolic antioxidant moieties, and [Ph–OH]_1/2_ the concentration that produces *R*_S_ = 0.5 *R*_S_°. The experimental trend of *R*_S_ vs. [Ph–OH] can be usually expressed as follows [63]:

(39) $$R_{S}=R_{S}^{o}\frac{1}{1+\frac{\left[Ph-OH\right]}{{\left[Ph-OH\right]}_{1/2}}}$$

Therefore, [Ph–OH]_1/2_ can be obtained by numerical fit of the experimental data. As an alternative, the linearized form is $1/R_{S}=(1/R_{S}^{o})+[Ph-OH]\times {\left({\left[Ph-OH\right]}_{1/2}\times R_{S}^{o}\right)}^{-1}$. Phenol is a suitable model antioxidant that can be used in this kind of experiment, e.g., by irradiating CBBP + *S* in the presence of different phenol concentration values (a possible suggestion to start with could be [Ph–OH] = 0–10 µmol·L^−1^). Note that the reaction rate *R*_S_° is made up of the contributions of K_s+3CBBP_*, [^3^CBBP*], and [S] (*R*_S_ = $k_{S+{}^{3}CBBP*}$ [^3^CBBP*] [S], as per second-order reaction kinetics, which becomes pseudo-first order by assuming that ^3^CBBP* is in steady state). Therefore, the reaction rate *R*_S_ in the presence of antioxidants can be expressed as follows:

(40) $$R_{S}=\frac{k_{S+{}^{3}CBBP*}\;[{}^{3}CBBP*]\;[S]}{1+\frac{\left[Ph-OH\right]}{{\left[Ph-OH\right]}_{1/2}}}$$

By defining an apparent reaction rate constant, $k_{S+{}^{3}CBBP*}^{a}=k_{S+{}^{3}CBBP*}^{}(1+{\left\{\left[Ph-OH\right]/{\left[Ph-OH\right]}_{1/2}\}\right)}^{-1}$, one can write *R*_S_ in a shorter form:

*R*_S_ = $k_{S+{}^{3}CBBP*}^{a}$ [^3^CBBP*] [S]. The value of $k_{S+{}^{3}CBBP*}^{a}$ can be used in APEX to carry out photochemical modeling (vide infra for the details), but natural DOM contains phenolic moieties and not actual phenol. The phenolic equivalent of DOM should thus be considered, and recent studies suggest that:

⇒ If [Ph–OH] is expressed in µmol L^−1^ and DOC_1/2_ in mg_C_·L^−1^, the following equivalence holds: DOC_1/2_ = 0.4 [Ph–OH]_1/2_ [64].

With this definition, and using CBBP as CDOM proxy, the apparent reaction rate constant between *S* and ^3^CDOM* ($k_{S+3^{}CDOM*}^{a}$) can be expressed as follows:

(41) $$k_{S+{}^{3}CDOM*}^{a}=\frac{k_{S+{}^{3}CBBP*}}{1+\frac{DOC}{DOC_{1/2}}}$$

**Figure 2:**
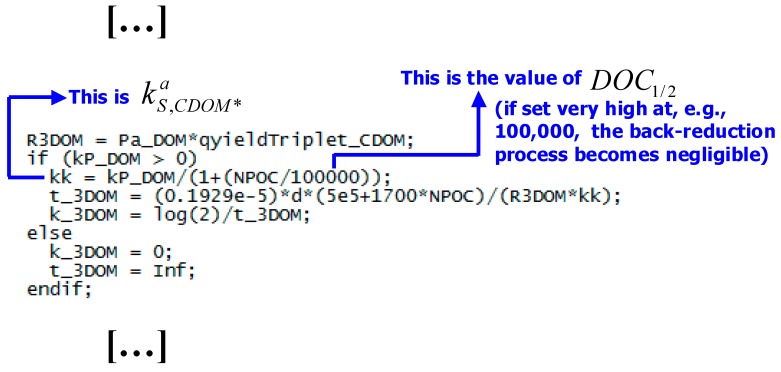
This is the part of the *apex.m* file where the back-reduction process can be described. The default value of DOC_1/2_ is so high (100,000) that back-reduction is negligible in the as-provided file. If modeling of back-reduction is needed, a suitable DOC_1/2_ numerical value (in mg_C_·L^−1^ units) should replace the default 100,000. For instance, for the acid and basic forms of sulfadiazine it is DOC_1/2_ = 17 and 0.7 mg_C_·L^−1^, respectively [64]. It is advisable to restore the 100,000 value after use, to avoid inadvertent back-reduction modeling when it is not needed. Note that DOC and NPOC are synonyms.

The following step is then to modify the APEX code in order to accommodate DOC_1/2_ in the expression for $k_{S+{}^{3}CBBP*}^{a}$. In this case, modification should involve the *apex.m* calculation engine (see Figure 2 for instructions).

### 4.3. Measurement of Second-Order Reaction Rate Constants by Using Reference Compounds (Competition Kinetics)

The experimental procedures described above have several pros, including robustness; indeed, it is relatively easy in that context to identify unwanted reactions (e.g., the direct photolysis) and carry out due corrections. For instance, to account for direct photolysis or additional processes in the case of ^•^OH, it is sufficient to fit the experimental data of *R*_S_ vs. [Alcohol] with a modified form of Equation (18), where a constant additive term is introduced in the right-hand side [65]. Nevertheless, the aforementioned procedures have a considerable disadvantage in being lengthy, because several experiments have to be carried out to derive a single reaction rate constant. This issue limits the number of compounds for which the photochemical reactivity can be assessed, which is a problem when considering the number of new chemicals that reach the market every year.

A faster procedure (competition kinetics) implies the use of reference compounds, combined with the monitoring of the time evolution of both the substrate and the reference compound, which should both be present at the same time in the same solution [66]. The advantage is that, in this case the second-order reaction rate constant may be obtained with just one irradiation experiment (although it is highly advisable to carry out replicates). Moreover, competition kinetics has turned out to be a necessary choice when studying the photoinactivation of bacteria and viruses, e.g., to avoid the use of toxic alcohols as ^•^OH scavengers [44,67]. In that case, and always for the issue of toxicity, it was also necessary to choose nitrate as ^•^OH source instead of H_2_O_2_. Unfortunately, the competition kinetics approach works well if there is only one operational reaction pathway. Indeed, it may be relatively difficult to account for additional reactions and exclude their interference, except perhaps for direct photolysis.

For a description of how competition kinetics works [68], assume *S* as the substrate for which one wants to determine the second-order reaction rate constant with the transient X, *k*_S+X_ (with X = ^•^OH, CO_3_^•−^, ^1^O_2_, or ^3^CDOM*). The transients can be customarily generated upon irradiation of suitable photosensitizers, e.g., H_2_O_2_ (^•^OH), NO_3_^−^ + HCO_3_^−^ (CO_3_^•−^), Rose Bengal (^1^O_2_), and CBBP (^3^CBBP* as ^3^CDOM* proxy). Assume *R* as the reference molecule, with a known second-order reaction rate constant *k*_R+X_. The solution to be irradiated should contain the photosensitizer to produce X (e.g., 1 to 10 mmol L^−1^ H_2_O_2_ under UVB, 10 mmol L^−1^ NO_3_^−^ + 100 mmol L^−1^ HCO_3_^−^ under UVB, 10 µmol L^−1^ Rose Bengal under yellow light, 50 to 100 µmol L^−1^ CBBP under UVA), as well as a mixture of *S* and *R*. Things are easier if the two compounds are used at the same initial concentration, i.e., [S]_o_ = [R]_o_, which can be somewhere in the range of 5–20 µmol L^−1^. During irradiation, the time evolution of both *S* and *R* should be monitored. A pseudo-first order degradation kinetics is usually observed, of the kind [S]_t_ = [S]_o_ $e^{-k_{S}t}$ and [R]_t_ = [R]_o_ $e^{-k_{R}t}$, where *t* is time and *k*_S_ and *k*_R_ the first-order degradation rate constants of *S* and *R*, respectively. The initial degradation rates are thus *R*_S_’ = *k*_S_ [S]_o_, and *R*_R_’ = *k*_R_ [R]_o_. Another experiment should involve irradiation of an *S* + *R* mixture, again with [S]_o_ = [R]_o_ and under the same lamp as before, but in the absence of photosensitizers, to assess the direct photolysis kinetics of the two compounds. With a similar first-order kinetics approach, one gets *R*_S_” and *R*_R_” as the initial degradation rates of, respectively, *S* and *R* by direct photolysis. If *S* and/or *R* directly photolyze in this experiment, it means that the runs with the photosensitizer actually provide a combination of direct photolysis and indirect photochemistry. It is thus not correct to just subtract the direct photolysis rate from the degradation rate measured in the presence of the photosensitizer, in order to obtain the rate of the photosensitized process after accounting for the direct photolysis. In fact, the direct photolysis kinetics may be slowed down in the solution containing the photosensitizer, because the latter absorbs radiation and inhibits the direct photolysis of the substrates (i.e., *R* and *S*) [46].

Provided that additional processes different from direct photolysis and the X reaction are not operational, the correct approach to subtract the direct photolysis kinetics is the following: the degradation rates of *S* and *R* in the photosensitizer solution, accounted for by reaction with the transient X are, respectively, *R*_S_ = *R*_S_’ − χ_S_ × *R*_S_”, and *R*_R_ = *R*_R_’ − χ_R_ × *R*_R_” (the meaning of *R*’ and *R*” is the same as before). The fractions χ_S_ and χ_R_ represent how much the direct photolysis kinetics of, respectively, S and R are slowed down in a solution that contains the photosensitizer *P*. These fractions can be calculated as follows, where *p*°(λ) is the lamp spectral photon flux density, ε a molar absorption coefficient, and *b* the optical path length in the photoreactor:

(42) $$\chi_{S}=\frac{\int_{\lambda_{1}}^{\lambda_{2}}\left(p{}^{\circ}(\lambda )\frac{\varepsilon_{S}(\lambda )\;{\left[S\right]}_{o}}{\varepsilon_{S}(\lambda )\;\;{\left[S\right]}_{o}+\varepsilon_{R}(\lambda )\;\;{\left[R\right]}_{o}+\varepsilon_{P}(\lambda )\;\;{\left[P\right]}_{o}}\;[1-10^{-b\;\{\varepsilon_{S}(\lambda )\;\;{\left[S\right]}_{o}+\varepsilon_{R}(\lambda )\;\;{\left[R\right]}_{o}+\varepsilon_{P}(\lambda )\;\;{\left[P\right]}_{o}\}}]\right)d\lambda }{\int_{\lambda_{1}}^{\lambda_{2}}\left(p{}^{\circ}(\lambda )\frac{\varepsilon_{S}(\lambda )\;{\left[S\right]}_{o}}{\varepsilon_{S}(\lambda )\;\;{\left[S\right]}_{o}+\varepsilon_{R}(\lambda )\;\;{\left[R\right]}_{o}}\;[1-10^{-b\;\{\varepsilon_{S}(\lambda )\;\;{\left[S\right]}_{o}+\varepsilon_{R}(\lambda )\;\;{\left[R\right]}_{o}\}}]\right)d\lambda }$$

(43) $$\chi_{R}=\frac{\int_{\lambda_{3}}^{\lambda_{4}}\left(p{}^{\circ}(\lambda )\frac{\varepsilon_{S}(\lambda )\;{\left[R\right]}_{o}}{\varepsilon_{S}(\lambda )\;\;{\left[S\right]}_{o}+\varepsilon_{R}(\lambda )\;\;{\left[R\right]}_{o}+\varepsilon_{P}(\lambda )\;\;{\left[P\right]}_{o}}\;[1-10^{-b\;\{\varepsilon_{S}(\lambda )\;\;{\left[S\right]}_{o}+\varepsilon_{R}(\lambda )\;\;{\left[R\right]}_{o}+\varepsilon_{P}(\lambda )\;\;{\left[P\right]}_{o}\}}]\right)d\lambda }{\int_{\lambda_{3}}^{\lambda_{4}}\left(p{}^{\circ}(\lambda )\frac{\varepsilon_{S}(\lambda )\;{\left[R\right]}_{o}}{\varepsilon_{S}(\lambda )\;\;{\left[S\right]}_{o}+\varepsilon_{R}(\lambda )\;\;{\left[R\right]}_{o}}\;[1-10^{-b\;\{\varepsilon_{S}(\lambda )\;\;{\left[S\right]}_{o}+\varepsilon_{R}(\lambda )\;\;{\left[R\right]}_{o}\}}]\right)d\lambda }$$

Note that [λ_1_,λ_2_] is the spectral interval where the spectrum of *S* overlaps with that of the lamp, and [λ_3_,λ_4_] is the interval where the *R* spectrum overlaps with the lamp.

After subtraction of the direct photolysis, one has *R*_S_ = *k*_S+X_ [S]_o_ [X] and *R*_R_ = *k*_R+X_ [R]_o_ [X]. The reaction solution is the same, thus [X] is the same for both compounds. Moreover, because [S]_o_ = [R]_o_, one has *R*_S_ (*R*_R_)^−1^ = *k*_S+X_ (*k*_R+X_)^−1^. The reaction rate constant between *S* and X can thus be obtained as follows [68]:

(44) $$k_{S+X}=k_{R+X}\frac{R_{S}}{R_{R}}=k_{R+X}\frac{R_{S}’-\chi_{S}R_{S}"}{R_{R}’-\chi_{S}R_{R}"}$$

In the case of ^•^OH, CO_3_^•−^, and ^1^O_2_, this is the end of the story. In the case of ^3^CDOM*, the back-reduction process should still be accounted for using the same procedure described in the previous section.

The choice of the reference compound *R* depends on several issues. First of all, a sound measurement of *k*_S+X_ can be carried out if 0.1 < *k*_S+X_ (*k*_R+X_)^−1^ < 10, otherwise the experimental reaction rates become too different. One then needs to know ${}^{3}CDOM*$ to be able to measure $k_{S+{}^{3}CBBP*}$. Unfortunately, the ^3^CBBP* reaction rate constant is presently known for only a few compounds, which restricts the number of reference molecules that can be used with CBBP. On this basis, acetaminophen and atrazine can be recommended as reference compounds because of their known $k_{R+{}^{3}CBBP*}$ [59]. In the case of CO_3_^•−^, it might be sometimes convenient to choose tyrosine [65], while acesulfame K can be used to study reactivity with ^•^OH and ^1^O_2_ [68]. The latter compound has the disadvantage of being ionic; thus, its monitoring by reverse-phase liquid chromatography requires the addition of an ionic coupler (e.g., tetrabutylammonium hydrogen sulfate) to the aqueous eluent. On the other hand, acesulfame K has the big advantage of not undergoing direct photolysis. Table 1 summarizes the known reaction rate constants of the mentioned compounds with the relevant transient species. For help in the choice of further reference compounds, it may be useful to look at literature lists of second-order reaction rate constants with ^•^OH [50,69], CO_3_^•−^ [70], and ^1^O_2_ [71].

### 4.4. QSAR Approaches to Determining the Second-Order Reaction Rate Constants

Compared to experimental measurements, a faster, although much less accurate method to determine the second-order reaction rate constants between reactive transient species and organic substrates makes use of QSAR calculations. Based on the molecular structure or on molecular characteristics such as the one-electron oxidation potential, it is possible to predict the reactivity with photogenerated transients with an accuracy that is usually within an order of magnitude of the target value. While not exceedingly accurate, such an approach can be used for an assessment of the photoreaction kinetics as a first screening, to identify particularly photostable or photolabile compounds that might be subject to further investigation. In the case of the direct photolysis, some studies have found a correlation between the photolysis quantum yield and parameters such as the energies of the HOMO and LUMO orbitals, as well as their difference. Moreover, other parameters that are strongly structure-dependent are also important [72,73]. For instance, in the case of polychlorodibenzodioxins, the number of chlorine atoms, the chlorine charges, and the dipole moment play a major role [73]. Unfortunately, such parameters are difficult or even impossible to extend to different structures.

A QSAR assessment of the second-order ^•^OH reaction rate constants with gas-phase compounds ($k_{S+•OH,gas}$) can be obtained using the shareware software EPI Suite^TM^ by US-EPA [74]. Interestingly, a correlation has also been observed between the gas-phase reaction rate constants and the aqueous-phase ones ($k_{S+•OH,\mathrm{water}}$), which can be expressed as follows depending on the measurement units of $k_{S+•OH,gas}$ (cm^3^ molecule^−1^ s^−1^ or L·mol^−1^·s^−1^, note that EPI Suite^TM^ uses the former) [8]:

(45) $$k_{S+•OH,\mathrm{water}}[L\;mol^{-1}s^{-1}]={\left(0.35\times {Ν}_{A}\times \;k_{S+•OH,\mathrm{gas}}[cm^{3}molecule^{-1}s^{-1}]\right)}^{0.76}$$

(46) $$k_{S+•OH,\mathrm{water}}[L\;mol^{-1}s^{-1}]={\left(350\times \;k_{S+•OH,\mathrm{gas}}[L\;mol^{-1}s^{-1}]\right)}^{0.76}$$

Note that N_A_ is Avogadro’s number (6.02 × 10^23^). One possible approach is thus to determine $k_{S+•OH,gas}$ with EPI Suite^TM^, and derive $k_{S+•OH,\mathrm{water}}$ with Equation (45). With ibuprofen this procedure yielded $k_{\mathrm{IBU}+•OH,\mathrm{water}}$ = 2.6 × 10^9^ L·mol^−1^·s^−1^, which is within one order of magnitude of the experimental value (1 × 10^10^ L·mol^−1^·s^−1^) [19].

The second-order reaction rate constants with CO_3_^•−^, ^1^O_2_, and ^3^CDOM* [L·mol^−1^·s^−1^] correlate reasonably well with the one-electron oxidation potential of the substrate, *E*_1_, coherently with the oxidizing nature of the transient species. The following correlations have been reported for phenolic compounds [75]:

(47) $$k_{S+1^{}O_{2}}=(7.8\times 10^{9})\times 10^{2.46\;E_{1}}$$

(48) $$k_{S+{CO}_{3}^{•-}}=(5.6\times 10^{9})\times 10^{1.54\;E_{1}}$$

(49) $$k_{S+{}^{3}CDOM*}=(2\times 10^{12})\times 10^{3.3\;E_{1}}$$

**Figure 3:**
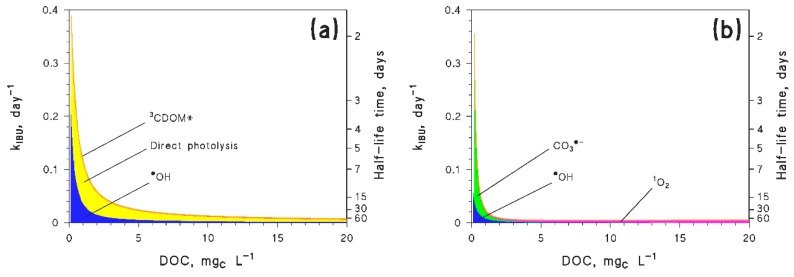
Modeling of the photodegradation kinetics of ibuprofen as a function of water DOC, carried out by using (**a**) the experimental photoreactivity data [19], and (**b**) the data obtained with the QSAR approaches described in the text. Other water conditions: 10^−4^ mol·L^−1^ NO_3_^−^, 10^−6^ mol·L^−1^ NO_2_^−^, 10^−3^ mol·L^−1^ HCO_3_^−^, 10^−5^ mol·L^−1^ CO_3_^2−^, 5 m water depth. Raw data were obtained with *Savetable* and further processed. The time unit is fair-weather days in mid-July, at 45°N latitude.

The ibuprofen radical cation has a reduction potential of 1.6 V vs. NHE [76], which corresponds to an oxidation potential for ibuprofen of *E*_1_ = −1.6 V (note that the reduction process S^•+^/S is understandably favored because of the instability of S^•+^, and the reverse happens for oxidation). With this datum, one gets $k_{IBU+1^{}O_{2}}$ = 9 × 10^5^ L·mol^−1^·s^−1^, $k_{IBU+CO_{3}^{•-}}$ = 1.9 × 10^7^ L·mol^−1^·s^−1^, and $k_{IBU+{}^{3}CDOM*}$ = 1 × 10^7^ L·mol^−1^·s^−1^. It should be remarked that, ibuprofen not being a phenolic compound, the estimates cannot be too accurate; the ^1^O_2_ reaction rate constant is overestimated by over an order of magnitude (15 times to be precise), there is unfortunately no reference for $k_{IBU+CO_{3}^{•-}}$, while $k_{IBU+{}^{3}CDOM*}$ is better predicted (within a factor of 5). Experimental data for comparison are reported in the file *readme.pdf* (Table 1) of the Supplementary Materials. Figure 3 compares the ibuprofen reaction pathways obtained by using the experimental (3a) vs. the predicted rate constants (3b). The former neglect the CO_3_^•−^ process, while the latter do not take into account direct photolysis. The figure gives some insight into the possible level of agreement between the experimental data and the QSAR approach. 

The agreement in the overall degradation kinetics looks surprisingly good for both low (<1 mg_C_·L^−1^) and relatively high (>10 mg_C_·L^−1^) DOC values. The main differences can be seen at intermediate DOC, but they are lower than an order of magnitude. Still, the QSAR approach does not consider the direct photolysis and overestimates CO_3_^•−^; it appears that the relatively good agreement in the overall kinetics stems from a compensation of errors, because the considered photoreaction pathways are definitely not the same, although they yield relatively similar kinetics.

### 4.5. Measurement of Intermediate Formation Yields

Knowledge of the intermediate formation yields, i.e., the ratios between intermediate initial formation rates and substrate initial degradation rates in the different photochemical pathways (direct photolysis and reaction with ^•^OH, CO_3_^•−^, ^1^O_2_, and ^3^CDOM*), is essential to predict the intermediate photogeneration kinetics by using APEX [17]. In these experiments, the substrate *S* should be irradiated alone or in the presence of photosensitizers (H_2_O_2_, NO_3_^−^ + HCO_3_^−^, RB, CBBP), to monitor the time evolution of both *S* and the intermediate(s) of interest (hereinafter *I*) [21]. This approach is usually allowed if the intermediate is available as a commercial standard, or if it is synthesized on purpose. In order to better quantify *I*, it might be convenient to use somewhat higher [S]_o_ values (e.g., 0.1 mM) compared to the previously described experiments dealing with degradation kinetics. The transformation of *S* into different intermediates, among which the intermediate *I*, followed by the transformation of *I* itself in a first-order kinetic context, can be described by the scheme below:
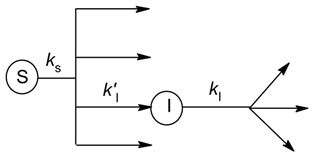
(50)
where *k*_S_ and *k*_I_ are the respective (first-order) degradation rate constants of *S* and *I*, and *k’*_I_ is the formation rate constant of *I* from *S* (obviously, *k*’_I_ ≤ *k*_S_). The first-order kinetic model yields the usual time evolution for *S* ([S]_t_ = [S]_o_ $e^{-k_{S}\;t}$, where the initial degradation rate is *R*_S_ = *k*_S_ [S]_o_). The time evolution of *I* is described by the following equation [77]:

(51) $${\left[I\right]}_{t}=\frac{k{’}_{I}{\left[S\right]}_{o}}{k_{S}-k_{I}}(e^{-k_{I}\;t}-e^{-k_{S}\;t})$$

**Figure 4:**
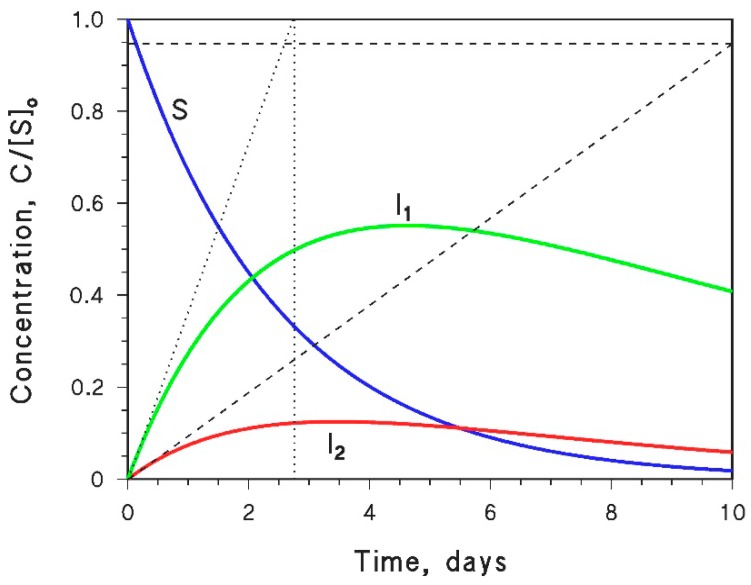
Calculation procedure for the initial formation rates of the intermediates I_1_ and I_2_, produced upon S transformation, using the initial slope (graphical) technique. The initial formation rate of I_1_ is {R_1_ [mol L^−1^ s^−1^]} ≅ {[S]_o_ [mol L^−1^]} (2.75 days × 3600 × 24)^−1^. The initial formation rate of I_2_ is {R_2_ [mol L^−1^ s^−1^]} ≅ 0.95 {[S]_o_ [mol L^−1^]} (10 days × 3600 × 24)^−1^.

The initial formation rate of *I* is *R’*_I_ = *k*’_I_ [S]_o_. However, the numerical fit of Equation (51), by making use of several free-floating and non-orthogonal parameters (at least *k*_I_ and *k’*_I_, and maybe also *k*_S_ and [S]_o_ to get a better fit), ensures that the fit outcome is non-univocal; different combinations of the floating parameters can give the same or very similar fit(s), and *k*’_I_ may be affected by a large error. Therefore, the calculation of *R’*_I_ by using a purely numerical approach may not be reliable, and it can be more convenient to determine *R’*_I_ graphically, as shown in Figure 4.

Briefly, the following procedure should be carried out: (i) irradiate *S* under the appropriate conditions (one photochemical pathway at a time) and monitor the time evolution of both *S* and *I*; (ii) determine R_S_; (iii) fit the experimental data of [I]_t_ vs. *t* with Equation (51), trying different initial values until you get a good fit (it might be necessary to use all four free-floating parameters for this purpose, i.e., *k*_I_, *k’*_I_, *k*_S_, and [S]_o_); (iv) draw the tangent of [I]_t_ vs. *t* at *t* = 0; (v) determine *R’*_I_ as the slope of the tangent line. Points (iv) and (v*)* are described in Figure 4.

Finally, once *R’*_I_ and *R*_S_ are known, the formation yield of *I* can be obtained as *y*_I_ = *R’*_I_ (*R*_S_)^−1^. The yield thus obtained refers to the single photochemical pathway under study, and *y*_I_ values should be determined for all the relevant photoreactions (direct photolysis, ^•^OH, CO_3_^•−^, ^1^O_2_, and ^3^CDOM*) [77].

## 5. How to Quantify the pH Effect

The solution pH can be an important parameter for photochemical reactions, especially if the substrate undergoes acid–base equilibrium within a pH interval that is significant for surface waters. One such example is represented by the solar filter benzophenone-4 (2-hydroxy-4-methoxybenzophenone-5-sulfonic acid, hereinafter BP4), which has a pK_a_ = 7.3 and is mainly photodegraded by direct photolysis and ^•^OH reaction [78]. The two forms of BP4 (acidic and basic) are indicated here as HBP4 and BP4^−^, respectively. The acid–base equilibrium can be expressed as follows, where *c*_BP4_ = [HBP4] + [BP4^−^]:

HBP4 ⇆ H^+^ + BP4^−^ (52)

(53) $$K_{a}=\frac{\left[H^{+}]\;[BP4^{-}\right]}{\left[HBP4\right]}$$

(54) $$[HBP4]=\alpha_{HBP4}c_{BP4}=\frac{\left[H^{+}\right]}{[H^{+}]+K_{a}}c_{BP4}=\frac{10^{-pH}}{10^{-pH}+10^{-pK_{a}}}c_{BP4}$$

(55) $$[BP4^{-}]=\alpha_{BP4^{-}}c_{BP4}=\frac{K_{a}}{[H^{+}]+K_{a}}c_{BP4}=\frac{10^{-pK_{a}}}{10^{-pH}+10^{-pK_{a}}}c_{BP4}$$

With the APEX software, it is possible to separately compute the photochemical transformation kinetics of both HBP4 and BP4^−^ (overall degradation rate constants, as well as rate constants accounted for by the single photoreaction pathways). In the case of the overall degradation rate constants, one gets $k_{HBP4}$ and $k_{BP4^{-}}$ from two separate APEX simulations (for the input data, see the file *readme.pdf*, Table 1, in the Supplementary Materials). The two results can be combined to obtain the overall BP4 transformation rate constant, $k_{BP4}^{overall}$, as follows [38,78]:

(56) $$k_{BP4}^{overall}=k_{HBP4}\alpha_{HBP4}+k_{BP4^{-}}\alpha_{BP4^{-}}=\frac{10^{-pH}k_{HBP4}+10^{-pK_{a}}k_{BP4^{-}}}{10^{-pH}+10^{-pK_{a}}}$$

**Figure 5:**
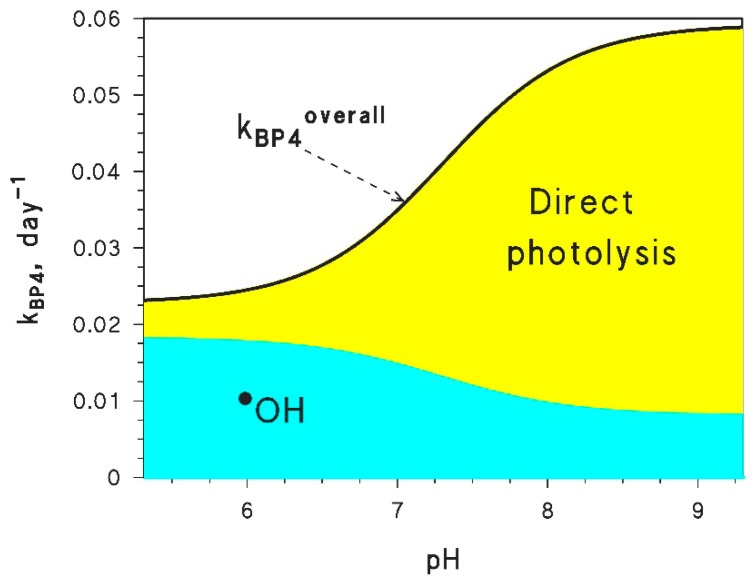
Modeled phototransformation kinetics of 2-hydroxy-4-methoxybenzophenone-5-sulfonic acid (BP4) (overall first-order rate constants, and role of the different photoreaction pathways), as a function of pH. Other water conditions: 10^−4^ mol·L^−1^ NO_3_^−^, 10^−6^ mol·L^−1^ NO_2_^−^, 10^−3^ mol·L^−1^ HCO_3_^−^, 10^−5^ mol·L^−1^ CO_3_^2−^, 3 mg_C_·L^−1^ DOC, 5 m water depth. Raw data were obtained with *Savetable* and further processed.

A similar approach can be followed for the degradation rate constants relative to the single photoreaction pathways, so that the pH trend can be derived for both the overall process (Equation (56)) and the separate pathways (^•^OH and direct photolysis). The calculations pertaining to the pH trend are not automatically carried out by APEX; thus, they should be performed separately on the APEX output data (clearly, the *Savetable* function should be used to produce such data, using the output *.csv* table as basis for the additional calculations). By following this approach, one can obtain the results shown in Figure 5. The pH trend is accounted for by the fact that HBP4 reacts with ^•^OH faster than BP4^−^, but the latter species undergoes much faster direct photolysis.

## 6. Model Treatment of Non-Thoroughly Mixed or Flowing Systems

The default use of APEX concerns stationary but thoroughly mixed systems, such as a lake during overturn, which are conceptually the simplest. However, lakes can become stratified during the summer season [79] while rivers flow by definition; thus, the most straightforward mathematical treatment of the reaction kinetics neglects some very important aquatic environments. Recently, tools and strategies have been developed to adapt the APEX output or input data to the treatment of somewhat more complex systems, such as stratified lakes, lakes undergoing evaporative concentration, and rivers [27]. The ways to do this, including tips on how to modify the code if needed, are reported below.

### 6.1. Stratified Lakes

**Figure 6:**
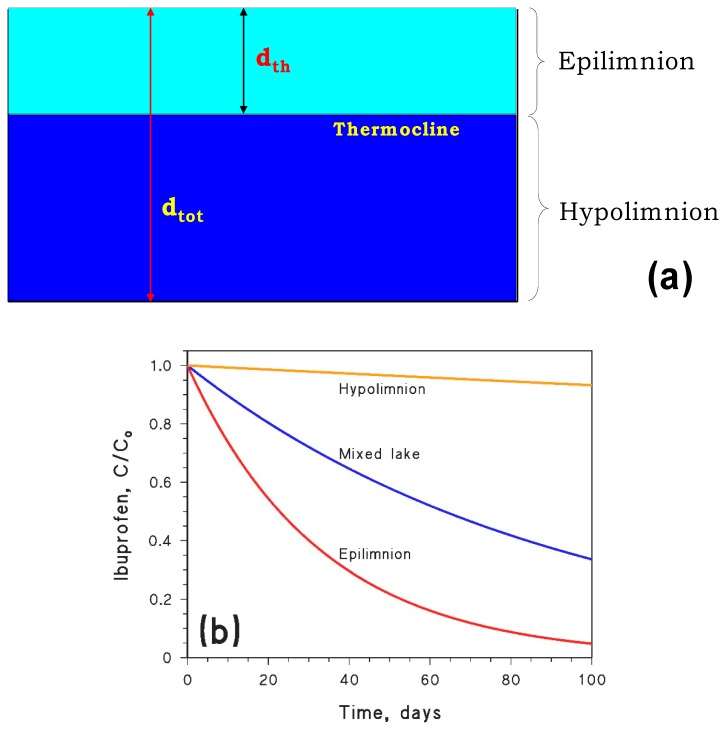
(**a**) Schematic of a stratified lake. (**b**) Modeled time trends of ibuprofen in the hypolimnion, the epilimnion, and the overall lake during overturn. Water conditions: 10^−4^ mol·L^−1^ NO_3_^−^, 10^−6^ mol·L^−1^ NO_2_^−^, 10^−3^ mol·L^−1^ HCO_3_^−^, 10^−5^ mol·L^−1^ CO_3_^2−^, 2 mg_C_·L^−1^ DOC, *d*_th_ = 10 m, *d*_tot_ = 30 m. Raw data were obtained with *Savetable* and further processed.

Lakes in temperate areas can undergo stratification during both summer and winter, but summer stratification is much more important from a photochemical point of view. In a stratified lake, the warmer and sunlit epilimnion floats over the darker and cooler hypolimnion, while the thermocline (i.e., the zone with the highest temperature gradient with depth) acts as a separation layer (see Figure 6a) [80]. In the absence of water mixing, the epilimnion and hypolimnion evolve separately, and so do the photochemical reactions in the two compartments. Photoprocesses are faster in the epilimnion than in the average or mixed lake, while the reverse is true in the hypolimnion [27]. The sunlight irradiance and the sunlight spectrum incident on the epilimnion (i.e., the lake surface) are taken into account by the default *p0sun* column of the *molecule.csv* input file. In contrast, because the epilimnion separates the hypolimnion from the surface, the incident irradiance on the hypolimnion is decreased by the absorption of radiation in the overlying water.

Assume *p*^epi^(λ) as the sunlight spectral photon flux density incident over the epilimnion/lake surface at a given wavelength λ. Although sunlight meets the water surface at an angle, refraction deviates the light trajectory in water towards the vertical [12]. Therefore, one makes a relatively low error by considering that the light path in water is equal to the water depth. As an alternative, one can introduce a correction factor *ψ* that is a function of latitude and season. The *ψ* parameter takes into account the fact that the path traveled by light in water is slightly longer than the water depth (see for this the *Readme.pdf* file in the Supplementary Materials, where paragraph 6.3 explains how to estimate *ψ*) [17]. The attenuation of sunlight in the epilimnion can be taken into account as follows:

(57) $$p^{th}(\lambda )=p^{epi}(\lambda )\times 10^{-[A_{1}(\lambda )\times d_{th}]}$$

where *p*^th^(λ) is the spectral photon flux density of sunlight at the depth of the thermocline, which separates epilimnion and hypolimnion; A_1_(λ) is the water absorbance over a path length of 1 cm (units of cm^−1^); and *d*_th_ (which may be replaced by *ψ* × *d*_th_) is the depth of the thermocline in cm. Note that consistent length units have to be used, so that the product A_1_(λ) × *d*_th_ is unitless.

A standard procedure to carry out the calculations of hypolimnion photochemistry could be to assume the default *p0sun* values as *p*^epi^(λ), apply Equation (57) to derive *p*^th^(λ), and insert the latter values into the *p0sun* column for the modeling of the hypolimnion. In practice, to carry out APEX calculations for the epilimnion, hypolimnion, and the overturning (mixed) lake, one simply has to modify the *p0sun* column of the *molecule.csv* file, and set the water depth, as follows:

**Epilimnion**: *p*^epi^(λ) → *p0sun*, depth *d* = *d*_th_

**Hypolimnion**: *p*^th^(λ) → *p0sun*, depth *d* = *d*_tot_ - *d*_th_

**Mixed lake**: *p*^epi^(λ) → *p0sun*, depth *d* = *d*_tot_

For more precision, the depth values can be multiplied by the *ψ* correction factor, as outlined above. Figure 6b reports the modeled time trends of ibuprofen in epilimnion, hypolimnion, and an overall mixed lake. The reported trends reflect the fact that the epilimnion is better illuminated than the overall lake due to its shallower water, while the hypolimnion is more poorly illuminated because of light screening by the overlying water column.

### 6.2. Rivers

The water flow in rivers is definitely a major parameter as far as the importance of photochemical reactions is concerned [27]. The (average) flow velocity $\bar{\nu }$ (units of [m s^−1^]) is a key issue, which is much more important for photochemistry when compared with the flow rate (*Q*, units of [m^3^ s^−1^]), although the latter is more often available [81]. Assume *L* as the length of a river: the residence time of flowing water is τ = L ($\bar{\nu }$)^−1^, which defines the timescale within which the photochemical reactions have to operate to be significant (note that water might pass to the underlying aquifer, but in this case it will be protected from light exposure. Photoreactions only occur during the in-stream time spent by both water, and the substrates it carries). Photoprocesses have fewer chances to be important under conditions of high flow, where $\bar{\nu }$ is also high and τ becomes correspondingly low. More interestingly, the reverse happens in periods of low flow. Therefore, photoreactions may help depollute rivers [27] when low values of *Q* and $\bar{\nu }$ could exacerbate pollution problems because of low dilution [82].

The flow rate *Q* = $\bar{\nu }$ *d w*, where *d* is the average depth and *w* the average width. It is reasonable to assume that a variation in *Q* will be evenly distributed amongst the three directions of space: in other words, if the water flow increases, one expects the water to flow faster and the river to be both deeper and wider (note that the width variation holds for natural water bodies, not for artificial channels where *w* might be constrained) [27]. In the case of even spatial distribution, and assuming ${\bar{\nu }}_{o}$, *d*_o_, *w*_o_ and *Q*_o_ to be the initial values of, respectively, average flow velocity, depth, width and water flow, we get $\bar{\nu }$ (${\bar{\nu }}_{o}$)^−1^ = *d* (*d*_o_)^−1^ = *w* (*w*_o_)^−1^ = $\sqrt[{3}]{Q\;{\left(Q_{o}\right)}^{-1}}$. Varying width does not affect the photoreaction rates, while the photoinduced processes are faster in shallower water. Moreover, slow water flow provides more time for the degradation reactions to take place. On this basis, given the pseudo-first order degradation rate constant *k*_S_ for a given substrate S, one can define both the half-life time (*t*_1/2_)_S_ = ln 2 (*k*_S_)^−1^ and the half-life length (*l*_1/2_)_S_ = $\bar{\nu }$ (*t*_1/2_)_S_ = ln 2 $\bar{\nu }$ (*k*_S_)^−1^. The parameter (*l*_1/2_)_S_ is the length of the river that is required for photoreactions to halve the concentration of *S* [27].

**Figure 7:**
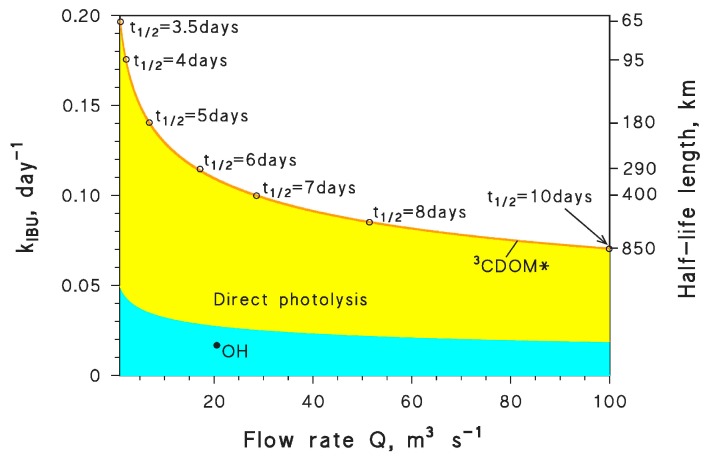
Modeled phototransformation kinetics of ibuprofen in river water, as a function of the flow rate Q (varied from 1 to 100 m^3^ s^−1^). Water conditions: 10^−4^ mol·L^−1^ NO_3_^−^, 10^−6^ mol·L^−1^ NO_2_^−^, 10^−3^ mol·L^−1^ HCO_3_^−^, 10^−5^ mol·L^−1^ CO_3_^2−^, 3 mg_C_·L^−1^ DOC, Q_o_ = 100 m^3^ s^−1^, *d*_o_ = 4 m. Raw data were obtained with *Savetable* and further processed.

Figure 7 shows an example of the application of this procedure to the attenuation of ibuprofen in river water. It was initially assumed that *Q*_o_ = 100 m^3^ s^−1^, with ${\bar{\nu }}_{o}$ = 1 m s^−1^, *d*_o_ = 4 m, and *w*_o_ = 25 m. It was then assumed that *Q* gradually decreased 100-fold, down to 1 m^3^ s^−1^. Assuming *d* (*d*_o_)^−1^ = $\sqrt[{3}]{Q\;{\left(Q_{o}\right)}^{-1}}$, we ultimately find *d* = 0.86 m. Therefore, APEX (*Savetable* function) was set to study the degradation of ibuprofen with varying depth from 0.86 to 4 m at 1 cm steps (“*d* = −1;”, “*x* = 0.86:0.01:4;”). The resulting values of the first-order degradation rate constants in the different photoprocesses (columns *k_OH*, *k_1O2*, *k_3DOM*, *k_Phot* of the output file, neglecting the CO_3_^•−^ reaction) were then plotted as shown in Figure 7 as a function of *Q*. The values of *Q* to be used were derived from the corresponding values of *d* that were available in the output file, as *Q* = *Q*_o_ [*d* (*d*_o_)^−1^]^3^. For selected cases (highlighted in the figure), the values of the half-life times (column *t_tot* in the output file) were chosen, and the corresponding *l*_1/2_ values were computed. Because *l*_1/2_ = $\bar{\nu }$ *t*_1/2_ and $\bar{\nu }$ (${\bar{\nu }}_{o}$)^−1^ = *d* (*d*_o_)^−1^, the half-life length was obtained as follows:

(58) $$l_{1/2}\;=\;{\bar{\nu }}_{o}\;d\;(d_{o}){-}^{1}\;t_{1/2}$$

As anticipated, it can be seen that photoreactions are both faster and have more time to take place if water flows more slowly. Therefore, photochemistry can be considered an important process in river water during drought.

### 6.3. Aquatic Systems under Evaporative Concentration

Lakes, ponds, and other stationary water systems located in subtropical areas can undergo evaporative concentration during the dry/hot season(s), especially if other water loss mechanisms are not or poorly operational [83,84]. The basic idea is that water evaporates but non-volatile solutes do not; thus, the concentration values of NO_3_^−^, NO_2_^−^, HCO_3_^−^, CO_3_^2−^ and the DOC all increase with decreasing water volume and depth [27]. If there are no constraints on the way the water volume decreases upon evaporation, one can make the hypothesis that the volume contraction is operational along the three axes. The most important quantities are volume (*V*) and depth; thus, *d* (*d*_o_)^−1^ = $\sqrt[{3}]{V\;{\left(V_{o}\right)}^{-1}}$ (similar equations hold for the horizontal widths, while for the water surface *S* it is *S* (*S*_o_)^−1^ = [*V* (*V*_o_)^−1^]^2/3^, but they have no photochemical implications).

**Figure 8:**
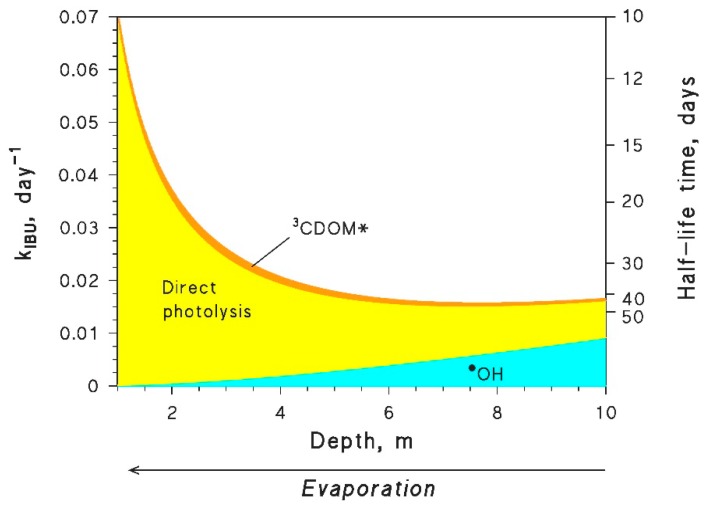
Trends of the first-order degradation rate constant of ibuprofen (left Y axis) and of the corresponding half-life time (right Y axis), as a function of water loss due to evaporative concentration. The weights of the different photoreaction pathways are highlighted in different colors. Initial water conditions for *d*_o_ = 10 m: 10^−4^ mol·L^−1^ NO_3_^−^, 10^−6^ mol·L^−1^ NO_2_^−^, 10^−3^ mol·L^−1^ HCO_3_^−^, and 10^−5^ mol·L^−1^ CO_3_^2−^. Raw data were obtained with *Savetable* and further processed.

Therefore, if depth gradually decreases from, say, *d*_o_ = 10 m down to 1 m (see Figure 8), the concentration values of photosensitizers and scavengers (hereafter [℘J]) are expected to vary as follows:

[℘] = [℘]_o_ [*d*_o_ (*d*)^−1^]^3^ (59)

**Figure 9:**
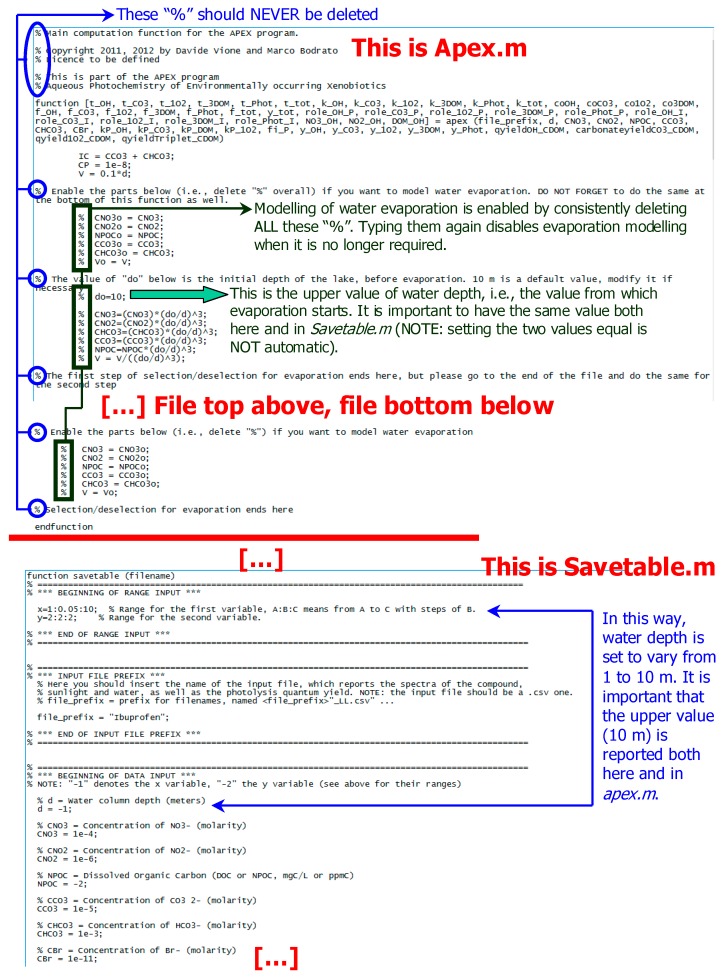
This figure reports the initial and final parts of *apex.m* (**top**) as well as a middle part of *Savetable.m* (**bottom**), showing the changes that should be made to *apex.m* in order to account for water evaporation (and how to undo such changes). The bottom panel reports the way *Savetable.m* should be set to run the evaporation scenario.

In order to include these variations in APEX, one should define depth as an X or Y variable (e.g., “*d* = −1;” “*x* = 1:0.05:10;”; see Figure 9 for where to type it in *Savetable*) and then introduce a modification in the *apex.m* file (see Figure 9 as well). Modifying the *apex.m* file can be potentially dangerous (in case of a mistake, the whole application might no longer work, or work erroneously). I personally operate in this way: make a new folder (e.g., *C:\Apex_Arid*), and copy the files from the original *C:\Apex* folder into it. The modifications are carried out in the duplicated *apex.m* file, leaving the original one intact. In this case, one has to call APEX from Octave as: “> cd c:\Apex_Arid” instead of “> cd c:\Apex”.

After that, APEX can be run in the customary way, and Figure 8 shows as an example the output results in the case of the ibuprofen phototransformation pathways. In the sample case shown here, the *Savetable* output file and, particularly, the data in the columns *k_OH*, *k_1O2*, *k_3DOM*, and *k_Phot* were used to plot the rate constants of the single photochemical pathways as a function of the depth (and volume) loss.

## 7. Ibuprofen as a Case Study

Ibuprofen is a non-steroidal, anti-inflammatory drug that is often found in natural waters due to incomplete elimination during wastewater treatment [85]. Among other processes, it can undergo photodegradation in surface waters, which contributes to its removal. However, in addition to the attenuation of the parent compound, ibuprofen phototransformation can also produce the toxic intermediate 4-isobutylacetophenone (IBAP) [77]. The use of APEX can help in the assessment of both ibuprofen photodegradation and IBAP photogeneration kinetics and pathways. The photoreaction parameters that describe the transformation of ibuprofen and the generation of IBAP, and that should be inserted in *Savetable*, are reported in Table 2. The absorption spectrum of ibuprofen is provided in the input file *Ibuprofen.csv*.

**Figure 10:**
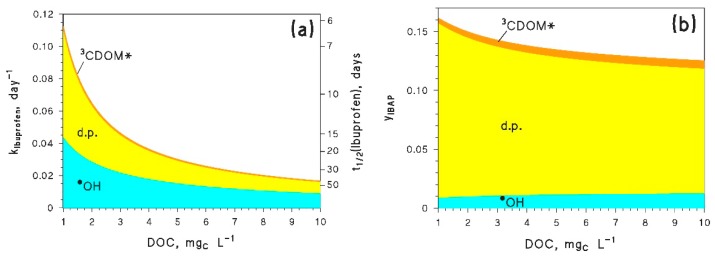
(**a**) Modeled trend of the first-order degradation rate constant of ibuprofen (left Y axis) and of the corresponding half-life time (right Y axis), as a function of the water DOC. (**b**) Modeled DOC trend of the formation yield of IBAP from ibuprofen. In both cases, the weights of the different photoreaction pathways are highlighted in different colors. Other water conditions: 10^−4^ mol·L^−1^ NO_3_^−^, 10^−6^ mol·L^−1^ NO_2_^−^, 10^−3^ mol·L^−1^ HCO_3_^−^, 10^−5^ mol·L^−1^ CO_3_^2−^, and 2 m water depth. Raw data were obtained with *Savetable* and further processed.

Based on the above data, it is possible to assess ibuprofen photodegradation and IBAP photogeneration kinetics for different environmental conditions. For instance, Figure 10 reports the calculation results as a function of the DOC, highlighting the contributions of the different photoreaction pathways. In the case of the IBAP formation yields, the different contributions to the overall yield were calculated as the ratios between the IBAP initial formation rate constant (*kf*) in each pathway and the overall ibuprofen degradation rate constant (*kd*; *dp* = direct photolysis):

(60) $$y_{IBAP}=\frac{kf_{IBAP,dp}+kf_{IBAP,•OH}+kf_{IBAP,1O_{2}}+kf_{IBAP,{}^{3}CDOM*}}{kd_{Ibuprofen}}=\frac{kf_{IBAP,dp}}{kd_{Ibuprofen}}+\frac{kf_{IBAP,•OH}}{kd_{Ibuprofen}}+\frac{kf_{IBAP,1O_{2}}}{kd_{Ibuprofen}}+\frac{kf_{IBAP,{}^{3}CDOM*}}{kd_{Ibuprofen}}$$

The decrease of *k*_Ibuprofen_ with increasing DOC is a typical finding, and is also observed with many other contaminants [27]. In the case of a river, an ibuprofen lifetime of 10 days would correspond to a half-life length of over 860 km if $\bar{\nu }$ = 1 m s^−1^, which in several instances means normal flow conditions [81]. Therefore, a considerable river length would be required to photochemically attenuate ibuprofen. In contrast, severe water scarcity could produce $\bar{\nu }$ = 0.1 m s^−1^ [81], in which case the scenario varies considerably because the half-life length reduces to around 86 km (moreover, river water would be shallower and photoreactions even faster, as explained in Section 6.2).

## 8. Conclusions

This paper shows how to use the APEX software in order to predict the phototransformation kinetics of pollutants, as a function of the environmental conditions. Moreover, because environment-specific adaptations of the original code have been successfully carried out since its first release, hints are here given into how to modify parts of the code to treat back-reduction processes as well as water evaporation. Compared to the original version, the APEX 1.1 code provided here was updated so that it might be easier to make such changes. Additional hints are also given into how to prepare the input data, or how to modify the output data, in order to describe varying pH values, stratification conditions, and river flow. It should be remarked that APEX strongly relies on the input data, and in particular on the photoreactivity parameters of the compounds to be studied. Such parameters include direct photolysis quantum yields and second-order reaction rate constants with photogenerated transient species. These may be known from the literature, or the compound(s) of interest may already be included in the list of 44 molecules for which there are complete data available (they are provided here together with the software, see Supplementary Materials). Otherwise, photoreaction parameters have to be measured in the laboratory or assessed by a QSAR approach. To aid APEX users, experimental protocols to measure photoreaction parameters (e.g., with or without using the competition kinetics methods) have been described in some detail. It has also been described how to approximately assess second-order reaction rate constants on the basis of ^•^OH gas-phase rate constants, and from the one-electron oxidation potential of the given compounds. This work has the ambition of being a key reference for APEX users, and it might hopefully inspire environmental photochemists to make wide use of a potentially powerful tool to predict the photochemical fate of pollutants in surface water bodies.

## Figures and Tables

**Table 1:** Photoreactivity parameters of some reference compounds that can be used in competition kinetics experiments, in order to measure the reaction rate constants with ^•^OH, CO_3_^•−^, ^1^O_2_, and ^3^CBBP* (the latter as ^3^CDOM* proxy) [59,65,68]. The potential of the given compound to undergo direct photolysis for irradiation at λ > 290 nm (which causes some additional complications in data treatment) is also specified. n/a = not available.

	$k_{S+•OH},$ M^−1^ s^−1^	$k_{S+CO_{3}^{•-}},$ M^−1^ s^−1^	$k_{S+1^{}O_{2}},$ M^−1^ s^−1^	$k_{S+{}^{3}CBBP*},$ M^−1^ s^−1^	Direct Photolysis
Acesulfame K	5.9 × 10^9^	n/a	2.8 × 10^4^	n/a	No
Acetaminophen	1.9 × 10^9^	3.8 × 10^8^	3.7 × 10^7^	1.6 × 10^9^	Yes (UVB & UVA)
Atrazine	2.7 × 10^9^	4 × 10^6^	n/a	7.2 × 10^8^	Yes (UVB & UVA)
Tyrosine	1.3 × 10^10^	4.5 × 10^7^	8 × 10^6^	n/a	Yes (UVB & UVA)

**Table 2:** Photoreactivity parameters of ibuprofen degradation and 4-isobutylacetophenone (IBAP) formation, relevant to the different photoreaction pathways. Φ = direct photolysis quantum yield, *k* = second-order reaction rate constant, and *y* = formation yield of the intermediate from the parent compound.

	Direct Photolysis	^•^OH	^1^O_2_	^3^CDOM*
Ibuprofen [19]	Φ = 0.33	*k* = 1.0 × 10^10^ M^−1^ s^−1^	*k* = 6 × 10^4^ M^−1^ s^−1^	*k* = 4.5 × 10^7^ M^−1^ s^−1^
IBAP [77]	*y* = 0.25	*y* = 0.023	*y* ~ 0	*y* = 0.31

## Supplementary Materials

The following are available online at https://www.mdpi.com/1420-3049/25/1/9/s1, APEX user’s guide: *readme.pdf*; the APEX software, version 1.1: *APEX1_1.zip* (compressed file); Octave, version 3.2.4: *3.2.4_gcc-4.4.0.zip* (compressed file).
