# Supplementary material for: A Critical View of the Application of the APEX Software (Aqueous Photochemistry of Environmentally-Occurring Xenobiotics) to Predict Photoreaction Kinetics in Surface Freshwaters
Source: Molecules. 2019 Dec 18;25(1):9. doi: 10.3390/molecules25010009 (PMC7017383; doi:10.3390/molecules25010009)
Supplement: Supplementary file 1 [file molecules-25-00009-s001.zip › Readme.pdf]

USER'S GUIDE

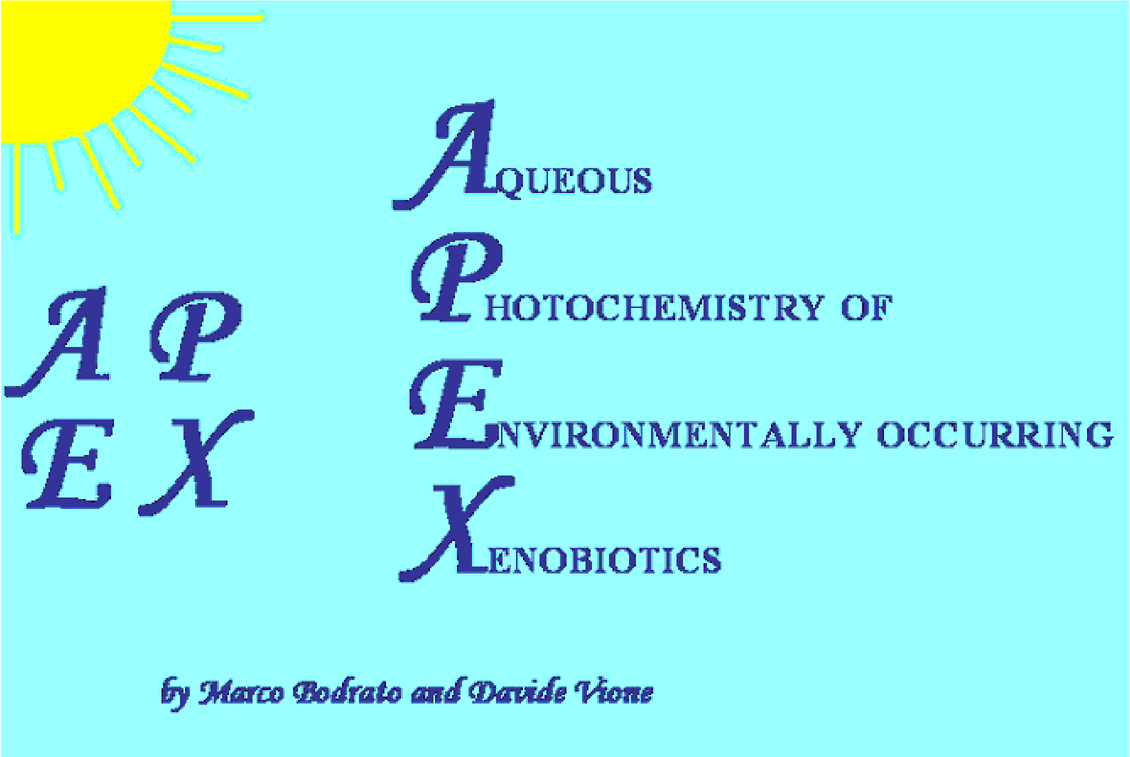

APEX Version 1.1

DISCLAIMER

As freely available software, ***Apex*** comes with absolutely no warranty. Any liability is excluded in relation to the use of ***Apex***, or to decisions made based on the outputs of ***Apex*** calculations. In particular (but without limitation), the authors shall not be liable to anybody for any direct loss of profits or other economic loss, or for any indirect, consequential, special or incidental damages, losses, expenses or claims arising out of the use (or inability to use) the ***Apex*** application.

HOW TO CITE APEX

If you are going to present results obtained with ***Apex*** in a scientific publication or in a congress communication, you should give full acknowledgements to the authors of this software. There are two possible options to cite ***Apex***: the first is to cite the 2014 paper:

M. Bodrato, D. Vione. APEX (Aqueous Photochemistry of Environmentally-occurring Xenobiotics): A Free Software Tool to Predict the Kinetics of Photochemical Processes in Surface Waters. *Environ. Sci.: Processes Impacts* **2014**, *16*, 732-740.

The second is to cite the article, of which this file is part of the Supplementary Material.

⇨ It is highly recommended to have this guide open for reference while browsing through APEX on your computer, as least the first times you are using the software.

Table of contents

1. Introduction **4**

2. How to install APEX on your computer **5**

2.1. Start with Octave ***5***
2.2. Unzip APEX ***7***
2.3. Test APEX ***9***
Table 1. Photoreaction parameters of 44 compounds 11

3. The photochemical model for surface waters **14**

3.1. Surface-water absorption spectrum ***14***

3.2. Reaction with $^{\bullet}OH$ ***14***

3.3. Direct photolysis ***18***

3.4. Reaction with $CO_3^{-\bullet}$ ***19***

3.5. Reaction with $^1O_2$ ***20***

3.6. Reaction with $^3CDOM^*$ ***22***

3.7. Formation of intermediates ***23***

3.8. The meaning of water depth in the model ***23***

3.9. Main approximations of the model ***24***

3.10. How to derive [HCO_3^-] and [CO_3^{2-}] from alkalinity and pH ***25***

4. The Apex software **27**

5. Input files (.csv) **29**

6. Plotgraph and Savetable **32**

6.1. Plotgraph (file plotgraph.m) ***32***

6.1.1. Range input *32*

6.1.2. Input file prefix *33*

6.1.3. Data input *33*

6.1.4. Output selection *35*

6.1.5. Quantum yields ($^{\bullet}OH$, $CO_3^{-\bullet}$, $^1O_2$ and $^3CDOM^*$) *39*

6.2. Savetable ***40***

6.2.1. How to define a variable as constant *42*

6.3. Correction for the solar zenith angle ***43***

7. Running Plotgraph and Savetable under Octave **45**

7.1. Plotgraph ***45***

7.2. Savetable ***46***

7.3. Calculation of model errors ***48***

7.4. Seasonal corrections at mid latitude ***49***

Appendix (Model errors) **52**

1. Introduction

APEX (Aqueous Photochemistry of Environmentally-occurring Xenobiotics) is a code to predict the photochemical fate of aqueous pollutants toward the main photochemical processes that take place in well-mixed surface waters (direct photolysis and reaction with $^{\bullet}OH$, $CO_3^{-\bullet}$, $^1O_2$ and $^3CDOM^*$), as a function of environmental features and of intrinsic substrate reactivity.

Environmental features that can be defined as input data are water depth and chemical composition. In particular, chemical parameters that significantly affect photochemistry are nitrate, nitrite, carbonate, bicarbonate and dissolved organic carbon (DOC, sometimes also named NPOC: non-purgeable organic carbon).

Substrate-dependent features are connected with photochemical reactivity (photolysis quantum yield and reaction rate constants), and to intermediate formation yields *via* the relevant processes.

The fate of pollutants is described by output variables such as first-order transformation rate constants, half-life times and rate constants of intermediate formation. The model also returns steady-state [$^{\bullet}OH$], [$CO_3^{-\bullet}$], [$^1O_2$] and [$^3CDOM^*$]. An overall scheme of APEX is provided below.

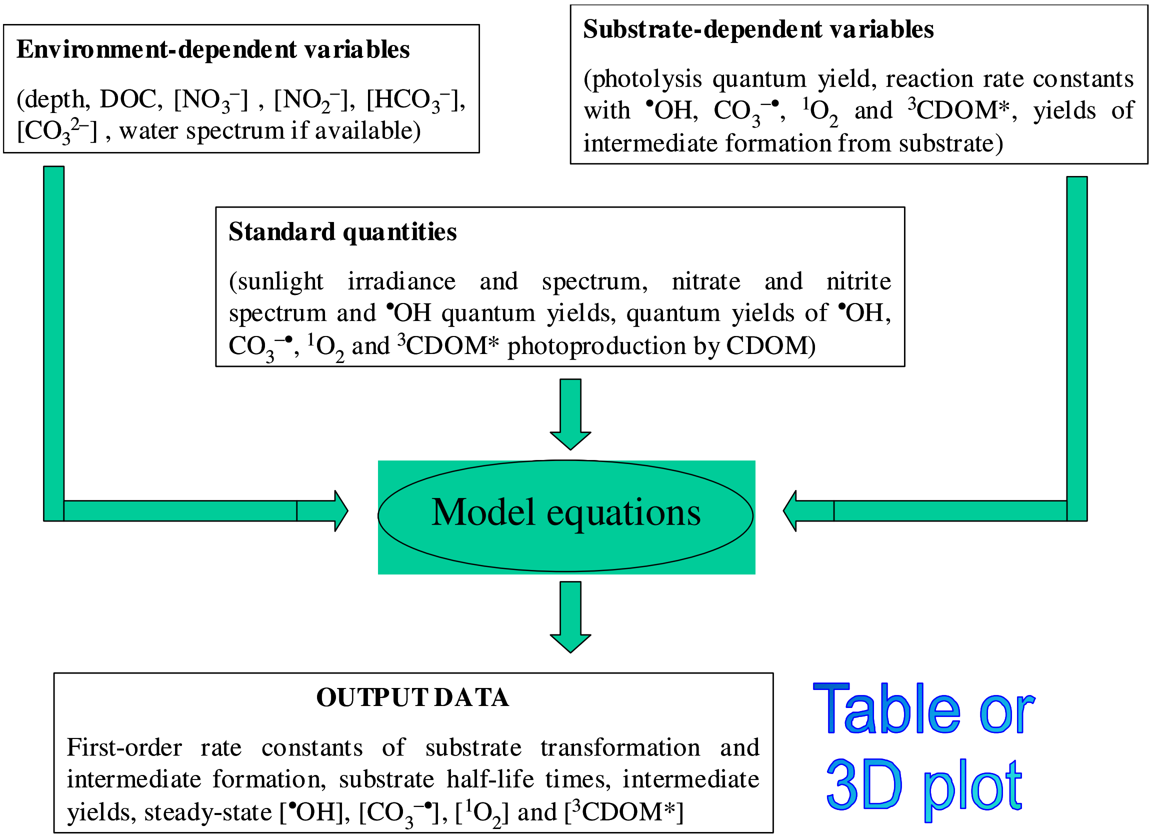

The model equations are described in section 3. Section 2 explains how to install APEX.

2. How to install APEX on your computer

2.1. Start with Octave

First of all, it should be considered that APEX is not self-standing software but requires Octave to function. Therefore, the first thing is to install Octave on your computer. While initially designed to work with Linux systems, many Octave versions for Windows are available (https://wiki.octave.org/Octave_for_Microsoft_Windows) (Note that these web links may not work in this .pdf file. Please copy and paste them in your Internet browser).
I personally use the 3.2.4 version of Octave, and APEX has been extensively tested on it. Therefore, if Octave 3.2.4 works on your computer, APEX will most likely work as well. Because it is not so easy to find Octave 3.2.4 for Windows, it is provided inside the *SM.zip* file of the Supplementary Material. This file contains the following items:

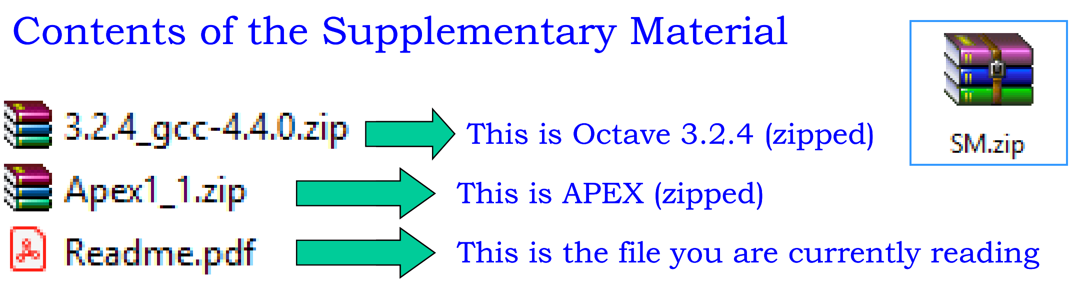

My advice is to try Octave 3.2.4 first. If it does not work (*e.g.*, too old), then go to the website https://wiki.octave.org/Octave_for_Microsoft_Windows to get a newer version.
The *SM.zip* file can be unzipped anywhere to obtain the three files it contains (if you can read these lines, you have already unzipped or at least opened the *SM.zip* file). In most cases you'll have suitable unzipping software on your computer. In case of problems, you can download WinRAR here (https://www.win-rar.com).

The file *3.2.4_gcc-4.4.0.zip* can be unzipped anywhere. One possibility is to unzip it inside the *Program* folder, but the exact position does not matter. The important thing is that *3.2.4_gcc-4.4.0.zip* contains all the sub-folders that are needed for Octave to work, thus there is no need to look for *Setup.exe* (which does not exist, by the way, somewhat of an homage to the Linux, not Windows, origin of the software).

Unzipping of *3.2.4_gcc-4.4.0.zip* is relatively easy because all the files are contained within one folder (when opened, it looks like this: 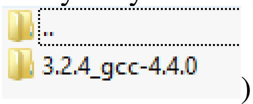

). Therefore, the unzipped files will not mess up the unzipping place with plenty of scattered items.

The *3.2.4_gcc-4.4.0* folder contains several sub-folders, and you should look into *bin* to find the proper executable (*octave.3.2.4.exe*, which starts the software). By far the easiest option is to create a Desktop shortcut. Moreover, you can make it look better by linking the shortcut with the *octave.ico* icon file (see below).

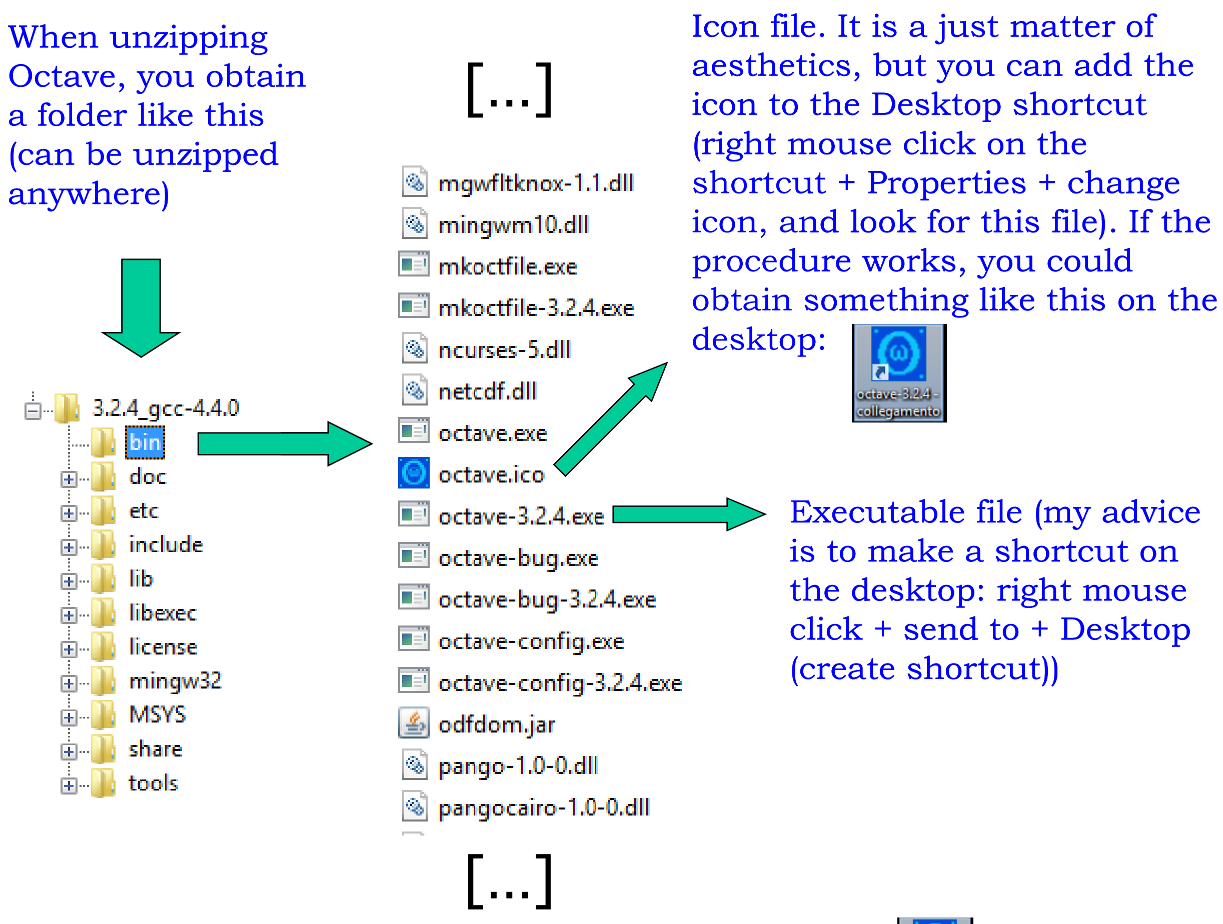

If everything works well, after double-clicking on the desktop icon 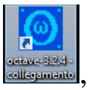, you should obtain the following result (except for the operating system language on the Window frame, in Italian on my computer. Note that more recent versions of Octave look quite different):

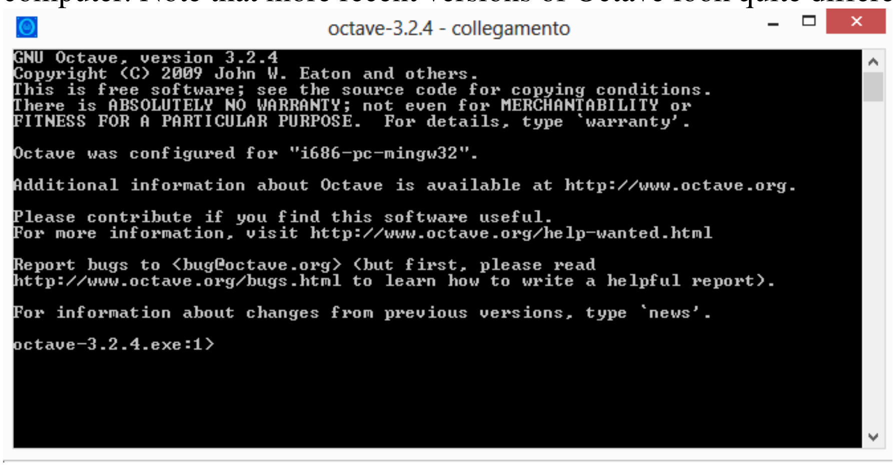

If this is OK, it is now time to install APEX.

2.2 Unzip APEX

The *APEX1_1.zip* file again contains everything in a single folder (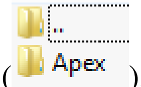

), in order not to mess things while unzipping. However, be careful not to unzip it as *\Apex\Apex* (an *Apex* folder within another *Apex* folder: nothing dramatic with that, but it will be more time consuming to call APEX within Octave).

My advice is to unzip APEX in a comfortable place where to recall it from Octave. I usually place it directly under *C:*, and this is the way the folders may look like:

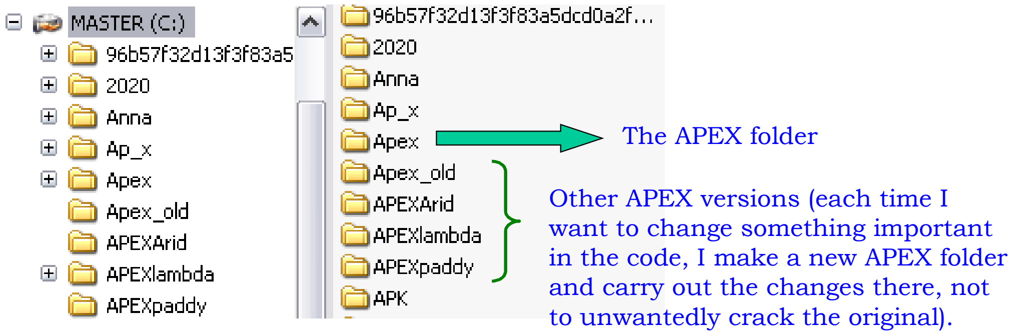

The contents of the Apex folder are shown below here:

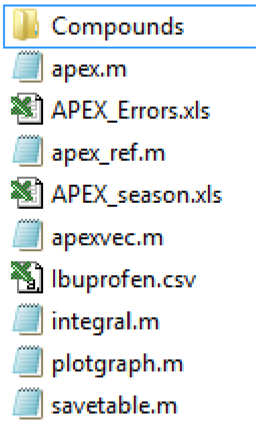

The key files are the *.m* ones. They are actually ASCII files, and my advice is to have them opened with *Notepad* or similar software (the file icons look like this, only after linking the *.m* files with *Notepad* as default software to open them). Avoid word processors, because they tend to add unwanted lines when saving the files, and these additional lines may prevent APEX from working.

That said, here are the file names and their characteristics:

apex.m — This is the calculation engine that carries out several procedures including numerical integration. In most cases there is no need to modify it (which might be dangerous). See the main article for the few cases where modifications may be needed to enable new operations.

apex_ref.m — This is a backup file for *apex.m*, just in case the original file may be irreversibly damaged (a thing of the kind: I made some mistaken change, and I do not know any longer how to restore the original version…it may happen). If in need, delete the damaged *apex.m*, and save a new *apex.m* file from *apex_ref.m* ("Save as…" option, or "copy" and "paste" from one file to the other).

apexvec.m — Service file, do not modify.

integral.m — Integration function, works with the trapeze method. No need to modify, unless you want to use a more performing method (note, however, that this is certainly not the main source of errors/approximations).

plotgraph.m — You'll need to routinely open and modify this file, to introduce part of the input data (vide infra on how to use it).

savetable.m — Same as plotgraph, just the previous contains the instructions for plotting 3D graphs, while this one produces the output data in tabular form.

The two Excel® files, *APEX_Errors.xls* and *APEX_season.xls*, are the tools to compute, respectively, the model uncertainties and the trend of the transformation kinetics in the different months of the year. Basically, they work by copying and pasting a whole line from the output .csv file produced by *savetable.m*, carrying out additional calculations (*vide infra* for more details).

The .csv file of the kind "*molecule.csv*" (here it is *Ibuprofen.csv*) contains the spectral input data, including the absorption spectrum of the relevant molecule (ibuprofen in this case). Several other such files are provided in the *Compounds* folder, for the molecules that have already been studied and fully characterised for their photochemical reactivity.

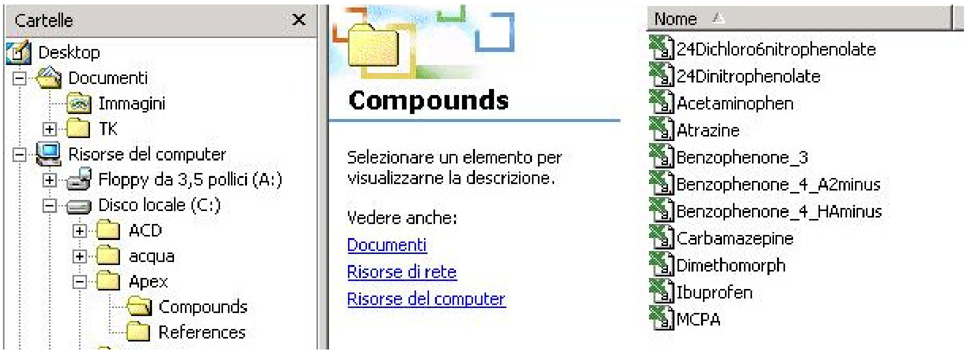

These files are in a dedicated folder to avoid messing things up, but they cannot be used there. In order to use these input files, you should copy them to the *C:\Apex* folder. For instance, in case you want to model the photodegradation of Acetaminophen, the *Acetaminophen.csv* file should appear here:

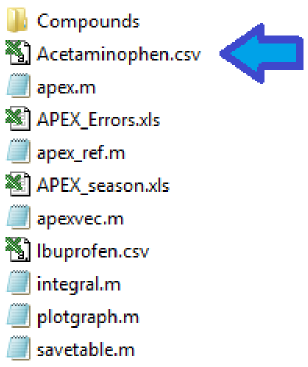

Therefore, you have to move "*Acetaminophen.csv*" from the *C:\Apex\Compounds* folder to *C:\Apex*, in order to use it. (Note: the *.csv* file icons appear like this only after choosing Excel® as default to open them. In so doing, pay attention to the column data separation options: the correct one is "comma separation" or equivalent).

2.3. Test APEX

To make a first test that everything works correctly, open the Octave shortcut (double click on 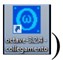), wait for the window below to open, and then digit "*cd c:\Apex* ↵" (↵ = return) and "*plotgraph()* ↵", as shown below (the arrow is just intended to highlight the items to be typed):

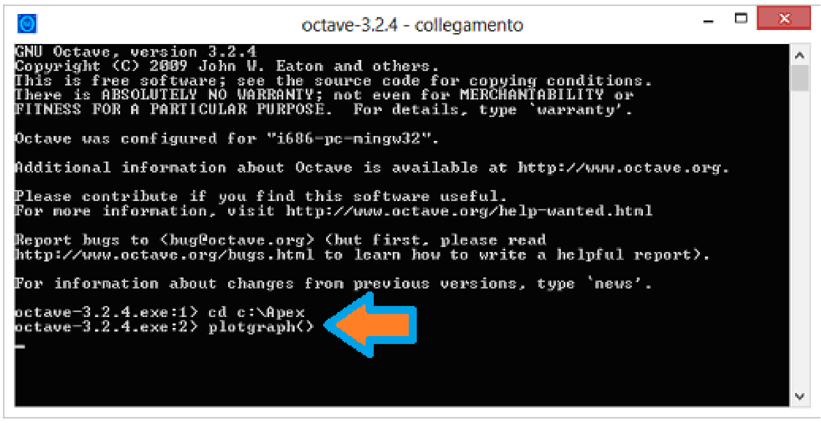

If everything works well, after some time (to have a comparative standard, the plot took around 8 s on a computer equipped with a i7-3632QM CPU @ 2.20 GHz; at the other extreme, 55 s on a Celeron CPU @ 333 MHz), you should get a result like this:

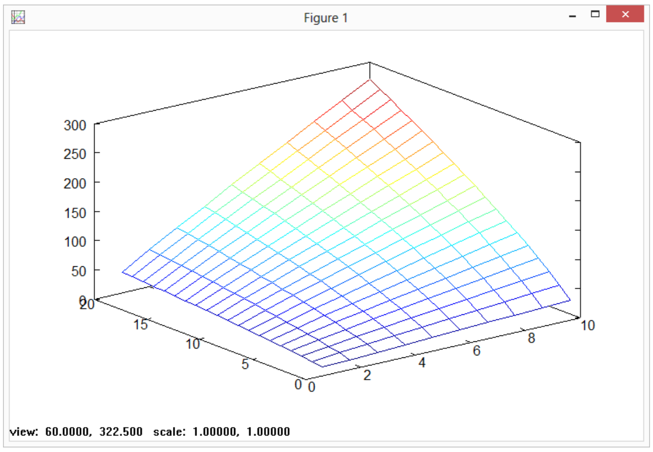

The 3D plot represents the half-life time of ibuprofen under mid-latitude, fair-weather summertime conditions, as a function of water depth varying between 1 and 10 m, and the dissolved organic carbon (DOC) between 1 and 20 mg_C L^{-1}. The axes are unfortunately mute, thus one needs to add titles later on with a graphical program (e.g., Paint® or equivalent). It is also better to define different scales on the X- and Y-axes, like in this case, so as to easily tell what is what. The graphical resolution in this default instance is kept very low (a 10×20 grid is definitely not the utmost aesthetics) to let the calculation work reasonably on most computers. With the same molecule and conditions, but by setting a denser grid that results in much longer computer time you will get a nicer plot (it took about 12 minutes by using the same i7-3632QM CPU as above, not even tried with the other: allow for scaling according to the performance differences you noticed in the previous test):

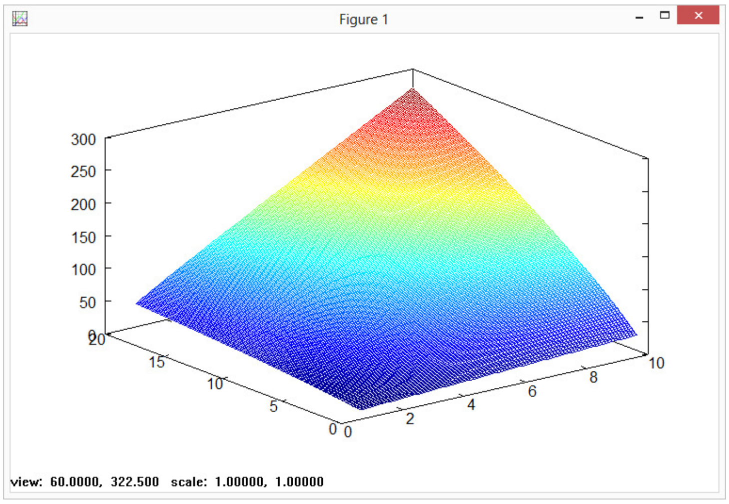

Octave can be closed after use by typing " *exit* ↵ ".

That shown here was just the preliminary test, to check that everything works properly (for instance, note that I did not yet tell you how to obtain the last plot). The theoretical basis of the APEX software and the instructions on how to use it are provided in the next pages.

Before dealing with the software functioning, it may be useful to consider that it strongly relies upon the input data. In particular, the direct photolysis quantum yield of a given molecule and the second-order reaction rate constants with photogenerated transient species (hydroxyl radical $^{\bullet}OH$, carbonate radical $CO_3^{\bullet-}$, singlet oxygen $^1O_2$, and chromophoric dissolved organic matter triplet states $^3CDOM^*$), if not known from the literature, should be measured experimentally. The experimental procedures and the QSAR approaches on how to do this are described in the main manuscript. However, you might be lucky enough that the compound of interest belongs to the list of 44 molecules for which the input files are already available, and the photoreactivity parameters are known. For these molecules, the input files are those contained in the *Compounds* folder (the files should be moved for use as explained above), while the table below lists filenames, quantum yields and second-order reaction rate constants with $^{\bullet}OH$, $CO_3^{\bullet-}$, $^1O_2$ and $^3CDOM^*$.

Table 1. List of photoreactivity parameters for the compounds with input *.csv* files. When relevant, $DOC_{1/2}$ is expressed in [mg_C L^{-1}] units.

Compound S	Filename	$\Phi_{d.p.}$, unitless	$k_{S+^{\bullet}OH}$, M^{-1} s^{-1}	$k_{S+CO_3^{\bullet-}}$, M^{-1} s^{-1}	$k_{S+^1O_2}$, M^{-1} s^{-1}	$k_{S+^3CDOM^*}$, M^{-1} s^{-1}	Ref.
Pesticides							
Atrazine	Atrazine.csv	1.6×10^{-2}	2.7×10^{9}	4×10^{6}	Negligible	7.15×10^{8}	1
Bentazone	Bentazone.csv	4.4×10^{-4}	5.7×10^{9}	2.5×10^{7}	3.1×10^{7}	9.7×10^{8}	2
Chlorotoluron	Chlortoluron.csv	3×10^{-2}	6.9×10^{9}	1.7×10^{7}	Negligible	2.7×10^{9}	3
Dimethomorph	Dimethomorph.csv	2.6×10^{-5}	2.6×10^{10}	Negligible	8.5×10^{5}	1.6×10^{9}	4
Diuron	Diuron.csv	1.25×10^{-2}	9.45×10^{9}	8.3×10^{6}	Negligible	5.2×10^{8}	3
Fenuron	Fenuron.csv	6×10^{-3}	7×10^{9}	6.0×10^{6}	Negligible	2.0×10^{9}	3
Imazethapyr	Imazethapyr.csv	0.023	7.8×10^{9}	n/a	n/a	10^{7}	5
Isoproturon	Isoproturon.csv	2×10^{-3}	7.9×10^{9}	3×10^{7}	Negligible	3.2×10^{9}	3
Metoxuron	Metoxuron.csv	2.0×10^{-2}	5.8×10^{9}	1.1×10^{8}	Negligible	3.1×10^{9}	3
Propanil	Propanil.csv	0.16	7.0×10^{9}	1.4×10^{7}	7.1×10^{4}	1×10^{7}	6
Pharmaceuticals and Personal Care Products (PPCPs)							
Acesulfame K	AcesulfameK.csv	Negligible(*)	5.9×10^{9}	Negligible	2.8×10^{4}	Negligible	7
Acetaminophen	Acetaminophen.csv	4.6×10^{-2}	1.9×10^{9}	3.8×10^{8}	3.7×10^{7}	1.6×10^{9}	8,9
Amoxicillin	Amoxicillin.csv	6×10^{-3}	6.9×10^{9}	n/a	Negligible	2×10^{9}	10
Carbamazepine	Carbamazepine.csv	7.8×10^{-4}	1.8×10^{10}	Negligible	1.9×10^{5}	7.5×10^{8}	11
Cefalexin	Cefalexin.csv	9.1×10^{-2}	7.1×10^{9}	n/a	Negligible	n/a	10
Cefapirin	Cefapirin.csv	7×10^{-3}	n/a	n/a	Negligible	n/a	10
Cefazolin	Cefazolin.csv	6.0×10^{-2}	6.5×10^{9}	n/a	Negligible	10^{8}	10
Cefotaxime	Cefotaxime.csv	1×10^{-3}	8.1×10^{9}	5×10^{7}	Negligible	n/a	10
Cefradine	Cefradine.csv	7.6×10^{-2}	1.1×10^{10}	n/a	Negligible	n/a	10

**Compound S**	**Filename**	**$\Phi_{d.p.}$, unitless**	$k_{S+^{\bullet}OH}$, **M^{-1} s^{-1}**	$k_{S+CO_3^{\bullet-}}$, **M^{-1} s^{-1}**	$k_{S+^1O_2}$, **M^{-1} s^{-1}**	$k_{S+^3CDOM^*}$, **M^{-1} s^{-1}**	**Ref.**
Diclofenac	Diclofenac.csv	9.4×10^{-2}	9.3×10^{9}	Negligible	1.3×10^{7}	6.4×10^{8}	12
Gemfibrozil	Gemfibrozil.csv	5.2×10^{-2}	8.6×10^{9}	n/a	2.6×10^{6}	Negligible	13
Ibuprofen	Ibuprofen.csv	0.33	1.0×10^{10}	Negligible	6.0×10^{4}	4.5×10^{7}	14,9
Naproxen	Naproxen.csv	0.005-0.01	8×10^{9}	Negligible	1.1×10^{5}	7.5×10^{8}	12
Sertraline	Sertraline.csv	0.95	2×10^{10}	2×10^{8}	1.3×10^{6}	7×10^{9}	15
Sulfadiazine	Sulfadiazine_HSDZ.csv	4×10^{-4}	3.7×10^{9}	n/a	8.9×10^{6}	4.9×10^{9} ($DOC_{1/2}$ = 17)	16
	Sulfadiazine_SDZ-.csv	1.2×10^{-3}	3.7×10^{9}	n/a	8.9×10^{6}	2.9×10^{9} ($DOC_{1/2}$ = 3.2)	16
Triclosan	Triclosan_HTric.csv	0.3	5.4×10^{9}	n/a	3×10^{6}	3.1×10^{9}	17
	Triclosan_Tric-.csv	0.3	1×10^{10}	n/a	1.1×10^{8}	4.3×10^{9}	17
UV Filters							
Benzophenone-3	Benzophenone-3.csv	3.1×10^{-5}	2.0×10^{10}	Negligible	2.0×10^{5}	1.1×10^{9}	18
Benzophenone-4	Benzophenone-4_HBP4. csv	3.2×10^{-5}	1.9×10^{10}	Negligible	Negligible	Negligible	19
	Benzophenone-4_BP4-.csv	7.0×10^{-5}	8.5×10^{9}	Negligible	Negligible	Negligible	19
Ethylhexylmethoxy cinnamate	EHMC.csv	3.8×10^{-2} (**)	$\leq 2\times10^{10}$	n/a	3.7×10^{7}	$\leq 5\times10^{9}$	20
2-Ethylhexyl 4-(dimethylamino)benzoate	ODPABA.csv	3.8×10^{-2} (**)	$\leq 2\times10^{10}$	n/a	1.3×10^{8}	$\leq 5\times10^{9}$	21
Ionic liquids							
1-Butyl-4-methylpyridinium tetrafluoroborate	BMPOTFB.csv	0.45	2.8×10^{8}	4.85×10^{6}	1.5×10^{6}	2×10^{9}	22
1-(3-Cyanopropyl)pyridinium chloride	CPPC.csv	0.78	1.55×10^{7}	1.5×10^{6}	1.3×10^{6}	2.6×10^{7}	22
1-Ethyl-3-methylimidazolium hydrogensulfate	EMIM.csv	0.12	2.1×10^{10}	Negligible	8.5×10^{5}	2.7×10^{8}	23
1-Ethylpyridinium tetrafluoroborate	EPTFB.csv	4.8×10^{-2}	3.3×10^{7}	7.0×10^{5}	1.4×10^{6}	3.7×10^{7}	22

**Compound S**	**Filename**	**$\Phi_{d.p.}$, unitless**	$k_{S+^{\bullet}OH}$, **M^{-1} s^{-1}**	$k_{S+CO_3^{\bullet-}}$, **M^{-1} s^{-1}**	$k_{S+^1O_2}$, **M^{-1} s^{-1}**	$k_{S+^3CDOM^*}$, **M^{-1} s^{-1}**	**Ref.**
Transformation intermediates and miscellaneous compounds							
2,4-Dichloro-6-nitrophenolate	2,4-Dichloro-6-nitrophenolate.csv	4.5×10^{-6}	2.8×10^{9}	Negligible	3.7×10^{9}	1.4×10^{8}	24
2,4-Dinitrophenolate	2,4-Dinitrophenolate.csv	3.5×10^{-5}	2.3×10^{9}	n/a	Negligible	Negligible	25
3,4-Dichloroaniline	3,4-Dichloroaniline.csv	4×10^{-2}	1.3×10^{10}	4.8×10^{8}	n/a	1.1×10^{9}	26
1H-Benzotriazole	Benzotriazole_HBZT.csv	5.4×10^{-2}	7.6×10^{9}	Negligible	Negligible	1.9×10^{7}	27
	Benzotriazole_BZT-.csv	9.4×10^{-3}	9.0×10^{9}	Negligible	Negligible	1.5×10^{9}	27
Glutathione	Glutathione.csv	Negligible (*)	3.5×10^{9}	5.3×10^{6}	2.4×10^{6}	6.7×10^{8}	28
Nitrobenzene	Nitrobenzene.csv	5.7×10^{-3}	3.9×10^{9}	Negligible	Negligible	1.1×10^{8}	29

(*) This compound does not absorb sunlight, thus it does not undergo direct photolysis under environmental conditions.

(**) The direct photolysis would be by far the main phototransformation pathway, irrespective of the values of the reaction rate constants with $^{\bullet}OH$ and $^3CDOM^*$.

References

1	*Water Res.* **2013**, *47*, 6211-6222	11	*Environ. Sci. Technol.* **2012**, *46*, 8164-8173	21	*Water Res.* **2016**, *88*, 235-244
2	*Chemosphere*, under revision	12	*Water Res.* **2016**, *105*, 383-394	22	*Environ. Sci. Technol.* **2015**, *49*, 10951-10958
3	*Chemosphere* **2015**, *119*, 601-607	13	*Chemosphere* **2017**, *170*, 124-133	23	*Water Res.* **2017**, *122*, 194-206
4	*Sci. Total Environ.* **2014**, *500-501*, 351-360	14	*Water Res.* **2011**, *45*, 6725-6736	24	*Environ. Sci. Technol.* **2011**, *45*, 209-214
5	*Sci. Total Environ.* **2018**, *644*, 1391-1398	15	*Environ. Pollut,* in press.	25	*Chemosphere* **2010**, *80*, 759-763
6	*Environ. Sci. Technol.* **2017**, *51*, 2695-2704	16	*Water Res.* **2018**, *128*, 38-48	26	*Environ. Sci. Technol.* **2018**, *52*, 6334-
7	*Chemosphere* **2017**, *186*, 185-192	17	*Water Res.* **2015**, *72*, 271-280	27	*Sci. Total Environ.* **2016**, *566-567*, 712-721
8	*Water Res.* **2014**, *53*, 235-248	18	*Sci. Tot. Environ.* **2013**, *463-464*, 243-251	28	*Chemosphere* **2018**, *209*, 401-410
9	*Chemosphere* **2019**, *237*, article 124476	19	*Wat. Res.* **2013**, *47*, 5943-5953	29	*Chemosphere* **2016**, *145*, 277-283
10	*Chemosphere* **2015**, *134*, 452-458	20	*Sci. Total Environ.* **2015**, *537*, 58-68		

3. The photochemical model for surface waters

The model describes the transformation kinetics of a substrate, a generic pollutant P, as a function of water chemistry and substrate reactivity, *via* the main photochemical reaction pathways (direct photolysis and reaction with $^{\bullet}OH$, $CO_3^{-\bullet}$, $^1O_2$ and $^3CDOM^*$). It also calculates the steady-state concentrations of photogenerated transient species. The model may use actual data of water absorption spectrum or, in their absence, it can approximate the spectrum from the dissolved organic carbon values. The different aspects of the model are now described in greater detail.

3.1. Surface-water absorption spectrum

It is possible to find a reasonable correlation between the absorption spectrum of surface waters and their content of dissolved organic matter, expressed as NPOC (Non-Purgeable Organic Carbon). The following equation holds for the water spectrum, referred to an optical path length of 1 cm:[1]

$$\mathrm{A}_1(\lambda) = (0.45 \pm 0.04) \cdot NPOC \cdot \mathrm{e}^{-(0.015 \pm 0.002) \cdot \lambda} \qquad (1)$$

As an obvious alternative, $A_1(\lambda)$ can be spectrophotometrically determined on a real water sample.

3.2. Reaction with $^{\bullet}OH$ [1]

In natural surface waters under sunlight illumination, the main $^{\bullet}OH$ sources are (in order of average importance) Chromophoric Dissolved Organic Matter (CDOM), nitrite, and nitrate. All these species produce $^{\bullet}OH$ upon absorption of sunlight. The calculation of the photon fluxes absorbed by CDOM, nitrate and nitrite requires taking into account the mutual competition for sunlight irradiance. Actually, CDOM is the main radiation absorber in the 300-500 nm region, where also nitrite and nitrate absorb radiation. At a given wavelength λ, the ratio of the photon flux densities absorbed by two different species is equal to the ratio of the respective absorbances. The same is also true for the ratio of the photon flux density absorbed by species to the total photon flux density absorbed by the solution, $p_a^{tot}(\lambda)$.[2] Accordingly, the following equations hold for the different $^{\bullet}OH$ sources (note that $A_1(\lambda)$ is the specific absorbance of the surface water layer over a 1 cm optical path length, in units of cm^{-1}; *d*, in metres, is the optical path length of sunlight in water, which is proportional to the water depth (see section 5.3); $A_{tot}(\lambda)$ is the total absorbance of the water column, and $p°(\lambda)$ is the spectrum of sunlight, expressed as incident photon flux density):

$$A_{tot}(\lambda) = 100\, A_1(\lambda) \cdot d \qquad (2)$$

[1] D. Vione, R. Das, F. Rubertelli, V. Maurino, C. Minero, S. Barbati, S. Chiron, Modelling the occurrence and reactivity of hydroxyl radicals in surface waters: Implications for the fate of selected pesticides. Intern. J. Environ. Anal. Chem. 90 (2010) 258-273.

[2] S. E. Braslavsky, Glossary of terms used in photochemistry, 3rd edition. Pure Appl. Chem. 79 (2007) 293-465.

$$A_{NO3-}(\lambda) = 100\ \varepsilon_{NO3-}(\lambda) \cdot d \cdot [NO_3^-] \qquad (3)$$

$$A_{NO2-}(\lambda) = 100\ \varepsilon_{NO2-}(\lambda) \cdot d \cdot [NO_2^-] \qquad (4)$$

$$A_{CDOM}(\lambda) = A_{tot}(\lambda) - A_{NO3-}(\lambda) - A_{NO2-}(\lambda) \approx A_{tot}(\lambda) \qquad (5)$$

$$p_a^{tot}(\lambda) = p°(\lambda) \cdot (1 - 10^{-A_{tot}(\lambda)}) \qquad (6)$$

$$p_a^{CDOM}(\lambda) = p_a^{tot}(\lambda) \cdot A_{CDOM}(\lambda) \cdot [A_{tot}(\lambda)]^{-1} \approx p_a^{tot}(\lambda) \qquad (7)$$

$$p_a^{NO2-}(\lambda) = p_a^{tot}(\lambda) \cdot A_{NO2-}(\lambda) \cdot [A_{tot}(\lambda)]^{-1} \qquad (8)$$

$$p_a^{NO3-}(\lambda) = p_a^{tot}(\lambda) \cdot A_{NO3-}(\lambda) \cdot [A_{tot}(\lambda)]^{-1} \qquad (9)$$

An important issue is that $p°(\lambda)$ is usually reported in units of Einstein cm^{-2} s^{-1} nm^{-1} (see for instance Figure 1), thus the absorbed photon flux densities are expressed in the same units. To express the formation rates of $^{\bullet}$OH in mol L^{-1} s^{-1}, the absorbed photon fluxes P_a^i should be expressed in Einstein L^{-1} s^{-1}. Integration of $p_a^i(\lambda)$ over wavelength would give units of Einstein cm^{-2} s^{-1} that represent the moles of photons absorbed per unit surface area and unit time.

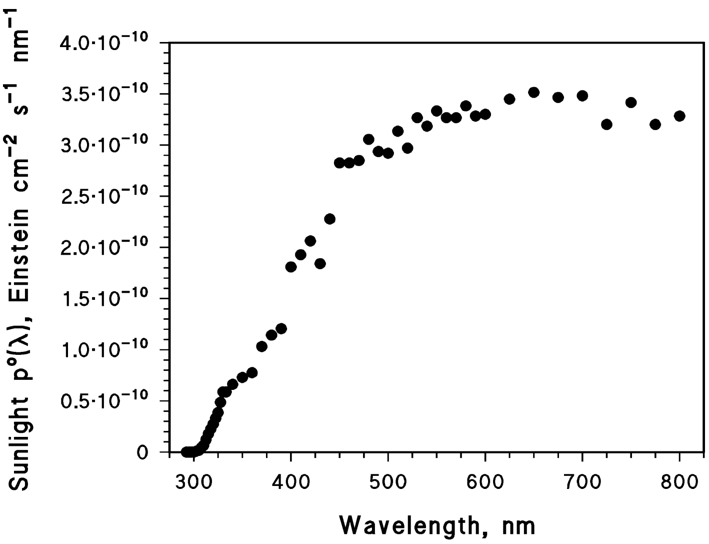

Figure 1. Sunlight spectral photon flux density at the water surface per unit area. The corresponding UV irradiance is 22 W m^{-2}.[3]

By assuming a cylindrical volume of unit surface area (1 cm^2) and height d (expressed in m), the absorbed photon fluxes in Einstein L^{-1} s^{-1} units would be expressed as follows (note that 1 L = 10^3 cm^3 and 1 m = 10^2 cm):

$$P_a^{CDOM} = 10\ d^{-1} \int_{\lambda} p_a^{CDOM}(\lambda)\ d\lambda \qquad (10)$$

$$P_a^{NO2-} = 10\ d^{-1} \int_{\lambda} p_a^{NO2-}(\lambda)\ d\lambda \qquad (11)$$

$$P_a^{NO3-} = 10\ d^{-1} \int_{\lambda} p_a^{NO3-}(\lambda)\ d\lambda \qquad (12)$$

[3] R. Frank, W. Klöpffer, Spectral solar photo irradiance in Central Europe and the adjacent north Sea, Chemosphere 17 (1988) 985-994.

Various studies have yielded useful correlation between the formation rate of $^{\bullet}$OH by the photoactive species and the respective absorbed photon fluxes of sunlight. In particular, it has been found that:[1,4,5]

$$r_{\bullet OH}^{CDOM} = (3.0 \pm 0.4) \cdot 10^{-5} \cdot P_a^{CDOM} \tag{13}$$

$$r_{\bullet OH}^{NO2-} = \int_{\lambda} \Phi_{\bullet OH}^{NO_2^-}(\lambda)\ \ p_a^{NO2-}(\lambda)\, d\lambda \tag{14}$$

$$r_{\bullet OH}^{NO3-} = (4.3 \pm 0.2) \cdot 10^{-2} \cdot \frac{[IC] + 0.0075}{2.25\,[IC] + 0.0075} \cdot P_a^{NO3-} \tag{15}$$

where [IC] = $[H_2CO_3]$ + $[HCO_3^-]$ + $[CO_3^{2-}]$ is the total concentration of inorganic carbon. The wavelength-dependent data of $\Phi_{\bullet OH}^{NO_2^-}(\lambda)$ are reported in Table 2.[5]

Table 2. Values of the quantum yield of $^{\bullet}$OH photoproduction by nitrite, for different wavelengths of environmental significance.

λ, nm	$\Phi_{\bullet OH}^{NO_2^-}(\lambda)$	λ, nm	$\Phi_{\bullet OH}^{NO_2^-}(\lambda)$	λ, nm	$\Phi_{\bullet OH}^{NO_2^-}(\lambda)$
292.5	0.0680	315.0	0.061	350	0.025
295.0	0.0680	317.5	0.058	360	0.025
297.5	0.0680	320.0	0.054	370	0.025
300.0	0.0678	322.5	0.051	380	0.025
302.5	0.0674	325.0	0.047	390	0.025
305.0	0.0668	327.5	0.043	400	0.025
307.5	0.066	330.0	0.038	410	0.025
310.0	0.065	333.3	0.031	420	0.025
312.5	0.063	340.0	0.026	430	0.025

At the present state of knowledge it is reasonable to hypothesise that CDOM, nitrite and nitrate generate $^{\bullet}$OH independently, with no mutual interactions. Therefore, the total formation rate of $^{\bullet}$OH ($r_{\bullet OH}^{tot}$) is the sum of the contributions of the three species:

$$r_{\bullet OH}^{tot} = r_{\bullet OH}^{CDOM} + r_{\bullet OH}^{NO2-} + r_{\bullet OH}^{NO3-} \tag{16}$$

Accordingly, having as input data d, $A_1(\lambda)$, $[NO_3^-]$, $[NO_2^-]$ and $p°(\lambda)$ (the latter referred to a 22 W m^{-2} sunlight UV irradiance, see Figure 1), it is possible to model the expected $r_{\bullet OH}^{tot}$ of the sample. The photogenerated $^{\bullet}$OH radicals could react either with the pollutant P or with the natural scavengers present in surface water (mainly organic matter, bicarbonate, carbonate, bromide and nitrite). The natural scavengers have the following $^{\bullet}$OH scavenging rate constant:

$\Sigma_i\, k_{Si}\, [S_i] = 5\times10^4\ NPOC + 8.5\times10^6\ [HCO_3^-] + 3.9\times10^8\ [CO_3^{2-}] + 1.0\times10^{10}\ [NO_2^-] + 1.1\times10^{10}\ [Br^-]$

[4] D. Vione, S. Khanra, S. Cucu Man, P. R. Maddigapu, R. Das, C. Arsene, R. I. Olariu, V. Maurino, C. Minero, Inhibition vs. enhancement of the nitrate-induced phototransformation of organic substrates by the $^{\bullet}$OH scavengers bicarbonate and carbonate. Wat. Res. 43 (2009) 4718-4728.

[5] J. Mack, J.R. Bolton, Photochemistry of nitrite and nitrate in aqueous solution: a review, J. Photochem. Photobiol. A: Chem. 128 (1999) 1-13.

($\Sigma_i\, k_{Si}\, [S_i]$ has units of s^{-1}; NPOC = non-purgeable organic carbon is a measure of the dissolved organic carbon or DOC, expressed in $mg_C\, L^{-1}$; the other concentration values are in molarity). The steady-state [$^{\bullet}$OH] is the ratio between $r_{^{\bullet}OH}^{tot}$ and the scavenging rate constant. Accordingly, the reaction rate between P and $^{\bullet}$OH ($r_P^{^{\bullet}OH} = k_{P,^{\bullet}OH}\, [P]$ [$^{\bullet}$OH]) can be expressed as follows:

$$r_P^{^{\bullet}OH} = r_{^{\bullet}OH}^{tot} \frac{k_{P,^{\bullet}OH}\,[P]}{k_{P,^{\bullet}OH}\,[P] + \sum_i k_{Si}\,[S_i]} \qquad (17)$$

where $k_{P,^{\bullet}OH}$ is the second-order reaction rate constant between P and $^{\bullet}$OH, and [P] is a molar concentration. Note that, in the vast majority of environmental cases, it would be $k_{P,^{\bullet}OH}\, [P] \ll \Sigma_i\, k_{Si}\, [S_i]$, thus the $k_{P,^{\bullet}OH}\, [P]$ term can be neglected at the denominator of equation (17). The pseudo-first order degradation rate constant of P is $k_P = r_P^{^{\bullet}OH}\, [P]^{-1}$, and the half-life time is $t_P = \ln 2\; k_P^{-1}$. The time t_P is expressed in seconds of continuous irradiation under sunlight, at 22 W m^{-2} UV irradiance (see Figure 1 for the sunlight spectrum). It has been shown that the sunlight energy reaching the ground in a summer sunny day (SSD), such as 15 July at 45°N latitude, corresponds to 10 h = $3.6 \cdot 10^4$ s of continuous irradiation at 22 W m^{-2} UV irradiance.[6] Accordingly the half-life time of P, because of reaction with $^{\bullet}$OH, would be expressed as follows in SSD units:

$$\tau_{P,^{\bullet}OH}^{SSD} = \frac{\ln 2 \sum_i k_{Si}\,[S_i]}{3.6 \cdot 10^4\; r_{^{\bullet}OH}^{tot}\; k_{P,^{\bullet}OH}} = 1.9 \cdot 10^{-5}\, \frac{\sum_i k_{Si}\,[S_i]}{r_{^{\bullet}OH}^{tot}\; k_{P,^{\bullet}OH}} \qquad (18)$$

Note that $1.9 \cdot 10^{-5} = \ln 2\, (3.6 \cdot 10^4)^{-1}$. The steady-state [$^{\bullet}$OH] (molar units) under 22 W m^{-2} UV irradiance would then be:

$$[^{\bullet}OH] = \frac{r_{^{\bullet}OH}^{tot}}{\sum_i k_{Si}\,[S_i]} \qquad (19)$$

Also note that the function ***apex.m*** adopts a slightly different definition of $r_{^{\bullet}OH}^{tot}$, namely the formation rate of $^{\bullet}$OH inside a cylinder of volume $V = 0.1\, d$ (units of mol s^{-1} instead of mol $L^{-1}\, s^{-1}$). For this reason, the term $V = 0.1\, d$ is included in the *apex.m* equation that is equivalent to (18).

[6] C. Minero, S. Chiron, G. Falletti, V. Maurino, E. Pelizzetti, R. Ajassa, M. E. Carlotti, D. Vione, Photochemical processes involving nitrite in surface water samples. Aquat. Sci. 69 (2007) 71-85.

3.3. Direct photolysis [7,8]

The calculation of the photon flux absorbed by P requires taking into account the mutual competition for sunlight irradiance between P and the other water components (mostly Chromophoric Dissolved Organic Matter, CDOM, which is the main sunlight absorber in the spectral region of interest, around 300-500 nm).

Under the Lambert-Beer approximation, at a given wavelength λ, the ratio of the photon flux densities absorbed by two different species is equal to the ratio of the respective absorbances.[2] Accordingly, the photon flux absorbed by P in a water column of optical path length d (expressed in m) can be obtained as follows (note that $A_1(\lambda)$ is the specific absorbance of the surface water sample over a 1 cm optical path length, $A_{tot}(\lambda)$ the total absorbance of the water column, $p°(\lambda)$ the spectrum of sunlight, referred to a UV irradiance of 22 W m^{-2} as per Figure 1, $\varepsilon_P(\lambda)$ the molar absorption coefficient of P, in units of M^{-1} cm^{-1}, and $p_a^P(\lambda)$ the absorbed spectral photon flux density of P; it is also $p_a^P(\lambda) \ll p_a^{tot}(\lambda)$ and $A_P(\lambda) \ll A_{tot}(\lambda)$, in the very vast majority of the environmental cases):

$$A_{tot}(\lambda) = 100\ A_1(\lambda) \cdot d \quad (20)$$

$$A_P(\lambda) = 100\ \varepsilon_P(\lambda) \cdot d \cdot [P] \quad (21)$$

$$p_a^{tot}(\lambda) = p°(\lambda) \cdot (1 - 10^{-A_{tot}(\lambda)}) \quad (22)$$

$$p_a^P(\lambda) = p_a^{tot}(\lambda) \cdot A_P(\lambda) \cdot [A_{tot}(\lambda)]^{-1} \quad (23)$$

To express the rate of P photolysis in mol L^{-1} s^{-1}, the absorbed photon flux P_a^P should be expressed in Einstein L^{-1} s^{-1}. Integration of $p_a^P(\lambda)$ over wavelength gives units of Einstein cm^{-2} s^{-1}, and the absorbed photon flux in Einstein L^{-1} s^{-1} units is expressed as follows (1 L = 10^3 cm^3, 1 m = 10^2 cm):

$$P_a^P = 10\ d^{-1} \int_\lambda p_a^P(\lambda)\ d\lambda \quad (24)$$

The rate of photolysis of P, expressed in mol L^{-1} s^{-1}, is:

$$r_P = 10\ d^{-1} \int_\lambda \Phi_P(\lambda)\ p_a^P(\lambda)\ d\lambda \quad (25)$$

where $\Phi_P(\lambda)$ is the photolysis quantum yield of P in the relevant wavelength interval. If only a single average value for Φ_P is known, Φ_P can be taken out of the integral as a constant. The pseudo-first order degradation rate constant of P is $k_P = r_P\ [P]^{-1}$, which corresponds to a half-life time $t_P = \ln 2\ (k_P)^{-1}$. The time t_P is expressed in seconds of continuous irradiation under sunlight, at 22 W m^{-2} UV irradiance. As per the previous discussion, the half-life time expressed in SSD units would be given by (note that $V = 0.1\ d$):

[7] D. Vione, J. Feitosa-Felizzola, C. Minero, S. Chiron, Phototransformation of selected human-used macrolides in surface water: Kinetics, model predictions and degradation pathways. Wat. Res. 43 (2009) 1959-1967.

[8] D. Vione, M. Minella, C. Minero, V. Maurino, P. Picco, A. Marchetto, G. Tartari, Photodegradation of nitrite in lake waters: role of dissolved organic matter. Environ. Chem. 6 (2009) 407-415.

$\tau^{SSD}{}_P = (3.6{\cdot}10^4)^{-1} \ln 2\, (k_P)^{-1} = 1.9{\cdot}10^{-5}\, [P]\, (r_P)^{-1} =$

$$=1.9{\cdot}10^{-5}\, [P]\, V \left(\int_\lambda \Phi_P(\lambda)\, p_a^P(\lambda)\, d\lambda \right)^{-1} =$$

$$= 1.9{\cdot}10^{-5}\, [P]\, V \left(\int_\lambda \Phi_P(\lambda) \cdot p_a^{tot}(\lambda) \cdot A_P(\lambda) \cdot [A_{tot}(\lambda)]^{-1}\, d\lambda \right)^{-1} =$$

$$\frac{1.9 \cdot 10^{-5}\, V\, [P]}{\int_\lambda \Phi_P(\lambda)\, p°(\lambda)\, (1-10^{-100\, A_1(\lambda)\, d})\, \frac{\varepsilon_P(\lambda)}{A_1(\lambda)}\, d\lambda} \qquad (26)$$

Also note that $1.9{\cdot}10^{-5} = (\ln 2)\, (3.6{\cdot}10^4)^{-1}$.

3.4. Reaction with $CO_3^{-\bullet}$ [9]

The radical $CO_3^{-\bullet}$ is produced upon oxidation of carbonate and bicarbonate by $^{\bullet}OH$, upon carbonate oxidation by $^3CDOM^*$, and possibly also from irradiated Fe(III) oxide colloids and carbonate. However, as far as the latter process is concerned, there is still insufficient knowledge about the Fe speciation in surface waters to enable proper modelling. The main sink of the carbonate radical in surface waters is the reaction with DOM, which is considerably slower than the reaction between DOM and $^{\bullet}OH$.

$^{\bullet}OH + CO_3^{2-} \rightarrow OH^- + CO_3^{-\bullet}$ $[k_{27} = 3.9\times10^8\ M^{-1}\ s^{-1}]$ (27)

$^{\bullet}OH + HCO_3^- \rightarrow H_2O + CO_3^{-\bullet}$ $[k_{28} = 8.5\times10^6\ M^{-1}\ s^{-1}]$ (28)

$^3CDOM^* + CO_3^{2-} \rightarrow CDOM^{-\bullet} + CO_3^{-\bullet}$ $[k_{29} \approx 1\times10^5\ M^{-1}\ s^{-1}]$ (29)

$DOM + CO_3^{-\bullet} \rightarrow DOM^{+\bullet} + CO_3^{2-}$ $[k_{30} \approx 10^2\ (mg\ C)^{-1}\ s^{-1}]$ (30)

The formation rate of $CO_3^{-\bullet}$ in reactions (27, 28) is given by the formation rate of $^{\bullet}OH$, times the fraction of $^{\bullet}OH$ that reacts with carbonate and bicarbonate, as follows:

$$r_{CO_3^{-\bullet}}^{\bullet OH} = r_{^{\bullet}OH}^{tot} \cdot \frac{8.5 \cdot 10^6 \cdot [HCO_3^-] + 3.9 \cdot 10^8 \cdot [CO_3^{2-}]}{5 \cdot 10^4 \cdot NPOC + 1.0 \cdot 10^{10} \cdot [NO_2^-] + 1.1 \cdot 10^{10} \cdot [Br^-] + 8.5 \cdot 10^6 \cdot [HCO_3^-] + 3.9 \cdot 10^8 \cdot [CO_3^{2-}]} \qquad (31)$$

The formation of $CO_3^{-\bullet}$ in reaction (29) is given by:

$$r_{CO_3^{-\bullet}}^{CDOM} = 6.5 \cdot 10^{-3} \cdot [CO_3^{2-}] \cdot P_a^{CDOM} \qquad (32)$$

The total formation rate of $CO_3^{-\bullet}$ is $r_{CO_3^{-\bullet}}^{tot} = r_{CO_3^{-\bullet}}^{\bullet OH} + r_{CO_3^{-\bullet}}^{CDOM}$. The transformation rate of P by $CO_3^{-\bullet}$ is proportional to the fraction of $CO_3^{-\bullet}$ that reacts with P, in competition with reaction (30) between $CO_3^{-\bullet}$ and DOM:

[9] D. Vione, V. Maurino, C. Minero, M. E. Carlotti, S. Chiron, S. Barbati, Modelling the occurrence and reactivity of the carbonate radical in surface freshwater. C. R. Chimie 12 (2009) 865-871.

$$r_P^{CO_3^{-\bullet}} = \frac{r_{CO_3^{-\bullet}}^{tot} \cdot k_{P,CO_3^{-\bullet}} \cdot [P]}{k_{30} \cdot NPOC + k_{P,CO_3^{-\bullet}} \cdot [P]} \quad (33)$$

where $k_{P,CO_3^{-\bullet}}$ is the second-order reaction rate constant between P and $CO_3^{-\bullet}$. In the very vast majority of the environmental cases, it is $k_{P,CO_3^{-\bullet}}$ [P] « k_{30} NPOC.

In a pseudo-first order approximation, the rate constant of P transformation is $k_P = r_P^{CO_3^{-\bullet}}\ [P]^{-1}$ and the half-life time is $t_P = ln\ 2\ k_P^{-1}$. Considering the usual conversion (≈ 10 h) between a constant 22 W m^{-2} sunlight UV irradiance and a SSD unit, the following expression for $\tau_{P,CO_3^{-\bullet}}^{SSD}$ (expressed in SDD) is obtained:

$$\tau_{P,CO_3^{-\bullet}}^{SSD} = 1.9 \cdot 10^{-5} \cdot \left(\frac{k_{30} \cdot NPOC}{r_{CO_3^{-\bullet}}^{tot} \cdot k_{P,CO_3^{-\bullet}}} \right) \quad (34)$$

Note that $1.9 \cdot 10^{-5}$ = ln 2 $(3.6 \cdot 10^4)^{-1}$. The steady-state $[CO_3^{-\bullet}]$ (molar units) under 22 W m^{-2} UV irradiance is:

$$[CO_3^{-\bullet}] = \frac{r_{CO_3^{-\bullet}}^{tot}}{k_{30} \cdot NPOC} \quad (35)$$

The function ***apex.m*** adopts a slightly different definition of $r_{CO_3^{-\bullet}}^{tot}$, namely the formation rate of $CO_3^{-\bullet}$ inside a cylinder of volume $V = 0.1\ d$ (units of mol s^{-1} instead of mol L^{-1} s^{-1}). For this reason, the term $V = 0.1\ d$ is included in the *apex.m* equation that is equivalent to (34) ($1.9 \cdot 10^{-4} = 1.9 \cdot 10^{-5}\ k_{30}\ 0.1$).

3.5. Reaction with $^1O_2$ [10]

The formation of singlet oxygen in surface waters arises from energy transfer between ground-state molecular oxygen and the excited triplet states of CDOM ($^3CDOM^*$). Accordingly, irradiated CDOM is practically the only source of $^1O_2$ in aquatic systems. In contrast, the main $^1O_2$ sink is the energy loss by collision with water molecules, to reach back ground-state O_2. The latter process has pseudo-first order rate constant $k_{^1O_2} = 2.5 \times 10^5\ s^{-1}$. Dissolved species, including dissolved organic matter that is certainly able to react with $^1O_2$, would play a minor role as $^1O_2$ sinks in aquatic systems. The main processes involving $^1O_2$ and P in surface waters would thus be the following:

$^3CDOM^* + O_2 \rightarrow CDOM + {}^1O_2$ (36)

$^1O_2 + H_2O \rightarrow O_2 + H_2O$ + heat (37)

$^1O_2$ + P → Products (38)

[10] D. Vione, R. Das, F. Rubertelli, V. Maurino, C. Minero, Modeling of indirect phototransformation processes in surface waters. In: Ideas in Chemistry and molecular Sciences: Advances in Synthetic Chemistry, Pignataro, B., ed., Wiley-VCH, Weinheim, Germany, 2010, pp. 203-234.

In the Rhône delta waters it has been found that the formation rate of $^1O_2$ by CDOM is $r_{^1O_2}^{CDOM}$ = $1.25 \cdot 10^{-3}\ P_a^{CDOM}$.[11] Considering the competition between the deactivation of $^1O_2$ by collision with the solvent (reaction 37) and reaction (38) with P, one gets the following expression for the degradation rate of P by $^1O_2$ (note that $k_{P,^1O_2} \cdot [P] \ll k_{^1O_2}$, where $k_{P,^1O_2}$ is the second-order reaction rate constant between P and $^1O_2$):

$$r_P^{^1O_2} = r_{^1O_2}^{CDOM} \cdot \frac{k_{P,^1O_2} \cdot [P]}{k_{^1O_2}} \tag{39}$$

In a pseudo-first order approximation, the rate constant of P transformation is $k_P = r_P^{^1O_2}\ [P]^{-1}$ and the half-life time is $t_P = \ln 2\ k_P^{-1}$. Considering the usual conversion (≈ 10 h) between a constant 22 W m^{-2} sunlight UV irradiance and a SSD unit, the following expression for $\tau_{P,^1O_2}^{SSD}$ is obtained (remembering that $r_{^1O_2}^{CDOM} = 1.25 \cdot 10^{-3}\ P_a^{CDOM}$, and that $P_a^{CDOM} = 10^3\ d^{-1} \int_\lambda p_a^{CDOM}(\lambda)\, d\lambda$):

$$\tau_{P,^1O_2}^{SSD} = \frac{4.81}{r_{^1O_2}^{CDOM}\ k_{P,^1O_2}} = \frac{3.85 \cdot d}{k_{P,^1O_2} \cdot \int_\lambda p_a^{CDOM}(\lambda)\, d\lambda} \tag{40}$$

Note that 3.85 = (ln 2) k_{1O2} $(1.25 \cdot 10^{-3} \cdot 3.60 \cdot 10^4 \cdot 10^3)^{-1}$. The steady-state [$^1O_2$] (molar units) under 22 W m^{-2} UV irradiance is:

$$[^1O_2] = \frac{r_{^1O_2}^{CDOM}}{k_{^1O_2}} \tag{41}$$

The function ***apex.m*** adopts a slightly different definition of $r_{^1O_2}^{CDOM}$, namely the formation rate of $^1O_2$ inside a cylinder of volume $V = 0.1\ d$ (units of mol s^{-1} instead of mol L^{-1} s^{-1}). For this reason, the term $V = 0.1\ d$ is included in the *apex.m* equation that is equivalent to (40).

[11] F. Al-Housari, D. Vione, S. Chiron, S. Barbati, Reactive photoinduced species in estuarine waters. Characterization of hydroxyl radical, singlet oxygen and dissolved organic matter triplet state in natural oxidation processes. Photochem. Photobiol. Sci. 9 (2010) 78-86.

3.6. Reaction with ^{3}CDOM* [10]

The formation of the excited triplet states of CDOM (^{3}CDOM*) in surface waters is a direct consequence of radiation absorption by CDOM itself. In aerated solution, ^{3}CDOM* could undergo thermal deactivation or reaction with O_2, and an overall pseudo-first order quenching rate constant $k_{^3CDOM*} \approx 5 \cdot 10^5$ s^{-1} has been observed. The quenching of ^{3}CDOM* is in competition with the reaction between ^{3}CDOM* and P. Moreover, ^{3}CDOM* can also be quenched by natural DOM, with a quenching/reaction rate that can be expressed as follows: $r_{^3CDOM*+DOM} = \{1700[L(mg_c)^{-1}s^{-1}]\} \times [^3CDOM*] \times DOC$, where [^{3}CDOM*] is in molar units while DOC is expressed in [mg_C L^{-1}]. Therefore, the reaction rate constant (1700) has units of L (mg_C)$^{-1}$ s^{-1}. The following processes are operational:

CDOM + hν → ^{3}CDOM*	(42)
^{3}CDOM* —(O_2)→ Deactivation and $^1O_2$ production	(43)
^{3}CDOM* + DOM → Products	(44)
^{3}CDOM* + P → Products	(45)

In the Rhône delta waters it has been found that the formation rate of ^{3}CDOM* is $r_{^3CDOM*} = 1.28 \cdot 10^{-3}\, P_a^{CDOM}$.[11] Considering the competition between reaction (45) with P and other processes (reactions 43,44), the following expression for the degradation rate of P by ^{3}CDOM* is obtained (note that $k_{P,^3CDOM*} \cdot [P] \ll k_{^3CDOM*}$, where $k_{P,^3CDOM*}$ is the second-order reaction rate constant between P and ^{3}CDOM*):

$$r_P^{^3CDOM*} = r_{^3CDOM*} \cdot \frac{k_{P,^3CDOM*} \cdot [P]}{k_{^3CDOM*} + 1700\,DOC} \quad (46)$$

In a pseudo-first order approximation, the rate constant for P transformation is $k_P = r_P^{^3CDOM*}\, [P]^{-1}$, and the half-life time is $t_P = \ln 2\; k_P^{-1}$. Considering the usual conversion (≈ 10 h) between a constant 22 W m^{-2} sunlight UV irradiance and a SSD unit, one gets the following expression for $\tau_{P,^3CDOM*}^{SSD}$ (remembering that $P_a^{CDOM} = 10^3\, d^{-1} \int_\lambda p_a^{CDOM}(\lambda)\, d\lambda$):

$$\tau_{P,^3CDOM*}^{SSD} = \frac{1.5 \cdot 10^{-5} \cdot d \cdot (k_{^3CDOM*} + 1700\,DOC)}{k_{P,^3CDOM*} \cdot \int_\lambda p_a^{CDOM}(\lambda)\, d\lambda} \quad (47)$$

Note that $1.5 \cdot 10^{-5} = (\ln 2)\,(1.28 \cdot 10^{-3} \cdot 3.60 \cdot 10^4 \cdot 10^3)^{-1}$. The steady-state [^{3}CDOM*] (molar units) under 22 W m^{-2} UV irradiance is:

$$[^3CDOM*] = \frac{r_{^3CDOM*}}{k_{^3CDOM*}} \quad (48)$$

The function ***apex.m*** adopts a slightly different definition of $r_{^3CDOM*}$, namely the formation rate of ^{3}CDOM* inside a cylinder of volume V = 0.1 d (units of mol s^{-1} instead of mol L^{-1} s^{-1}). For this reason, the term V = 0.1 d is included in the *apex.m* equation that is equivalent to (47).

3.7. Formation of intermediates [12]

In the photochemical process *ph* (direct photolysis or reaction with $^{\bullet}OH$, $^1O_2$, $CO_3^{-\bullet}$, ^{3}CDOM*), the pollutant P could produce the intermediate I with yield y_I^{ph}, experimentally determined as the ratio between the initial formation rate of I and the initial transformation rate of P. The pseudo-first order rate constant of I formation in the process *ph* is then $(k_I^{ph})' = y_I^{ph}\, k_P^{ph}$, where k_P^{ph} is the (model-derived) first-order transformation rate constant of P in the process *ph*. The production of I from P often takes place *via* more than one process. Therefore, the overall rate constant of I formation is:

$$(k_I)' = \sum_{ph} (k_I^{ph})' = \sum_{ph} (y_I^{ph}\, k_I^{ph}) \qquad (49)$$

One can also obtain the overall yield of I formation from P (y_I), as follows:

$$y_i = (k_I)'(k_P)^{-1} = \frac{\sum_{ph} (y_I^{ph}\, k_I^{ph})}{\sum_{ph} k_P^{ph}} \qquad (50)$$

The APEX code includes the calculation tools to determine intermediate formation kinetics and yields.

3.8. The meaning of water depth (optical path length of sunlight) in the model

An important issue is that the model was not designed to make depth profiles of the transformation kinetics or of the concentration of reactive transients. Therefore, when setting optical path length as a variable (which is proportional to depth, see section 6.4), one actually compares different water bodies, each with its own depth value. This means that for, *e.g.*, 1 m depth the model returns the average [$^{\bullet}OH$] (or another average photoreactivity parameter) in the first 1 m of the water column. It should be underlined that it is the average value in the first 1 m of the water column and not the point value at 1 m. One can also obtain the transformation kinetics of dissolved species in the hypothesis of thorough mixing in the water column, because the model applies to well-mixed shallow waters or to the top mixing layer of stratified water bodies. A key issue is that, if one wants

[12] E. De Laurentiis, S. Chiron, S. Kouras-Hadef, C. Richard, M. Minella, V. Maurino, C. Minero, D. Vione, Photochemical fate of carbamazepine in surface freshwaters: Laboratory measures and modelling. Environ. Sci. Technol. 46 (2012) 8164-8173.

to determine the photochemical reaction kinetics due to, *e.g.*, reaction with $^{\bullet}OH$ in the first 1 m of the water column, the needed value is the average [$^{\bullet}OH$] value (as determined by the model) and not the point [$^{\bullet}OH$] at 1 m.

3.9. Main approximations of the model

Surface waters are an extremely complex and varied series of environments, and the present attempt to describe their photochemical behaviour had to include a number of assumptions and approximations. The main ones are listed below.

- The model considers well-mixed water. Therefore, it applies to shallow water environments and to the well-mixed epilimnion of stratified lakes. However, it has recently become possible to treat the hypolimnion of stratified lakes (see the main article for instruction on how to do it).
- The Lambert-Beer approximation does not take radiation scattering into account. Therefore, the model applies to clear waters rather than to highly turbid ones. However, water turbidity changes the underwater light field but it usually does not increase much the upward reflection of sunlight (briefly, the same photons are available for photoreactions, although distributed differently with depth than in a clear water body). Therefore, the average photochemistry in a well-mixed water body is about the same, irrespective of water turbidity.
- The data on which the modelling of the surface-water absorption spectrum is based (equation 1) were obtained for lake water in NW Italy. There is evidence that applicability is much wider, but more accurate results for a particular environment can be obtained if the actual water spectrum is available (it can be used by the software, in alternative to equation 1).
- The quantum yields for the formation of $^{\bullet}OH$ by CDOM are average values for NW Italian lakes. The corresponding values for $^{1}O_2$ and $^{3}CDOM^*$ have been obtained in the Rhône delta (S. France), and the value of $CO_3^{-\bullet}$ formation from $^{3}CDOM^*$ is from Lake Greifensee (Switzerland). In different environments, different values may be found. The best scenario is obviously attained when one has data that have been measured in the studied water body, and inserts them where appropriate (*vide infra*).
- The scavenging rate constants of $^{\bullet}OH$ and $CO_3^{-\bullet}$ by DOM are average values from the literature. The same considerations as above apply here.

Despite its approximations, the model can be very useful to laboratory scientists who measure the photochemical degradation of pollutants and would like to have an assessment of the environmental significance of their findings. The possibility to model the water absorption spectrum, instead of having to use experimentally measured data could be particularly useful, if one wants to see the significance of different photochemical pathways under variable conditions.

In contrast, if one wants to describe a particular environment, the best way to increase the accuracy of the results is to use measured values from that environment. Such values are water absorption spectrum, formation quantum yields of $^{\bullet}OH$, $^{1}O_2$, $CO_3^{-\bullet}$ and $^{3}CDOM^*$ by CDOM, scavenging rate constants of $^{\bullet}OH$ and $CO_3^{-\bullet}$ by DOM, and $^{3}CDOM^*$ deactivation rate constant.

3.10. How to derive [HCO_3^-] and [CO_3^{2-}] from alkalinity and pH

The concentration values of HCO_3^- and CO_3^{2-} are often not available, unless dedicated water titration is carried out, which is relatively rare. In contrast, alkalinity (*Alk*) and pH are more common to be found.[13] The procedure to derive [HCO_3^-] and [CO_3^{2-}] from *Alk* and pH is based on the thermodynamic theory of equilibria in aqueous solution. The charge balance in surface waters is described by equation (1), where Alk is expressed in mol L^{-1} or eq L^{-1}:

$$Alk = [HCO_3^-] + 2[CO_3^{2-}] + [OH^-] - [H^+] \tag{1}$$

The acid-base equilibrium between HCO_3^- and CO_3^{2-} ($HCO_3^- \leftrightarrows CO_3^{2-} + H^+$) is described by the equilibrium constant K_{a2} = [CO_3^{2-}] [H^+] [HCO_3^-]$^{-1}$. By combining this constant with equation (1), one gets the following expressions for [HCO_3^-] and [CO_3^{2-}]:

$$[HCO_3^-] = \frac{[H^+]}{[H^+] + 2K_{a2}}(Alk + [H^+] - [OH^-]) \tag{2}$$

$$[CO_3^{2-}] = \frac{K_{a2}}{[H^+] + 2K_{a2}}(Alk + [H^+] - [OH^-]) \tag{3}$$

The values of [H^+] and [OH^-] can be easily obtained from the pH data. However, the equilibrium constant K_{a2} depends on both temperature and ionic strength. In particular, K_{a2} is related to the activity-based thermodynamic constant ($\mathbf{K}_{a2}$ = $a_{CO_3^{2-}}\, a_{H^+} (a_{HCO_3^-})^{-1}$) by the relationship K_{a2} = $\mathbf{K}_{a2}$ $\gamma_{HCO_3^-} (\gamma_{CO_3^{2-}}\, \gamma_{H^+})^{-1}$, where $\gamma_{HCO_3^-}$, $\gamma_{CO_3^{2-}}$ and γ_{H^+} are the activity coefficients of the relevant species. Moreover, $\mathbf{K}_{a2}$ depends on the temperature T according to the following relationship:[14]

$$\ln \mathbf{K}_{a2} = 207.6548 - \frac{11843.79}{T} - (33.6485)\ln T \tag{4}$$

For the calculation of the activity coefficients of the ionic compounds, the Davies equation can be used:[14]

[13] Minella, M.; Leoni, B.; Salmaso, N.; Savoye, L.; Sommaruga, R.; Vione, D. Long-term trends of chemical and modelled photochemical parameters in four Alpine lakes. *Sci. Total Environ.* **2016**, *541*, 247-256.
[14] Millero, F.J.; Pierrot, D. A chemical equilibrium model for natural waters. *Aquatic Geochem.* **1998**, *4*, 153-199.

$$\log_{10}\gamma_i = -\frac{0.5\, z_i^2 \sqrt{I}}{1+\sqrt{I}} + 0.24\, I \qquad (5)$$

where z_i is the charge of the ion *i*, and *I* is the ionic strength of the solution. The ionic strength *I* is computed as $I = \frac{1}{2}\sum_i (z_i^2[i])$, by using all the available data about inorganic ions including their concentrations values [*i*]. On this basis, $[HCO_3^-]$ and $[CO_3^{2-}]$ are calculated with an iterative method that is described in the schematic below (the value of $\mathbf{K}_{a2}$ can be obtained preliminarily by using equation (4)). Iterations are carried out till convergence. Note that, if the concentration values of the additional ionic species are not known, one can approximately consider an aqueous solution containing calcium and magnesium bicarbonates. In this case, the ionic strength can be approximated as $I = ½\ \{3\ [HCO_3^-] + 4\ [CO_3^{2-}]\}$.[15]

Within the *Apex1_1.zip* file provided as Supplementary Material, the *Savetable.m*/*Plotgraph.m* files contain by default sample values that make sense for freshwater conditions. The provided sample values are depth d = 5 m, $[NO_3^-] = 10^{-4}$ mol L^{-1}, $[NO_2^-] = 10^{-6}$ mol L^{-1}, NPOC = 2 mg_C L^{-1}, $[HCO_3^-] = 10^{-3}$ mol L^{-1}, and $[CO_3^{2-}] = 10^{-5}$ mol L^{-1}. However, users may want to replace the default values with customized ones when using the software.

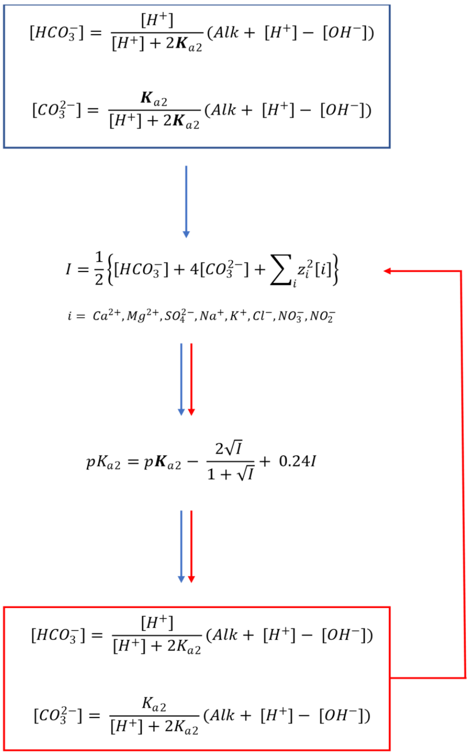

[15] Carena, L.; Terrenzio, D.; Mosley, L.M.; Toldo, M.; Minella, M.; Vione, D. Photochemical consequences of prolonged hydrological drought: A model assessment of the Lower Lakes of the Murray-Darling Basin (Southern Australia). *Chemosphere* **2019**, *236*, article n. 124356.

4. The APEX software

APEX is a tool to model photochemical processes in surface waters. It is based on Octave, an Open Source and freely available mathematics package (http://www.gnu.org/software/octave). For using APEX, Octave should be downloaded and installed. Octave has been developed for Linux, but Windows versions are also available for free download (https://wiki.octave.org/Octave_for_Microsoft_Windows), as well as versions for Mac OSX (http://wiki.octave.org/Octave_for_MacOS_X). Note that the relatively old but largely tested (with APEX) 3.2.4 Octave version for Windows is provided as a compressed *.zip* file in this SM package.

After download and installation, one has to launch Octave and to run APEX within Octave. To make this easier, it is advisable to have the Apex folder under the main root (*e.g.* as C:\Apex). An easy way to do so is to save the downloaded *Apex1_1.zip* file under C:\, and then use the "Extract to here" option of WinZip (*Apex1_1.zip* already contains the files in the \Apex folder).

APEX is based on a series of functions: *plotgraph.m* (data input and 3D graph plot), *savetable.m* (data input and generation of a table with numerical output data), *apex.m* (model calculations), *integral.m* (numerical integration). In addition, *apexvec.m* is used to produce the output format of both ***Plotgraph*** and ***Savetable***. Finally, part of the input data are contained in a *.csv* file. The scheme below gives insight into the flow of information within APEX.

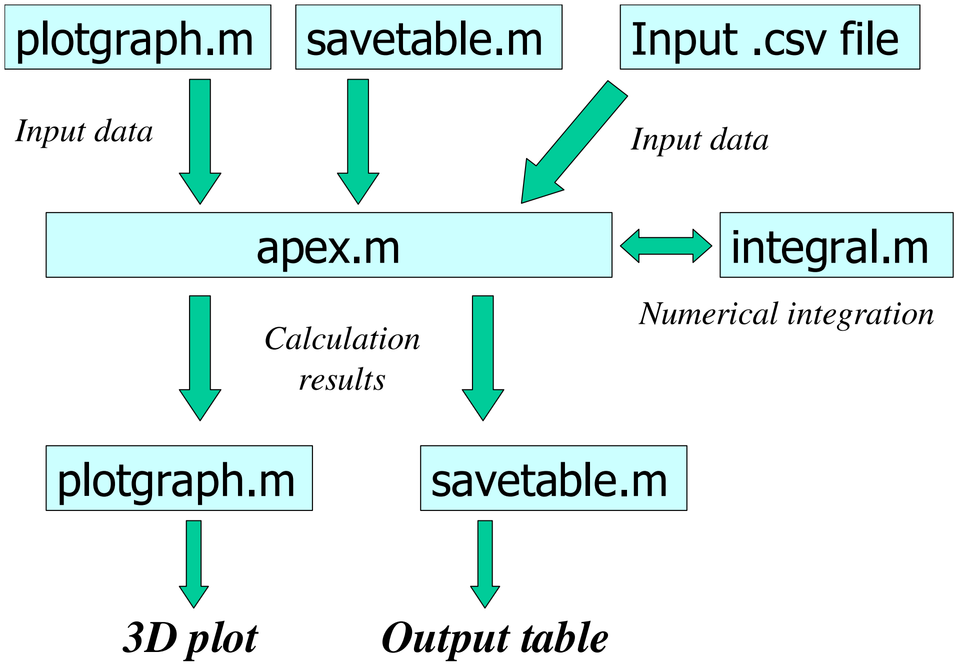

All the functions (*plotgraph.m*, *savetable.m*, *apex.m*, *integral.m* and *apexvec.m*) can be read (and modified) with a normal text editor (such as Windows' Notepad). In theory a word processor could also be used, but it is not recommended because it might add additional lines while saving the files. These lines might not be understood by Octave, and would cause the generation of error messages.

The overall procedure to apply in the use of APEX is reported below. The different parts will be explained in detail in the next chapters.

1) Create or open (to modify and/or check) the input *.csv* file (it contains data of the target compound such as absorption spectrum and photolysis quantum yield, as well as additional information).
2) Open and modify *plotgraph.m* and/or *savetable.m*, to introduce input data. **Remember to always save these files after modifying them! (And before passing to the following point).**
3) Launch Octave to create the 3D plot or the output table.

WARNING. The user needs to open the functions *plotgraph.m* and *savetable.m* to insert some of the input data. This implies a risk of accidentally causing irreversible damage to those files. For this reason, in addition to the main Apex folder, it is advisable to have a second one where to store the original APEX files after download, so that they can be quickly retrieved if needed.

NOTE: APEX has been developed in a Linux environment and it has been extensively tested with the 3.2.4 version of Octave. Some of the earlier Octave versions (*e.g.*, 2.*) might not work properly with APEX. Some later versions have been found to work properly. Successful running of APEX with Octave 3.2.4 has been carried out with Windows Me, 2000, XP, 7, 8 and 10. Other operating systems have not been tested.

5. Input files (.csv)

All the wavelength-dependent parameters that are used as input data are contained in a *.csv* file. The file contains both compound-dependent parameters, and parameters of general use. The standard adopted by default is to name the file after the compound it is referred to (this is what *molecule.csv* means in the main article). The columns present in the input file will now be listed.

Column 1: Wavelength.
Column 2: ε_{NO3-}. Molar absorption coefficient of nitrate, in L mol^{-1} cm^{-1} units.
Column 3: ε_{NO2-}. Molar absorption coefficient of nitrite, in L mol^{-1} cm^{-1} units.
Column 4: $\Phi_{NO2-/^\bullet OH}$. Quantum yield of $^\bullet$OH generation by nitrite (unitless).
Column 5: $p°_{Sun}$. Incident photon flux of sunlight at the water surface (Einstein cm^{-2} s^{-1} nm^{-1}).
Column 6: Φ. Photolysis quantum yield of the substrate (unitless).
Column 7: ε. Molar absorption coefficient of the substrate (L mol^{-1} cm^{-1}).
Column 8: A_w. Water absorbance over a 1 cm path length (cm^{-1}).

Note that data in columns 1-5 are the same for all input files, while those in columns 6,7 are referred to the relevant substrate. Column 8 reports the water spectrum if available. Therefore, if the absorption spectrum of water from the surface layer of the relevant water body has been measured, it can be placed as A_w in column 8 (units of cm^{-1}). The measure unit of A_w means that the values to be inserted are relevant to a cuvette having an optical path length of 1 cm. This is usually not the case, as such a path length is often too short to enable a precise measurement of water absorbance. If absorbance is measured with a cuvette having longer path length (*e.g.*, 5 cm or 10 cm), to obtain A_w one should divide the measured absorbance by, *e.g.*, 5 or 10, respectively.

If the water spectrum is not available, one should enter "-1" in all the cells of column 8. In this case, the absorption spectrum of water will be modelled on the basis of the DOC (or NPOC) value (entered in ***plotgraph*** and ***savetable***, *vide infra*), as $A_1(\lambda) = (0.45 \pm 0.04) \cdot NPOC \cdot e^{-(0.015 \pm 0.002) \cdot \lambda}$.

In column 6 it is possible to enter the photolysis quantum yield of the substrate as a function of wavelength, if available. If not, as it often happens, it is also possible to insert a constant value in all the column lines. Another possibility is to put "-1" throughout. In this case, the input value of the photolysis quantum yield will be given in the ***plotgraph*** and ***savetable*** functions (*vide infra*). The latter possibility allows one to assess the consequences on pollutant photodegradation of a variation of the photolysis quantum yield. In fact, inside ***plotgraph*** and ***savetable***, the photolysis quantum yield can be defined as a variable (to do so, however, one needs to have "-1" throughout in column 6 of the input *.csv* file).

The figure in the next page shows how an input file looks like.

NO_3^- absorption spectrum ⇩ | NO_2^- absorption spectrum ⇩ | Wavelength trend of •OH production by NO_2^- ⇩ | Sun spectral photon flux density (corresp. to 22 W m^{-2} UV irradiance) ⇩ | Molecule (ibuprofen) photolysis quantum yield ⇩ | Molecule (ibuprofen) molar absorption coefficient ⇩ | Water sample absorbance (b = 1 cm, optional) ⇩

Wavelength, nm	ENO3, M-1 cm-1	ENO2, M-1 cm-1	phi(NO2/OH)	p0sun, Einstein/(cm2)/s/nm	phi	EP, M-1 cm-1	Aw, cm-1
292.5	6.92	8.85	0.068	8.25E-17	3.30E-01	1.33E+01	-1
295	7.5	8.9222	0.068	1.64E-16	3.30E-01	1.11E+01	-1
297.5	7.87	8.98	0.068	3.24E-14	3.30E-01	9.50E+00	-1
300	7.9355	9.07599	0.0678	6.46E-14	3.30E-01	7.96E+00	-1
302.5	8	9.14	0.0674	8.41E-13	3.30E-01	7.14E+00	-1
305	7.7	9.29331	0.0668	1.61E-12	3.30E-01	6.62E+00	-1
307.5	7.2	9.44	0.066	4.04E-12	3.30E-01	5.78E+00	-1
310	6.7	9.66443	0.065	6.44E-12	3.30E-01	5.14E+00	-1
312.5	6	9.915	0.0635	1.21E-11	3.30E-01	4.69E+00	-1
315	5.2	10.2527	0.061	1.78E-11	3.30E-01	4.48E+00	-1
317.5	4.3	10.715	0.058	2.26E-11	3.30E-01	4.12E+00	-1
320	3.5	11.2871	0.054	2.74E-11	3.30E-01	3.84E+00	-1
322.5	2.8	11.99	0.051	3.31E-11	3.30E-01	3.71E+00	-1
325	2.1	12.78278	0.047	3.87E-11	3.30E-01	3.45E+00	-1
327.5	1.5	13.67	0.043	4.87E-11	3.30E-01	3.36E+00	-1
330	1	14.60306	0.0375	5.88E-11	3.30E-01	3.34E+00	-1
333.3	0.6	16.05	0.031	5.88E-11	3.30E-01	2.73E+00	-1
340	0	19.1156	0.026	6.63E-11	3.30E-01	2.50E+00	-1
350	0	22.59355	0.025	7.29E-11	3.30E-01	1.11E+00	-1
360	0	22.01905	0.025	7.75E-11	3.30E-01	1.11E+00	-1
370	0	16.61029	0.025	1.03E-10	3.30E-01	1.26E+00	-1
380	0	9.06147	0.025	1.14E-10	3.30E-01	7.49E-01	-1
390	0	3.41239	0.025	1.21E-10	3.30E-01	0	-1
400	0	0.82399	0.025	1.81E-10	3.30E-01	0	-1
410	0	0.17069	0.025	1.93E-10	3.30E-01	0	-1
420	0	0.06963	0.025	2.06E-10	3.30E-01	0	-1
430	0	0.05638	0.025	1.84E-10	3.30E-01	0	-1
440	0	0.05405	0.025	2.28E-10	3.30E-01	0	-1
450	0	0.05132	0.025	2.83E-10	3.30E-01	0	-1
460	0	0	0	2.83E-10	3.30E-01	0	-1
470	0	0	0	2.85E-10	3.30E-01	0	-1
[illegible]	[illegible]	[illegible]	[illegible]	[illegible]	[illegible]	[illegible]	[illegible]

[...] The file continues till 800 nm

The figure above is cut at 470 nm, but the actual wavelength interval is 292.5-800 nm.

It is advisable to have the input *.csv* file in the same folder as the other APEX files (*i.e.*, in C:\Apex). In the version provided here the only input file in the " C:\Apex " folder is "*Ibuprofen.csv*". However, input files for 44 compounds are contained in the "Compounds" folder (C:\Apex\Compounds). To use a file, transfer it to the main Apex folder (*i.e.*, copy the file from C:\Apex\Compounds to C:\Apex).

It is useful to spend some words on how the photolysis quantum yield can be dealt with in APEX. The program has been designed to manage the quantum yield at different levels of knowledge by the user. In other words, the user may know the dependence of the quantum yield on the wavelength ("full knowledge"), or just one average value over wavelength (or maybe, the quantum yield is really constant in the wavelength interval of sunlight absorption by the substrate). In some unfortunate cases, the quantum yield could be totally unknown. Depending on the level of knowledge, APEX enables four different approaches to the problem (but note that two of them are equivalent):

1) If one knows the values of the photolysis quantum yield as a function of the wavelength, these values can be inserted in the 6th column (labelled "phi") of the input *.csv* file (*molecule.csv*). This is the most favourable scenario because photolysis is computed at each wavelength, also considering depth penetration of sunlight at that wavelength, and the competition for irradiance between the solute and other solution components at that wavelength.
2) If one knows only a single average value of the photolysis quantum yield (or if the quantum yield is really constant with wavelength), one can enter the same constant value in the whole 6th column of the input file. This is the case for *Ibuprofen.csv*, for instance.
3) As an alternative to 2), one can put "-1" overall in the 6th column of *molecule.csv*, and add the quantum yield value within ***plotgraph*** or ***savetable*** (see the next section 5). The result will be exactly the same as in point 2) above.
4) If the quantum yield is unknown, one might wish to see how would the substrate transformation kinetics be modified for different possible values of the quantum yield itself. To do so, one should put "-1" in the whole 6th column of the input file, and define the quantum yield as a variable within ***plotgraph*** or ***savetable*** (see the next section 5 for this).

An important issue is that there is a hierarchy in the quantum yield data. If quantum yield values are inserted both in the input file and in ***plotgraph*** or ***savetable***, the program will read only the values in the *.csv* input file and ignore the others. Therefore, if one wants to insert the photolysis quantum yield within ***plotgraph*** or ***savetable***, or define it as a variable, it is necessary that the 6th column ("*phi*") of the input .csv file has "-1" throughout.

6. Plotgraph and Savetable

These are the two main functions of APEX that users will most frequently access. They are basically two different ways to present the same output data. ***Plotgraph*** is intended to produce a 3D plot that shows how the selected output parameter (first-order rate constant, half-life time, intermediate formation yield, etc.; *vide infra* for the full list of the 36 possible output variables) varies as a function of a couple of selected variables (data of water chemistry and depth, or reaction rate constants of the pollutant). ***Savetable*** reports, in a *.csv* output file, all the possible output parameters as a function of that couple of variables. Users are expected to open either "*plograph.m*" or "*savetable.m*" (they can be opened with any text editor, *e.g.* Windows' Notepad®) and modify the editable parts. In fact, all the wavelength-independent input data should be inserted here. The two functions will now be described with more detail.

6.1. Plotgraph (file plotgraph.m)

Plotgraph consists of a series of input blocks. They deal with variable (X,Y) range, name of input *.csv* file (this is *molecule.csv*), numerical parameters and output selection. The rest of the ***plotgraph.m*** file (beginning from the quantum yields of formation of $^{\bullet}OH$, $CO_3^{-\bullet}$, $^1O_2$ and $^3CDOM^*$ from CDOM) should not be modified by most users, unless they are very experienced in both CDOM photochemistry and Octave programming. Warning notices are present in the relevant parts of the code to discourage careless use. The various input blocks will now be commented.

6.1.1. Range input

This section appears as follows (the APEX code is here in blue, and data to be modified by the user are highlighted in yellow; note that APEX is widely commented, and that all the comments begin with "%". By adding "%", the relevant line will not be read by the software when running):

```
% ============================================================
% *** BEGINNING OF RANGE INPUT ***

  x=0.2:0.2:10;  % Range for the first variable, A:B:C means from A to C with steps of B.
  y=0.1:0.1:8;    % Range for the second variable.

% *** END OF RANGE INPUT ***
% ============================================================
```

This means that here one should select the X and Y range (minimum, variation step, and maximum). Some lines below, the user will be asked to select which variables are X and Y in the 3D graph, as a function of which the Z values will be computed and plotted. The selection of Z takes place further below. Note that three numbers should be entered here for each variable, saparated by colons: they are the minimum value, the step and the maximum value of the variable,

respectively. Therefore, "0.2:0.2:10" means from 0.2 to 10 with steps of 0.2 (in other words, the software will make calculations for the X values of 0.2, 0.4, 0.6 etc., up to 10). It is also possible to write something like "0.1:0.2:10". In this case, the X values will be 0.1, 0.3, 0.5 etc. The sequence would end with 9.9 and 10.1 and, because 10.1 is already too large, the last value for computations will be 9.9. The calculation engine will accommodate this without problems or error messages. Note that the smaller the step, the more accurate and "coloured" the final plot will be (*vide infra*). However, computation time will increase accordingly. In most cases the actual choice will be a compromise between scientific requirements, aestetics, processor speed and available time.

6.1.2. Input file prefix

The name of the .csv input file should be given here. Note that "name" means that the relevant file is "filename.csv" (i.e., *molecule.csv*). The input file *molecule.csv* should be placed in the same folder as ***plotgraph*** (for Windows users, the folder may have the path C:\Apex).

```
% ==================================================================
% *** INPUT FILE PREFIX ***
  % Here you should insert the name of the input file, which reports the spectra of the compound,
  % sunlight and water, as well as the photolysis quantum yield.
  % file_prefix = prefix for filenames, named <file_prefix>"_LL.csv" ...

  file_prefix = "Ibuprofen";

% *** END OF INPUT FILE PREFIX ***
% ==================================================================
```

The above entry means that the input file is "Ibuprofen.csv".

6.1.3. Data input

Here users should specify the numerical values of: column depth, *i.e.*, optical path length of sunlight within the water body; concentration of photochemically relevant parameters (they are to be expressed in **molarity –mol L^{-1}–**, with the exception of the NPOC –a synonym for the DOC– that is expressed in **ppm of carbon**, that is, **mg$_C$ L^{-1}**),[16] as well as the second-order reaction rate constants of the studied compound with $^{\bullet}$OH, $CO_3^{-\bullet}$, $^1O_2$ and ^{3}CDOM* (units of **L mol^{-1} s^{-1}**). It is also possible to model the formation of an intermediate by inserting its (unitless) formation yields from the substrate upon direct photolysis and reaction with $^{\bullet}$OH, $CO_3^{-\bullet}$, $^1O_2$ and ^{3}CDOM* (see section 3.7 for the relevant theory). Among the input data, users should choose two variables that will be the X and Y of the 3D plot. These variables will vary within the range defined above (section 6.1.1). To make the program recognise such variables, X should be indicated by "-1" and Y by "-2". The relevant section of the ***plotgraph*** function is reported in the next page, where the optical path length *d* is set as X and NPOC as Y.

[16] Note that water concentration data are often given in mg L^{-1} (for nitrate, it may be mg L^{-1} of N (NO3-N) or of NO3, and a similar issue holds for nitrite). Whatever they mean exactly, the mg L^{-1} data should be converted into molarity (mol L^{-1}) by users to enable proper kinetic calculations.

In the case of fi_P (photolysis quantum yield, unitless), the value inserted here will be read by the program only if the relevant column of the input .csv file (column 6, see section 4) has “-1” values throughout. Otherwise, the value inserted here will be ignored and the program will use the data of the input file. The possibility to insert fi_P here is interesting if one wants to define fi_P as the X or Y variable (*e.g.*, because the quantum yield is unknown), and to see the effects of its variation.

The code lines concerning data input are here reported (example data are referred to ibuprofen and to its transformation into 4-isobytylacetophenone, as a function of path length (X) and NPOC (Y)).[12] For the proportionality between the optical path length of sunlight and water depth, see section 6.3. Note that "1e-4" means 1×10^{-4}, and so on.

```
% ======================================================================
% *** BEGINNING OF DATA INPUT ***
% NOTE: "-1" denotes the x variable, "-2" the y variable (see above for their ranges)

  % d = optical path length of sunlight in water (metres)
  % See the User's Guide (section 5.3) for the relationship between optical path length and water depth
  d = -1;

  % CNO3 = Concentration of NO3- (nitrate, molarity)
  CNO3 = 1e-4;

  % CNO2 = Concentration of NO2- (nitrite, molarity)
  CNO2 = 1e-6;

  % NPOC = Dissolved Organic Carbon (DOC or NPOC, mgC/L or ppmC)
  NPOC = -2;

  % CCO3 = Concentration of CO3 2- (carbonate, molarity)
  CCO3 = 1e-5;

  % CHCO3 = Concentration of HCO3- (bicarbonate, molarity)
  CHCO3 = 1e-3;

  % CBr = Concentration of Br- (bromide, molarity)
  CBr = 1e-11;

  % kP_OH = Second-order reaction rate constant between P and °OH (units of molarity^-1 seconds^–1, 0 if
            not available)
  kP_OH = 1e10;

  % kP_CO3 = Second-order reaction rate constant between P and CO3-° (units of molarity^–1 seconds^–1,
             0 if not available)
  kP_CO3 = 1

  % kP_DOM = Second-order reaction rate constant between P and 3CDOM* (units of molarity^–1
             seconds^–1, 0 if not available)
  kP_DOM = 4.5e7

  % kP_1O2 = Second-order reaction rate constant between P and 1O2 (units of molarity^–1 seconds^–1, 0 if
            not available)
  kP_1O2 = 6e4
```

```
% fi_P = direct photolysis quantum yield of P (unitless, 0 if not available)
% Note that the software will read with priority the data reported in the input .csv file.
% To enable the use of fi_P here, the whole relevant column in the .csv file should read "-1"
fi_P = 0.33;

% y_OH = yield of the intermediate via the °OH pathway (unitless, [formation rate of the intermediate via
        °OH]/[transformation rate of P])
y_OH = 0.023;

% y_CO3 = yield of the intermediate via the CO3-° pathway (unitless, [formation rate of the intermediate
        via CO3-°]/[transformation rate of P])
y_CO3 = 1e-10;

% y_1O2 = yield of the intermediate via the 1O2 pathway (unitless, [formation rate of the intermediate via
        1O2]/[transformation rate of P])
y_1O2 = 1e-10;

% y_3DOM = yield of the intermediate via the 3CDOM* pathway (unitless, [formation rate of the
        intermediate via 3CDOM*]/[transformation rate of P])
y_3DOM = 0.31;

% y_Phot = yield of the intermediate via the direct photolysis (unitless, [formation rate of the intermediate
        via direct photolysis]/[transformation rate of P])
y_Phot = 0.25;

% *** END OF DATA INPUT ***
% ==================================================================
```

Note that any of the above variables can be defined as X (-1) or Y (-2), thus it is possible to see the effects of a variation of depth, chemical composition, P reaction rate constants and quantum yield, as well as formation yields of the intermediate from P. Also note that defining kP_CO3 = 1, y_CO3 = 1e-10 and y_1O2 = 1e-10 are safe ways to consider the relevant values as negligible.

Reasonably complete sets of rate constants have already been measured for some compounds, and some available values are reported in Table 1 (pages 11-13). The corresponding wavelength-dependent input files (of the kind *molecule.csv*) are included in the "Compounds" folder as explained above.

6.1.4. Output selection

In this section, users define the Z variable of the 3D plot. There is a choice between 36 different variables, namely: half-life times, either total or referred to a single photochemical pathway (direct photolysis and reaction with $^{\bullet}OH$, $CO_3^{-\bullet}$, $^1O_2$ and $^3CDOM^*$); first-order rate constants of substrate transformation (both total and pathway-specific); steady-state concentrations of $^{\bullet}OH$, $CO_3^{-\bullet}$, $^1O_2$ and $^3CDOM^*$ (these are independent of the chosen substrate); rate constants and yields of intermediate formation; relative role of the different processes in substrate transformation and intermediate formation (*i.e.*, the fractions accounted for by $^{\bullet}OH$, $CO_3^{-\bullet}$, $^1O_2$ and $^3CDOM^*$). Finally, the fractions of $^{\bullet}OH$ formation accounted for by nitrate, nitrite and CDOM (this is used to compute model errors, *vide infra*). The variable choice is done by inserting the corresponding number, see below.

```
% ===========================================================================
% *** BEGINNING OF OUTPUT SELECTION ***

 % The function returns results as a vector in the following order
 %  1) t_OH  = The half-life time of P with .OH in Summer Sunny Days (SSD)
 %  2) t_CO3 = The half-life time of P with CO3-. in Summer Sunny Days (SSD)
 %  3) t_1O2 = The half-life time of P with 1O2 in Summer Sunny Days (SSD)
 %  4) t_3DOM = The half-life time of P with CDOM* in Summer Sunny Days (SSD)
 %  5) t_Phot = The half-life time of P by direct photolysis in Summer Sunny Days (SSD)
 %  6) t_tot = The overall half-life time of P in Summer Sunny Days (SSD)
 %  7) k_OH = The first-order rate constant of P for reaction with .OH (1/SSD)
 %  8) k_CO3 = The rate constant of P for reaction with CO3-. (1/SSD)
 %  9) k_1O2 = The rate constant of P for reaction with 1O2 (1/SSD)
 % 10) k_3DOM = The rate constant of P for reaction with CDOM* (1/SSD)
 % 11) k_Phot = The rate constant of P upon direct photolysis (1/SSD)
 % 12) k_tot = The overall rate constant for P degradation (1/SSD)
 % 13) coOH = The steady-state [.OH] in mol/L (22 w/m2 UV irradiance)
 % 14) coCO3 = The steady-state [CO3-.] in mol/L (22 w/m2 UV irradiance)
 % 15) co1O2 = The steady-state [1O2] in mol/L (22 w/m2 UV irradiance)
 % 16) co3DOM = The steady-state [CDOM*] in mol/L (22 w/m2 UV irradiance)
 % 17) f_OH = The first-order rate constant of intermediate formation upon reaction of P with .OH (1/SSD)
 % 18) f_CO3 = The rate constant of intermediate formation upon reaction of P with CO3-. (1/SSD)
 % 19) f_1O2 = The rate constant of intermediate formation upon reaction of P with 1O2 (1/SSD)
 % 20) f_3DOM = The rate constant of intermediate formation upon reaction of P with CDOM* (1/SSD)
 % 21) f_Phot = The rate constant of intermediate formation from P by direct photolysis (1/SSD)
 % 22) f_tot = The overall rate constant of intermediate formation (1/SSD)
 % 23) y_tot = Overall formation yield of the intermediate from P
 % 24) role_OH_P = Fraction of P transformation that is accounted for by .OH
 % 25) role_CO3_P = Fraction of P transformation that is accounted for by CO3-.
 % 26) role_1O2_P = Fraction of P transformation that is accounted for by 1O2
 % 27) role_3DOM_P = Fraction of P transformation that is accounted for by 3DOM
 % 28) role_Phot_P = Fraction of P transformation that is accounted for by direct photolysis.
 % 29) role_OH_I = Fraction of intermediate formation that is accounted for by .OH
 % 30) role_CO3_I = Fraction of intermediate formation that is accounted for by CO3-.
 % 31) role_1O2_I = Fraction of intermediate formation that is accounted for by 1O2.
 % 32) role_3DOM_I = Fraction of intermediate formation that is accounted for by 3DOM.
 % 33) role_Phot_I = Fraction of intermediate formation that is accounted for by direct photolysis.
 % 34) NO3_OH = Fraction of .OH formation accounted for by nitrate
 % 35) NO2_OH = Fraction of .OH formation accounted for by nitrite
 % 36) DOM_OH = Fraction of .OH formation accounted for by CDOM)
 % Select the Z variable to be plotted as a function of X and Y (e.g.: var_to_plot = 6, means t_tot will be
   plotted).

 var_to_plot = 6;

% *** END OF OUTPUT SELECTION ***
% ===========================================================================
```

With d = -1, NPOC = -2 and var_to_plot = 6, t_tot (the overall half-life time of substrate transformation, units of SSD) will be plotted as a function of depth and NPOC. With all of the above choices, a plot will be obtained of the photochemical half-life time of ibuprofen as a function of path length and NPOC (*i.e.*, DOC), with constant 0.1 mM nitrate, 1 µM nitrite, 1 mM bicarbonate, 10 µM carbonate, and 0.01 nM bromide (*i.e.*, negligible bromide).

For better/additional clarity, the figures below show how the ***plotgraph.m*** file looks like overall. The added comments are intended as an aid to identify the different parts more easily. The figure in this page shows the initial lines of the file (***savetable.m*** looks the same, differences occur in the parts below that are shown -for ***plotgraph***- in the next page).

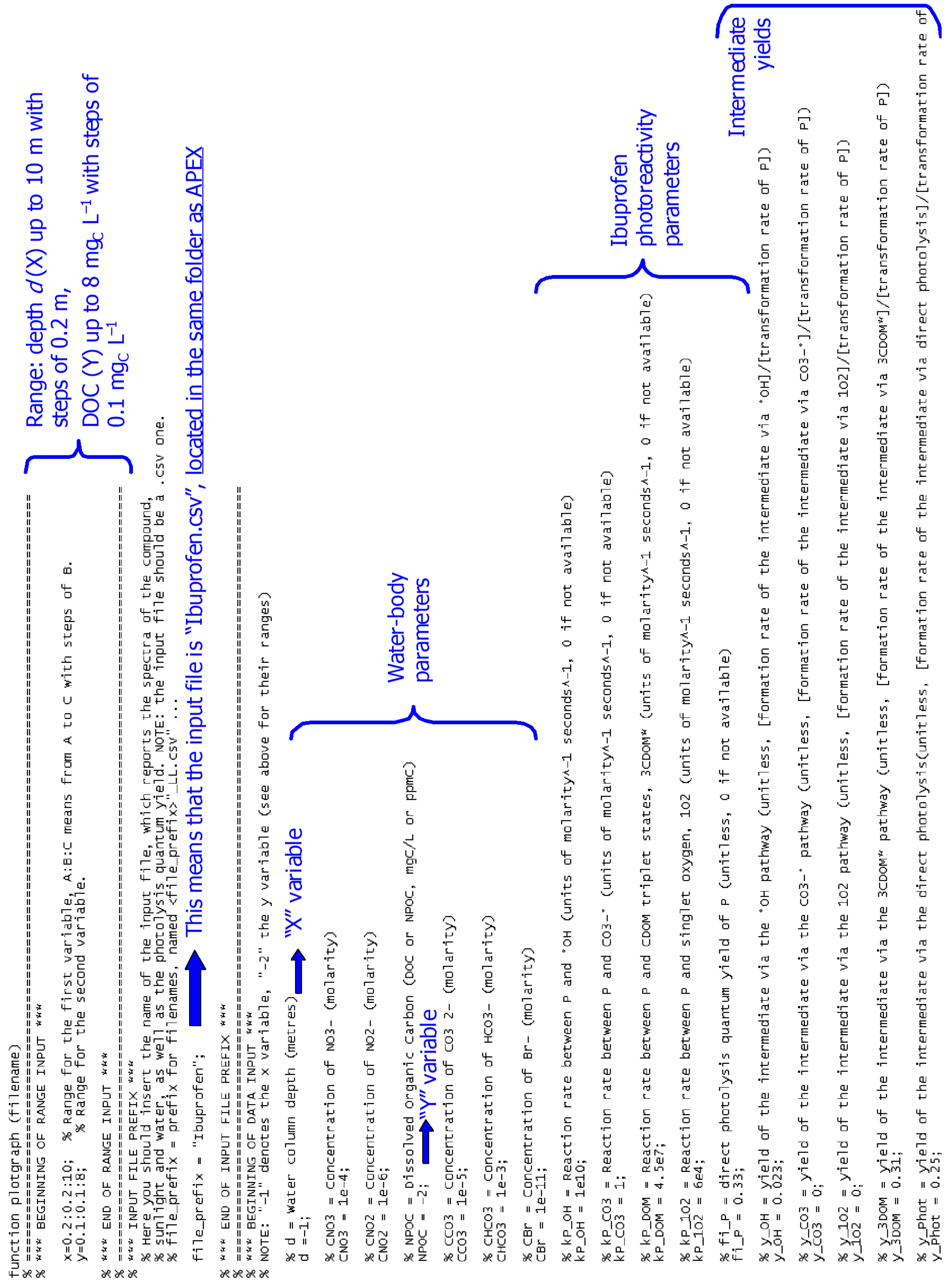

```
function plotgraph (filename)
% ==========================================================================================
% *** BEGINNING OF RANGE INPUT ***

  x=0.2:0.2:10;   % Range for the first variable, A:B:C means from A to C with steps of B.
  y=0.1:0.1:8;      % Range for the second variable.

% *** END OF RANGE INPUT ***
% ==========================================================================================
% *** INPUT FILE PREFIX ***
  % Here you should insert the name of the input file, which reports the spectra of the compound,
  % sunlight and water, as well as the photolysis quantum yield. NOTE: the input file should be a .csv one.
  % file_prefix = prefix for filenames, named <file_prefix>"_LL.csv" ...

  file_prefix = "Ibuprofen";

% *** END OF INPUT FILE PREFIX ***
% ==========================================================================================
% *** BEGINNING OF DATA INPUT ***
% NOTE: "-1" denotes the x variable, "-2" the y variable (see above for their ranges)

  % d = Water column depth (metres)
  d =-1;

  % CNO3 = Concentration of NO3- (molarity)
  CNO3 = 1e-4;

  % CNO2 = Concentration of NO2- (molarity)
  CNO2 = 1e-6;

  % NPOC = Dissolved Organic Carbon (DOC or NPOC, mgC/L or ppmC)
  NPOC = -2;

  % CCO3 = Concentration of CO3 2- (molarity)
  CCO3 = 1e-5;

  % CHCO3 = Concentration of HCO3- (molarity)
  CHCO3 = 1e-3;

  % CBr = Concentration of Br- (molarity)
  CBr = 1e-11;

  % kP_OH = Reaction rate between P and •OH (units of molarity^-1 seconds^-1, 0 if not available)
  kP_OH = 1e10;

  % kP_CO3 = Reaction rate between P and CO3-• (units of molarity^-1 seconds^-1, 0 if not available)
  kP_CO3 = 1;

  % kP_DOM = Reaction rate between P and CDOM triplet states, 3CDOM* (units of molarity^-1 seconds^-1, 0 if not available)
  kP_DOM = 4.5e7;

  % kP_1O2 = Reaction rate between P and singlet oxygen, 1O2 (units of molarity^-1 seconds^-1, 0 if not available)
  kP_1O2 = 6e4;

  % fi_P = direct photolysis quantum yield of P (unitless, 0 if not available)
  fi_P = 0.33;

  % y_OH = yield of the intermediate via the •OH pathway (unitless, [formation rate of the intermediate via •OH]/[transformation rate of P])
  y_OH = 0.023;

  % y_CO3 = yield of the intermediate via the CO3-• pathway (unitless, [formation rate of the intermediate via CO3-•]/[transformation rate of P])
  y_CO3 = 0;

  % y_1O2 = yield of the intermediate via the 1O2 pathway (unitless, [formation rate of the intermediate via 1O2]/[transformation rate of P])
  y_1O2 = 0;

  % y_3DOM = yield of the intermediate via the 3CDOM* pathway (unitless, [formation rate of the intermediate via 3CDOM*]/[transformation rate of P])
  y_3DOM = 0.31;

  % y_Phot = yield of the intermediate via the direct photolysis(unitless, [formation rate of the intermediate via direct photolysis]/[transformation rate of
  y_Phot = 0.25;
```

This is how the ***plotgraph.m*** file continues, below the part shown in the previous page.

```
% ==========================================================================================
% *** BEGINNING OF OUTPUT SELECTION ***

  % The function returns results as a vector in the following order
  %  1) t_OH  = The half-life time of P with .OH in Summer Sunny Days (SSD)
  %  2) t_CO3 = The half-life time of P with CO3-. in Summer Sunny Days (SSD)
  %  3) t_1O2 = The half-life time of P with 1O2 in Summer Sunny Days (SSD)
  %  4) t_3DOM = The half-life time of P with 3CDOM* in Summer Sunny Days (SSD)
  %  5) t_Phot = The half-life time of P by direct photolysis in Summer Sunny Days (SSD)
  %  6) t_tot = The overall half-life time of P in Summer Sunny Days (SSD)
  %  7) k_OH = The first-order rate constant of P for reaction with .OH (1/SSD)
  %  8) k_CO3 = The rate constant of P for reaction with CO3-. (1/SSD)
  %  9) k_1O2 = The rate constant of P for reaction with 1O2 (1/SSD)
  % 10) k_3DOM = The rate constant of P for reaction with 3CDOM* (1/SSD)
  % 11) k_Phot = The rate constant of P upon direct photolysis (1/SSD)
  % 12) k_tot = The overall rate constant for P degradation (1/SSD)
  % 13) cOOH = The steady-state [.OH] in mol/L (22 W/m2 UV irradiance)
  % 14) cOCO3 = The steady-state [CO3-.] in mol/L (22 W/m2 UV irradiance)
  % 15) cO1O2 = The steady-state [1O2] in mol/L (22 W/m2 UV irradiance)
  % 16) cO3DOM = The steady-state [3CDOM*] in mol/L (22 W/m2 UV irradiance)
  % 17) f_OH = The first-order rate constant of intermediate formation upon reaction of P with .OH (1/SSD)
  % 18) f_CO3 = The rate constant of intermediate formation upon reaction of P with CO3-. (1/SSD)
  % 19) f_1O2 = The rate constant of intermediate formation upon reaction of P with 1O2 (1/SSD)
  % 20) f_3DOM = The rate constant of intermediate formation upon reaction of P with 3CDOM* (1/SSD)
  % 21) f_Phot = The rate constant of intermediate formation from P by direct photolysis (1/SSD)
  % 22) f_tot = The overall rate constant of intermediate formation (1/SSD)
  % 23) y_tot = overall formation yield of the intermediate from P
  % 24) role_OH_P = Fraction of P transformation that is accounted for by .OH
  % 25) role_CO3_P = Fraction of P transformation that is accounted for by CO3-.
  % 26) role_1O2_P = Fraction of P transformation that is accounted for by 1O2
  % 27) role_3DOM_P = Fraction of P transformation that is accounted for by 3CDOM*
  % 28) role_Phot_P = Fraction of P transformation that is accounted for by direct photolysis.
  % 29) role_OH_I = Fraction of intermediate formation that is accounted for by .OH
  % 30) role_CO3_I = Fraction of intermediate formation that is accounted for by CO3-.
  % 31) role_1O2_I = Fraction of intermediate formation that is accounted for by 1O2.
  % 32) role_3DOM_I = Fraction of intermediate formation that is accounted for by 3CDOM*.
  % 33) role_Phot_I = Fraction of intermediate formation that is accounted for by direct photolysis.
  % 34) NO3_OH = Fraction of .OH formation accounted for by nitrate
  % 35) NO2_OH = Fraction of .OH formation accounted for by nitrite
  % 36) DOM_OH = Fraction of .OH formation accounted for by CDOM)
  % Select the variable to be plotted (es: var_to_plot = 6, means t_tot will be plotted).

  var_to_plot = 6;

% *** END OF OUTPUT SELECTION ***
% ==========================================================================================


% ------------------------------------------------------------------------------------------
% === INPUT OF QUANTUM YIELD VALUES FOR REACTIVE SPECIES PHOTOPRODUCTION BY CDOM (ONLY FOR EXPERT USERS!!!) ===
% NOTE: MODIFY THESE VALUES ONLY IF YOU DEFINITELY KNOW WHAT YOU ARE DOING!!!!

  qyieldOH_CDOM = 3e-5;
  carbonateyieldCO3_CDOM = 6.5e-3;      % NOTE: The relevant equation is: R_CO3_CDOM = carbonateyieldCO3_CDOM * CCO3 * PaCDOM
  qyield1O2_CDOM = 1.25e-3;
  qyieldTriplet_CDOM = 1.28e-3;

% === END OF INPUT OF QUANTUM YIELD VALUES
% ------------------------------------------------------------------------------------------


%*****************************************************************************************
% ***** DO NOT TRY TO MODIFY THE FILE BELOW THIS POINT, UNLESS YOU ARE AN EXPERIENCED OCTAVE PROGRAMMER *****
%*****************************************************************************************
```

List of the dependent variables that can be computed and plotted on the Z-axis of the 3D graph, as a function of the chosen X and Y

Entering a number from 1 to 36 selects the Z-variable to be plotted ("6" means overall half-life time)

Formation quantum yields of reactive species by irradiated CDOM

The part below (not shown) makes the plot. It is highly advisable not to modify it

MOST USERS WILL END HERE THEIR EDITING OF PLOTGRAPH. The part located below the choice of Z deals with the quantum yields of formation of $^{\bullet}OH$, $CO_3^{-\bullet}$, $^1O_2$ and $^3CDOM^*$ by CDOM. They should be modified only if reliable data are available for the particular environment under study (section 6.1.5). In all the other cases, it is better not to alter the existing values. Further below there is the procedure to make the plot, which is highly advisable not to modify inadvertently.

6.1.5. Quantum yields ($^{\bullet}OH$, $CO_3^{-\bullet}$, $^1O_2$ and $^3CDOM^*$)

If data describing a particular water body are available, quantum yields could be entered by the user in this section. Note that the formation rate of $CO_3^{-\bullet}$ upon carbonate oxidation by $^3CDOM^*$ also depends on the carbonate concentration, thus in this case the "quantum yield" has a different meaning. This issue should be considered when inserting the relevant datum.

The meaning of the relevant variables is as follows:

qyieldOH_CDOM	Quantum yield of $^{\bullet}OH$ formation by CDOM
qyield1O2_CDOM	Quantum yield of $^1O_2$ formation by CDOM
qyieldTriplet_CDOM	Quantum yield of $^3CDOM^*$ formation by CDOM
carbonateyieldCO3_CDOM	It describes the formation of $CO_3^{-\bullet}$ by $^3CDOM^*$ and CO_3^{2-}. It also depends on carbonate concentration. The formula of $CO_3^{-\bullet}$ formation rate is as follows: R_CO3_CDOM = = carbonateyieldCO3_CDOM × CCO3 × PaCDOM

The relevant lines of the code are reported below.

```
% -------------------------------------------------------------------------------------------
% === INPUT OF QUANTUM YIELD VALUES FOR REACTIVE SPECIES PHOTOPRODUCTION BY
    CDOM (ONLY FOR EXPERT USERS!!!) ===
% NOTE: MODIFY THESE VALUES ONLY IF YOU DEFINITELY KNOW WHAT YOU ARE
    DOING!!!!

 qyieldOH_CDOM = 3e-5;
 carbonateyieldCO3_CDOM = 6.5e-3;          % The relevant equation is: R_CO3_CDOM =
                                           carbonateyieldCO3_CDOM * CCO3 * PaCDOM
 qyield1O2_CDOM = 1.25e-3;
 qyieldTriplet_CDOM = 1.28e-3;

% === END OF INPUT OF QUANTUM YIELD VALUES
% -------------------------------------------------------------------------------------------
```

The rest of the ***plotgraph*** function makes the 3D plot. It should not be modified, unless users are expert in Octave programming.

6.2 Savetable (file savetable.m)

The function ***savetable*** is similar to ***plotgraph***, with the difference that it returns the output data in the form of a numerical table instead of a 3D plot. The input features are very similar to those already seen for ***plotgraph***: the code for *Range input*, *Input file prefix* and *Data input* is exactly the same, including the choice of the X and Y variables. ***Savetable*** does **not** include the *Output selection* section, because the table that is generated reports the data for all the 36 output variables. Such a table file contains 38 columns, namely X, Y and t_OH…DOM_OH. The same issues as for ***plotgraph*** apply to the *Quantum yields* section (6.1.5), because modifications of the quantum yield values is recommended only in the presence of known/reliable environmental data.

The table below reports the names of the output variables (in the order they appear in the file and grouped by typology: half-life times, first-order rate constants etc.) as well as a brief description.

Variable	*Description*
t_OH	Half-life time of P degradation by $^{\bullet}OH$ (SSD)
t_CO3	Half-life time of P degradation by $CO_3^{-\bullet}$ (SSD)
t_1O2	Half-life time of P degradation by $^1O_2$ (SSD)
t_3DOM	Half-life time of P degradation by $^3CDOM^*$ (SSD)
t_Phot	Half-life time of P degradation by (direct) photolysis (SSD)
t_tot	Overall half-life time of P degradation (SSD)
k_OH	First-order rate constant of P degradation by $^{\bullet}OH$ (SSD^{-1})
k_CO3	First-order rate constant of P degradation by $CO_3^{-\bullet}$ (SSD^{-1})
k_1O2	First-order rate constant of P degradation by $^1O_2$ (SSD^{-1})
k_3DOM	First-order rate constant of P degradation by $^3CDOM^*$ (SSD^{-1})
k_Phot	First-order rate constant of P degradation by photolysis (SSD^{-1})
k_tot	Overall first-order rate constant of P degradation (SSD^{-1})
coOH	Steady-state [$^{\bullet}OH$] (mol L^{-1}, 22 W m^{-2} UV irradiance)
coCO3	Steady-state [$CO_3^{-\bullet}$] (mol L^{-1}, 22 W m^{-2} UV irradiance)
co1O2	Steady-state [$^1O_2$] (mol L^{-1}, 22 W m^{-2} UV irradiance)
co3DOM	Steady-state [$^3CDOM^*$] (mol L^{-1}, 22 W m^{-2} UV irradiance)
f_OH	First-order rate constant of I formation by $^{\bullet}OH$ (SSD^{-1})
f_CO3	First-order rate constant of I formation by $CO_3^{-\bullet}$ (SSD^{-1})
f_1O2	First-order rate constant of I formation by $^1O_2$ (SSD^{-1})
f_3DOM	First-order rate constant of I formation by $^3CDOM^*$ (SSD^{-1})
f_Phot	First-order rate constant of I formation by photolysis (SSD^{-1})
f_tot	Overall first-order rate constant of I formation (SSD^{-1})
y_tot	Overall formation yield of I from P (unitless)
role_OH_P	Role of $^{\bullet}OH$ in P degradation (fraction of total reaction, unitless)
role_CO3_P	Role of $CO_3^{-\bullet}$ in P degradation (fraction of total reaction, unitless)
role_1O2_P	Role of $^1O_2$ in P degradation (fraction of total reaction, unitless)

role_3DOM_P	Role of ^{3}CDOM* in P degradation (fraction, unitless)
role_Phot_P	Role of direct photolysis in P degradation (fraction, unitless)
role_OH_I	Role of $^{\bullet}$OH in I formation (fraction of total reaction, unitless)
role_CO3_I	Role of $CO_3^{-\bullet}$ in I formation (fraction of total reaction, unitless)
role_1O2_I	Role of $^{1}O_2$ in I formation (fraction of total reaction, unitless)
role_3DOM_I	Role of ^{3}CDOM* in I formation (fraction of total reaction, unitless)
role_Phot_I	Role of photolysis in I formation (fraction of total reaction, unitless)
NO3_OH	Fraction of $^{\bullet}$OH produced by nitrate photolysis (unitless)
NO2_OH	Fraction of $^{\bullet}$OH produced by nitrite photolysis (unitless)
DOM_OH	Fraction of $^{\bullet}$OH produced by CDOM (unitless)

The ***savetable*** function creates an output table. To do so one has to run ***savetable*** within Octave, which will be explained in the next chapter. The figure below shows how the output file looks like.

X	Y	t_OH	t_CO3	t_1O2	t_3DOM	t_Phot	t_tot	k_OH	k_CO3	k_1O2	k_3DOM	k_Phot	k_tot
0.1	0.1	3.321679	16089151	2542855	903.0025	7.261395	2.273373	0.208674	4.31E-08	2.73E-07	0.000768	0.095456	0.304898
0.1	0.2	3.427707	32757483	1273247	452.1475	7.279061	2.318394	0.202219	2.12E-08	5.44E-07	0.001533	0.095225	0.298977
0.1	0.3	3.533256	49984294	850044.3	301.8623	7.296737	2.361908	0.196178	1.39E-08	8.15E-07	0.002296	0.094994	0.293469
0.1	0.4	3.63833	67750475	638442.4	226.7196	7.314423	2.403963	0.190512	1.02E-08	1.09E-06	0.003057	0.094764	0.288335
0.1	0.5	3.742933	86038366	511481	181.6339	7.332117	2.444605	0.185188	8.06E-09	1.36E-06	0.003816	0.094536	0.283542
0.1	0.6	3.847067	1.05E+08	426839.8	151.5766	7.349821	2.483881	0.180176	6.61E-09	1.62E-06	0.004573	0.094308	0.279058
0.1	0.7	3.950736	1.24E+08	366381.6	130.1071	7.367534	2.521834	0.175448	5.58E-09	1.89E-06	0.005328	0.094081	0.274858
0.1	0.8	4.053943	1.44E+08	321037.8	114.0049	7.385256	2.558506	0.170981	4.82E-09	2.16E-06	0.00608	0.093856	0.270919
0.1	0.9	4.156692	1.64E+08	285770.2	101.4809	7.402987	2.593939	0.166755	4.22E-09	2.43E-06	0.00683	0.093631	0.267218
0.1	1	4.258986	1.85E+08	257556	91.46166	7.420726	2.628171	0.162749	3.75E-09	2.69E-06	0.007579	0.093407	0.263738
0.1	1.1	4.360828	2.06E+08	234471.5	83.26404	7.438475	2.66124	0.158949	3.37E-09	2.96E-06	0.008325	0.093184	0.26046
0.1	1.2	4.462222	2.27E+08	215234.3	76.43265	7.456232	2.693184	0.155337	3.05E-09	3.22E-06	0.009069	0.092962	0.257371
0.1	1.3	4.56317	2.49E+08	198956.6	70.6522	7.473998	2.724039	0.1519	2.78E-09	3.48E-06	0.009811	0.092741	0.254456
0.1	1.4	4.663677	2.72E+08	185004.2	65.6975	7.491772	2.753838	0.148627	2.55E-09	3.75E-06	[illegible]	[illegible]	[illegible]
0.1	1.5	4.763744	2.95E+08	172911.9	61.40338	7.509555	2.782615	0.145505	2.35E-09	4.01E-06	[illegible]	[illegible]	[illegible]
0.1	1.6	4.863376	3.18E+08	162331.1	57.646	7.527347	2.810403	0.142524	2.18E-09	4.27E-06	[illegible]	[illegible]	[illegible]
0.1	1.7	4.962575	3.41E+08	152995.1	54.33064	7.545146	2.837232	0.139675	2.03E-09	4.53E-06	[illegible]	[illegible]	[illegible]
0.1	1.8	5.061344	3.65E+08	144696.3	51.38361	7.562954	2.863132	0.136949	1.90E-09	4.79E-06	0.01349	0.09165	0.242094
0.1	1.9	5.159687	3.89E+08	137270.9	48.74678	7.58077	2.888133	0.134339	1.78E-09	5.05E-06	0.014219	0.091435	0.239998
0.1	2	5.257606	4.14E+08	130588	46.3736	7.598594	2.912263	0.131837	1.67E-09	5.31E-06	0.014947	0.09122	0.23801
0.1	2.1	5.355105	4.39E+08	124541.6	44.22641	7.616426	2.935548	0.129437	1.58E-09	5.57E-06	0.015673	0.091007	0.236122
0.1	2.2	5.452186	4.65E+08	119044.7	42.2744	7.634266	2.958016	0.127132	1.49E-09	5.82E-06	0.016396	0.090794	0.234328
0.1	2.3	5.548852	4.9E+08	114025.8	40.4921	7.652114	2.979691	0.124917	1.41E-09	6.08E-06	0.017118	0.090582	0.232624
0.1	2.4	5.645107	5.16E+08	109425	38.85831	7.669969	3.000599	0.122787	1.34E-09	6.33E-06	0.017838	0.090372	0.231003
0.1	2.5	5.740954	5.43E+08	105192.2	37.3552	7.687832	3.020762	0.120737	1.28E-09	6.59E-06	0.018556	0.090162	0.229461
0.1	2.6	5.836394	5.7E+08	101285	35.96769	7.705703	3.040204	0.118763	1.22E-09	6.84E-06	0.019271	0.089952	0.227994
0.1	2.7	5.931431	5.97E+08	97667.18	34.68295	7.723581	3.058947	0.11686	1.16E-09	7.10E-06	0.019985	0.089744	0.226597
0.1	2.8	6.026068	6.24E+08	94307.69	33.48995	7.741467	3.077013	0.115025	1.11E-09	7.35E-06	0.020697	0.089537	0.225266
0.1	2.9	6.120308	6.52E+08	91179.85	32.37921	7.75936	3.094423	0.113254	1.06E-09	7.60E-06	0.021407	0.08933	0.223999
0.1	3	6.214154	6.8E+08	88260.47	31.3425	7.77726	3.111196	0.111543	1.02E-09	7.85E-06	0.022115	0.089125	0.222791
0.1	3.1	6.307607	7.08E+08	85529.4	30.37266	7.795167	3.127352	0.109891	9.79E-10	8.10E-06	0.022821	0.08892	0.22164

Continues to the right =>

Note that, for readability issues, only 12 of the whole 36 output variables (one variable per column, in addition to those chosen as X and Y) are shown in the figure above.

6.2.1. How to define a variable as constant

The ***savetable*** function always requires the definition of a X and a Y variable. In some cases, however, one just needs to predict what is going to happen in a given environment, where all the input values are constant and no variables are needed. In these cases just one output line is required, which corresponds to a definite set of values of water chemical composition and depth that define the particular environment. To do so, one still has to choose two variables as X and Y, but it is possible to make these variable assume a single value (*i.e.*, they can be treated as constants). For instance, suppose to choose optical path length and NPOC as X and Y variables, respectively, but to have the need to define them as constants (e.g., d = 5 m and NPOC = 3 mg_C L^{-1}). To enter in ***savetable*** d = 5 m and NPOC = 3 mg_C L^{-1}, the following input range should be chosen:

```
% ==================================================================
% *** BEGINNING OF RANGE INPUT ***

 x=5:5:5;  % Range for the first variable, A:B:C means from A to C with steps of B.
 y=3:3:3;    % Range for the second variable.

% *** END OF RANGE INPUT ***
% ==================================================================
```

The output file will contain just one line (and all the columns related to the 38 output variables, of course), corresponding to X=5 and Y=3.

6.3. Correction for the solar zenith angle

When considering the path length travelled by sunlight in water, one should take into account the solar zenith angle (z) and the refraction of sunlight at the air-water interface. Light reflection at the interface also takes place, but it is of lesser importance and can be neglected.[17] The geometry of irradiation can be represented by the scheme below.

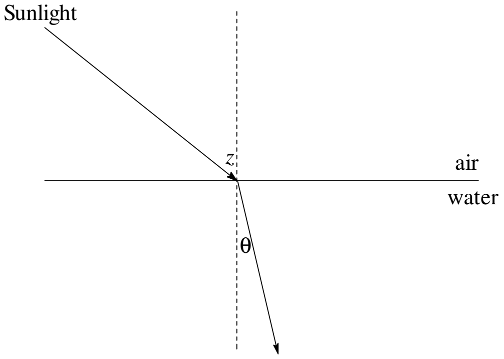

The solar zenith angle z (horizontal system of coordinates) is a function of sun declination δ (geocentric equatorial system of coordinates) and of the hour angle τ. The sun hour angle is defined as the difference between sun's right ascension (geocentric equatorial system of coordinates) and the right ascension of a star on the local meridian. At local noon (when sun is on the local meridian), it is $\tau_{sun} = 0$. Furthermore, every 1 h difference from the local noon gives $\tau \sim 15°$. This means that after 3 h from local noon, the sun has $\tau \sim 45°$. Assume φ as the latitude of the place, and (δ,τ) for the sun as above. The following equation holds for the solar zenith angle:[18]

$$\cos z = \cos\delta \cos\tau \cos\varphi + \sin\delta \sin\varphi \quad (50)$$

Water has refraction index $n \sim 1.34$, which undergoes relatively limited variation with wavelength. It is $\sin z = n \sin\theta$, where θ is the refraction angle as per the scheme above, from which the following relationship can be obtained between the path length d of sunlight and the water column depth h: $h = d\,\cos\theta = d\,\sqrt{1-(\sin\theta)^2}$.[17] Therefore, for depth h and solar zenith angle z, the optical path length d of sunlight inside the water body would be expressed as follows:

$$d = \frac{h}{\sqrt{1-(\sin\theta)^2}} = \frac{h}{\sqrt{1-(n^{-1}\sin z)^2}} \quad (51)$$

This means that water depth could be corrected by a factor $\psi = \left(\sqrt{1-(n^{-1}\sin z)^2}\right)^{-1}$ ($\psi > 1$) that depends on latitude, hour and season. The following plots report the values of ψ that would be observed at different latitudes and months (they are calculated for the 15th day of each month), at

[17] R.G. Zepp, D.M. Cline, Rates of direct photolysis in aquatic environment. Environ. Sci. Technol. 11 (1977) 359-366.
[18] O. Montenbruck, T. Pfleger, Astronomy on the Personal Computer, 2nd Edition. Springer, Berlin, 1994.

the solar noon (τ_{sun} = 0) and at ± 3 h from noon (τ_{sun} ~ ± 45°). The latter would constitute a reasonable daily average in many cases. Calculations of z were carried out with the *Perseus* software.[19]

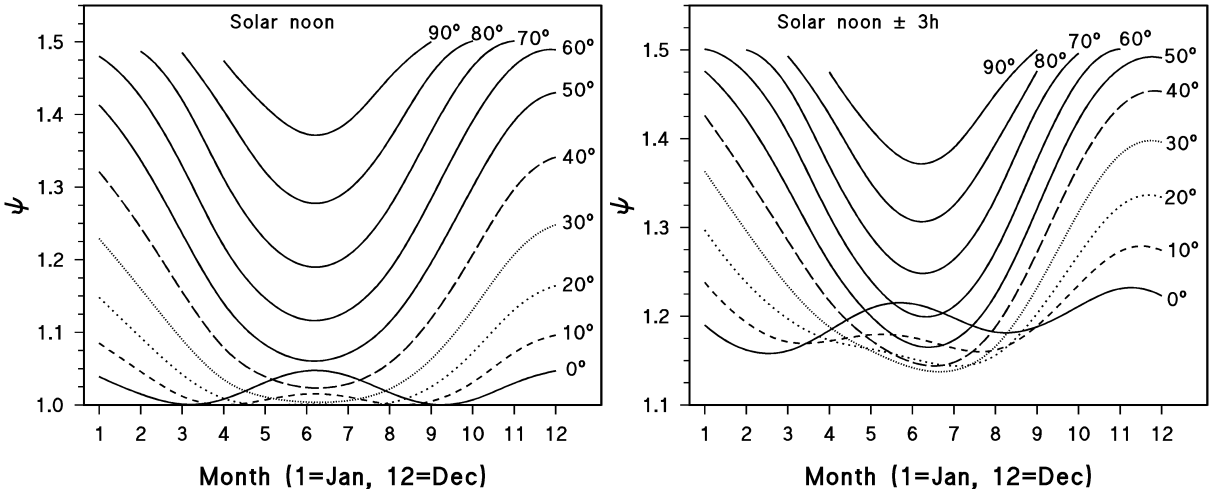

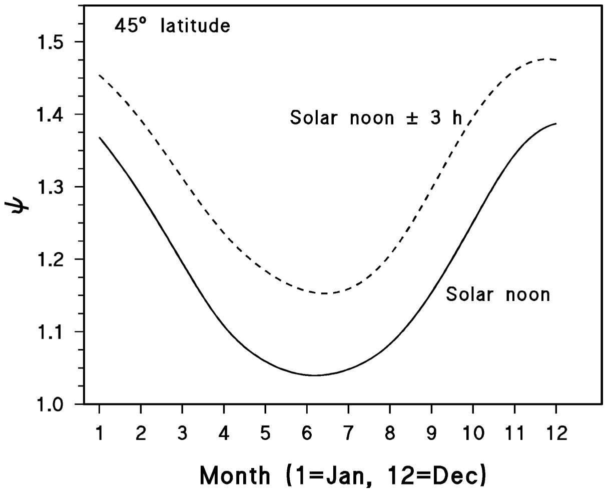

For example, assume a water body with an average depth of 1 m. On May at 50°N latitude one has $\psi \approx 1.2$ (noon ± 3h). Therefore, as input datum in **plotgraph** or **savetable** one should put d = 1.2.

Figure 2. Values of ψ as a function of month and latitude, for the local solar noon and for solar noon ± 3h. In the upper plots the latitude value is specified near each curve. The 45° latitude is highlighted because it is the standard one used in the model. In the case of noon ± 3h the calculations were carried out for 3 pm; in the case of 9 am the symmetry of the curves would be opposite. However, differences are much lower than other uncertainties associated to the model.

Note that correcting for the solar zenith angle could not be enough to take the latitude effects into account. For latitude values that are very different from 45°, the sunlight spectrum might be quite different from that used in the model and reported in Figure 1, in particular in the UV region. Therefore, different and more appropriate values for sunlight $p°(\lambda)$ should be used in the input file (see *http://cprm.acom.ucar.edu/Models/TUV/Interactive_TUV*, for such values as a function of the latitude). Also note that the significance attributed here to a "day", as far as sunlight irradiance is concerned, would be largely lost in polar regions where sunlight irradiation can be either continuous or absent.

[19] F. Riccio, 2009. http://www.perseus.it.

7. Running Plotgraph and Savetable under Octave

When starting Octave, a DOS-like windows appears with some notices and finally a prompt, which in the 3.2.4 version reads as follows:

octave-3.2.4.exe:1>

It is possible to enter commands at the prompt. First of all, if the Apex files are in the "Apex" folder under disk C (path: "C:\Apex"), one should call that folder. The command to be entered (followed by "↵") is:

octave-3.2.4.exe:1>cd c:\Apex

The prompt will now be updated as **octave-3.2.4.exe:2>**, and it is possible to launch Apex calculations. In particular, ***plotgraph*** and ***savetable*** can be run here.

7.1. Plotgraph

The easiest way to plot a graph is to have it on the screen, which can be done by entering the following command (always with "↵" at the end):

octave-3.2.4.exe:2>plotgraph()

Note that the command is case-sensitive, and that "plotgraph" has no capital letters. The 3D plot appears in a window and can be rotated for better view, to obtain, *e.g.*, the following result (**X = *d*, Y = NO_3^-, Z = phototransformation rate constant, k_tot**). This is for instance the plot of the first-order transformation rate constant of carbamazepine as a function of depth and nitrate concentration, with 0.2 µM nitrite, 3.5 mg C L^{-1} DOC, 2 mM bicarbonate, 10 µM carbonate, and 10 nM bromide:

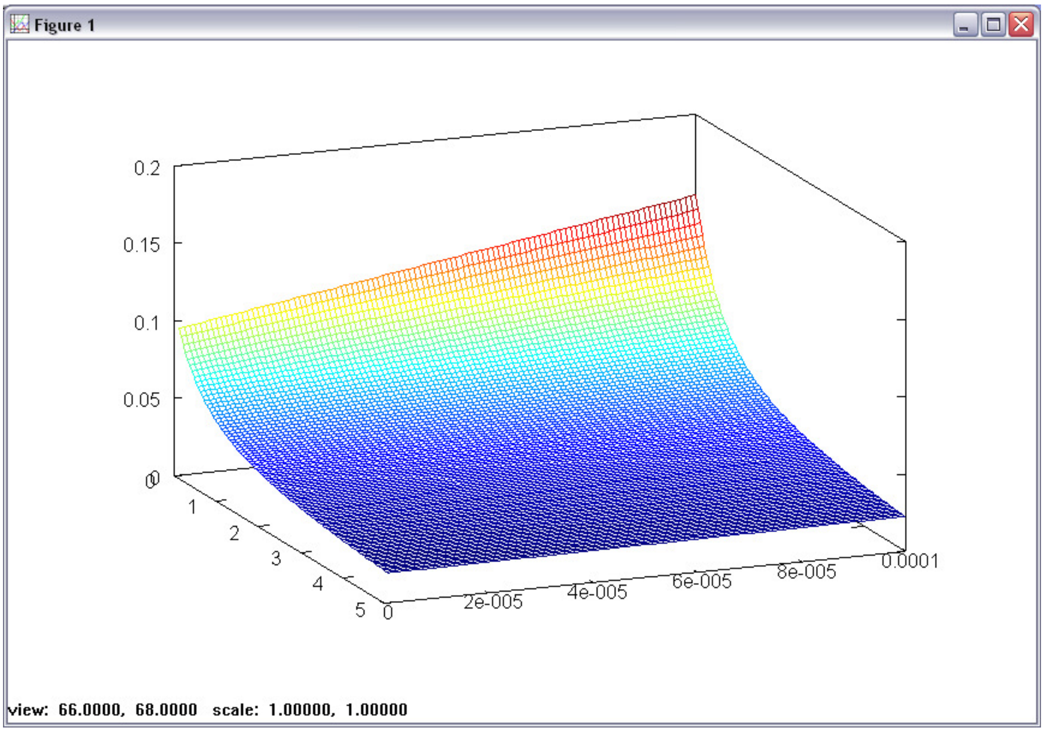

A finer grid (the figure above adopted a 100 × 100 one) will usually give a better aesthetic result, but the computer time needed to perform it should be taken into account. Unfortunately, it is not possible to label the axes. Anyway, the vertical one is always the chosen output variable.

The only way to transform this output in a figure file is to make a print of the screen (Alt+Print or Shift+Print) and to paste it in a graphics file, which is a low-resolution option. As an alternative, a .pdf figure file (*e.g.*, *filename.pdf*) can be generated by entering the following command (followed by "↵"):

octave-3.2.4.exe:3>plotgraph("filename.pdf")

Note the "" around the file name. Obviously, any file name can be given. The advantage is that a usable figure file with better resolution is obtained, but no further rotation is possible. From this point of view, ***plotgraph*** is a good way to have a quick glance at the general trend of Z *vs.* (X,Y), but to obtain a nice high-resolution plot one should often make use of ***savetable***.

7.2. Savetable

The ***savetable*** function returns a table with the X,Y values in the chosen range and all the possible output variables in 36 columns. The first line of the file reports the column titles. The table file is in .csv format (see section 6.2), and the file name is to be specified when entering the command. For instance, to create the table *filename.csv*, one should enter the following (plus "↵"):

octave-3.2.4.exe:3>savetable("filename.csv")

An important issue is that the name of the output .csv file has to be different from the input file name, otherwise the input file will be overwritten.

The output file thus obtained can be the starting point to make a 3D plot with appropriate software. In this way one can obtain 3D graphs with fewer limitations, compared to the ***plotgraph*** function.

IMPORTANT: the files *plotgraph.m* and *savetable.m* that contain the input data should be saved before launching APEX from Octave, otherwise the data thus entered will not be read. The sequence should thus be Open *plotgraph*/*savetable* → insert the data → save the file → launch Octave → call the saved file within Octave. It can be a good idea to have all these files open, as shown in the figure reported in the next page.

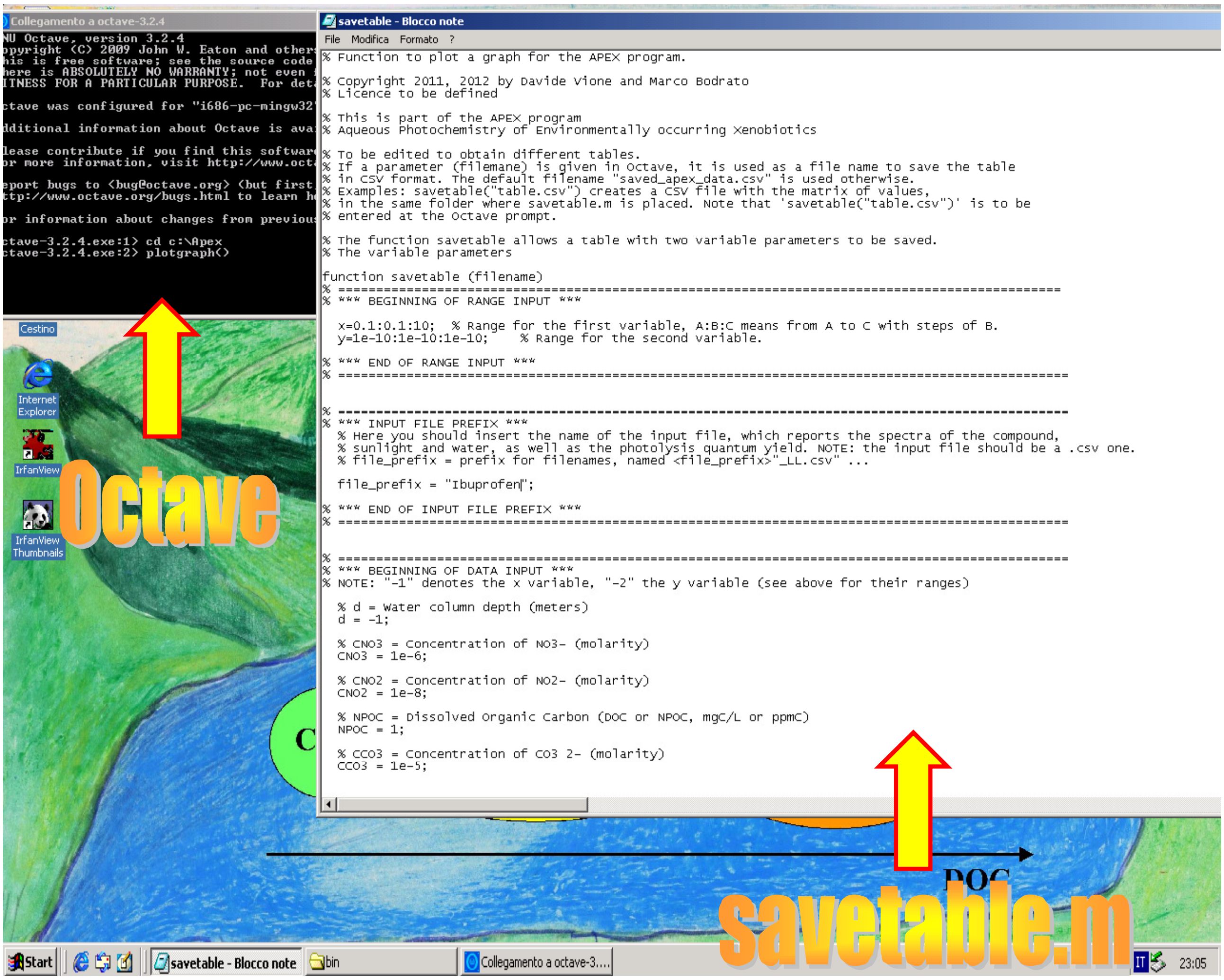
savetable - Blocco note
File Modifica Formato ?
% Function to plot a graph for the APEX program.
% Copyright 2011, 2012 by Davide Vione and Marco Bodrato
% Licence to be defined
% This is part of the APEX program
% Aqueous Photochemistry of Environmentally occurring Xenobiotics
% To be edited to obtain different tables.
% If a parameter (filename) is given in Octave, it is used as a file name to save the table
% in CSV format. The default filename "saved_apex_data.csv" is used otherwise.
% Examples: savetable("table.csv") creates a CSV file with the matrix of values,
% in the same folder where savetable.m is placed. Note that 'savetable("table.csv")' is to be
% entered at the Octave prompt.
% The function savetable allows a table with two variable parameters to be saved.
% The variable parameters
function savetable (filename)
% *** BEGINNING OF RANGE INPUT ***
x=0.1:0.1:10; % Range for the first variable, A:B:C means from A to C with steps of B.
y=1e-10:1e-10:1e-10; % Range for the second variable.
% *** END OF RANGE INPUT ***
% *** INPUT FILE PREFIX ***
% Here you should insert the name of the input file, which reports the spectra of the compound,
% sunlight and water, as well as the photolysis quantum yield. NOTE: the input file should be a .csv one.
% file_prefix = prefix for filenames, named <file_prefix>"_LL.csv" ...
file_prefix = "Ibuprofen";
% *** END OF INPUT FILE PREFIX ***
% *** BEGINNING OF DATA INPUT ***
% NOTE: "-1" denotes the x variable, "-2" the y variable (see above for their ranges)
% d = water column depth (meters)
d = -1;
% CNO3 = Concentration of NO3- (molarity)
CNO3 = 1e-6;
% CNO2 = Concentration of NO2- (molarity)
CNO2 = 1e-8;
% NPOC = Dissolved Organic Carbon (DOC or NPOC, mgC/L or ppmC)
NPOC = 1;
% CCO3 = Concentration of CO3 2- (molarity)
CCO3 = 1e-5;
Collegamento a octave-3.2.4
octave-3.2.4.exe:1> cd c:\Apex
octave-3.2.4.exe:2> plotgraph()
Octave
savetable.m
Start
Collegamento a octave-3....
23:05

7.3. Calculation of model errors

The model calculations are unavoidably subject to an uncertainty, which combines both the errors on the photochemical kinetics parameters of the substrate P (direct photolysis quantum yield and second-order reaction rate constants with $^{\bullet}OH$, $CO_3^{-\bullet}$, $^1O_2$ and $^3CDOM^*$), and the errors on experimentally derived data of surface-water photochemistry (quantum yields of transient formation from CDOM, nitrate and nitrite, scavenging rate constants by DOM). Details of the equations used for error calculation are reported in the Appendix. To make calculation of the errors easier, an Excel file is provided with Apex (C:\Apex\Apex_Errors.xls) that returns absolute and relative errors on all the output parameters. Such parameters should be inserted in the file by copying and pasting a whole line of the output file generated by the ***savetable*** function.

The way the *Apex_Errors.xls* file looks like is shown in the picture below (note that the file is cut here for space limitation issues, but it continues on the right side).

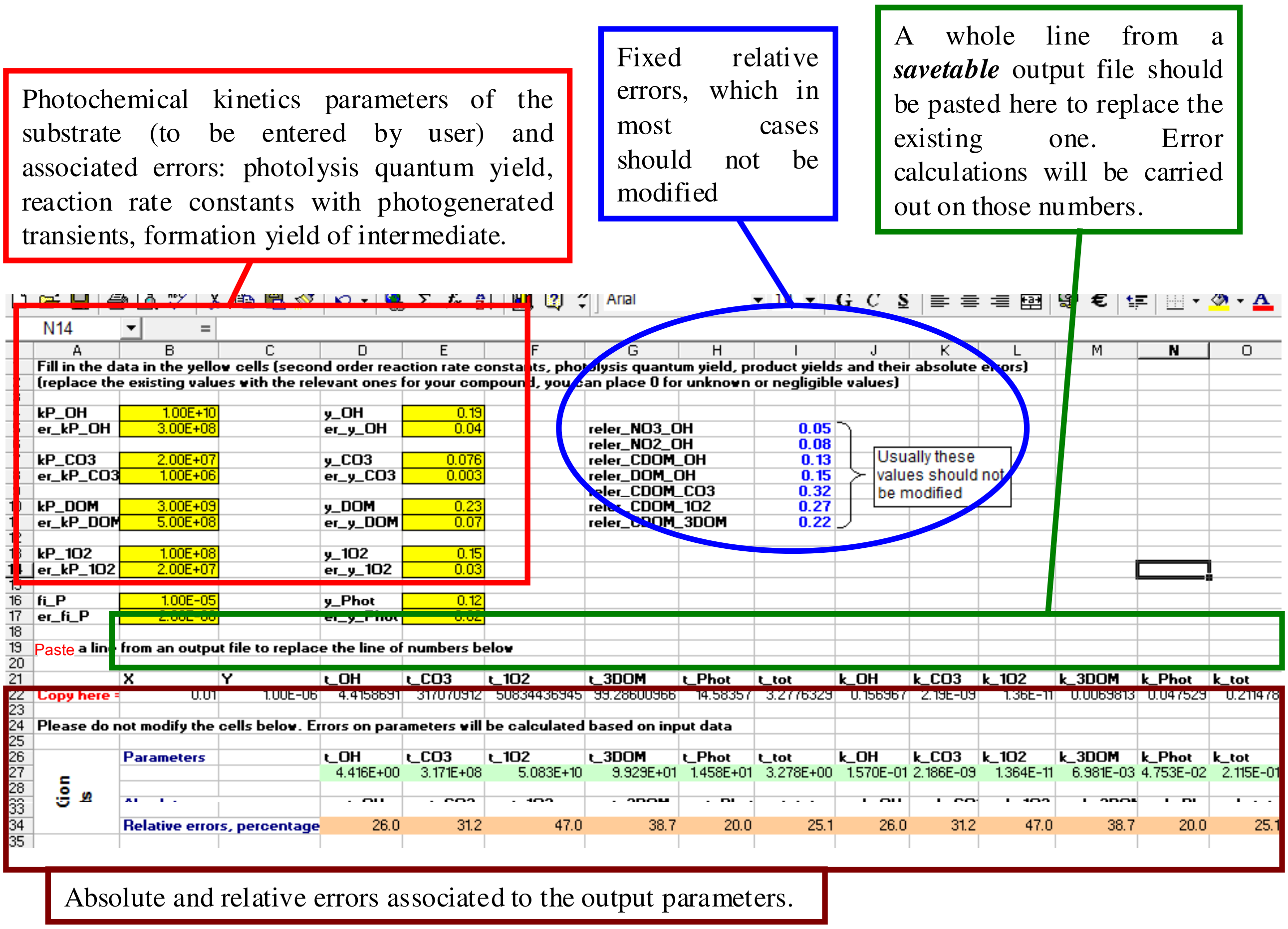

The user should enter data in the yellow cells at the top left corner (photochemical kinetics parameters of the substrate, including -if available- the intermediate formation yields), and paste where indicated a whole line of an output file generated by the ***savetable*** function.

7.4. Seasonal corrections at mid latitude

The standard time unit used in the model output is the SSD, summer sunny day, corresponding to fair-weather 15 July at 45°N latitude. Half-life times are thus expressed in SSD units, and first-order rate constants in SSD^{-1}. The results would thus apply to mid-latitude summertime conditions.

If an (approximated) insight is needed into the behaviour that a compound may have at mid latitude in different seasons, it is possible to take into account the variations of sunlight intensity in the different months of the year. Assume $p°(\lambda)$ as the incident spectral photon flux density of sunlight at mid latitude in a given month of the year (15th of that month).[3] The incident photon flux P_o can be calculated as the integral over wavelength of $p°(\lambda)$ in different spectral ranges: 290-320 nm (UVB), 320-400 nm (UVA), and 300-450 nm where most absorption of sunlight by CDOM takes place. The July results are equivalent to a SSD, and the results for the other months can be normalised to those of July. Therefore, one can obtain a SSD-normalised photon flux $\wp°$ for the relevant spectral range, which represents the numerical value by which the rate constants should be multiplied (and the half-life times divided) to obtain representative photoreactivity values in a given month. The equations used to calculate $\wp°$ are reported below.

$$[P°]_{month} = \int_{\lambda} [p°(\lambda)]_{month} \, d\lambda \qquad (52)$$

$$[\wp°]_{month} = \frac{[P°]_{month}}{[P°]_{July}} \qquad (53)$$

The following Table reports the values of $\wp°$ for the different months of the year at mid latitude, for the three spectral ranges mentioned above. Note that $\wp°$ = 1 at 15 July (SSD).

Month	***SSD-normalised photon flux $\wp°$ (mid latitude)***		
	**UVB (290-320 nm)**	**UVA (320-400 nm)**	**300-450 nm**
January	0.0673	0.197	0.214
February	0.168	0.352	0.370
March	0.389	0.596	0.604
April	0.720	0.862	0.860
May	0.924	0.979	0.966
June	1.05	1.03	1.01
July	1.00	1.00	1.00
August	0.985	0.934	0.928
September	0.596	0.664	0.671
October	0.303	0.421	0.439
November	0.101	0.220	0.238
December	0.040	0.129	0.143

The $\wp°$ values reported in the previous table are referred to different spectral ranges that affect different photosensitisers. In fact, the UVB values can be applied to nitrate photolysis, the UVA ones to nitrite, and those at 300-450 nm to CDOM. In the case of reactions induced by $^1O_2$ and $^3CDOM^*$ that are only produced by CDOM, rate constants should be multiplied by the $\wp°$ values at 300-450 nm (and half-life times divided by the same values). In the case of $^{\bullet}OH$ that is produced by nitrate, nitrite and CDOM, the three different contributions should be corrected by the UVB, UVA and 300-450 nm values, respectively. Note that the relative contributions of nitrate, nitrite and CDOM to $^{\bullet}OH$ generation are given by the output variables NO3_OH, NO2_OH and DOM_OH, respectively, in both ***plotgraph*** and ***savetable***. The radical $CO_3^{-\bullet}$ is mostly produced by oxidation of carbonate and bicarbonate by $^{\bullet}OH$, thus the corrections for $CO_3^{-\bullet}$ are the same as for $^{\bullet}OH$. Finally, in the case of the direct photolysis, the correction would depend on the spectral range where the relevant compound mostly absorbs sunlight (UVB, UVA, or right into the visible). Depending on compound absorption, the most relevant $\wp°$ value should be chosen.

To make these calculations a bit easier, an Excel file is provided in the main Apex folder (C:\Apex\APEX_Season.xls). The file is made up of three sheets named "UVB", "UVA" and "300-450 nm", to be chosen depending on the spectral range where the pollutant mostly absorbs radiation. In each sheet there is the possibility to paste a whole line copied from an output file of ***savetable***, which is referred to a whole set of environmental conditions (see Section 6.2 for the way such an output *.csv* file looks like). For instance, the "UVB" sheet in *APEX_Season.xls* looks like as follows (the others are almost identical):

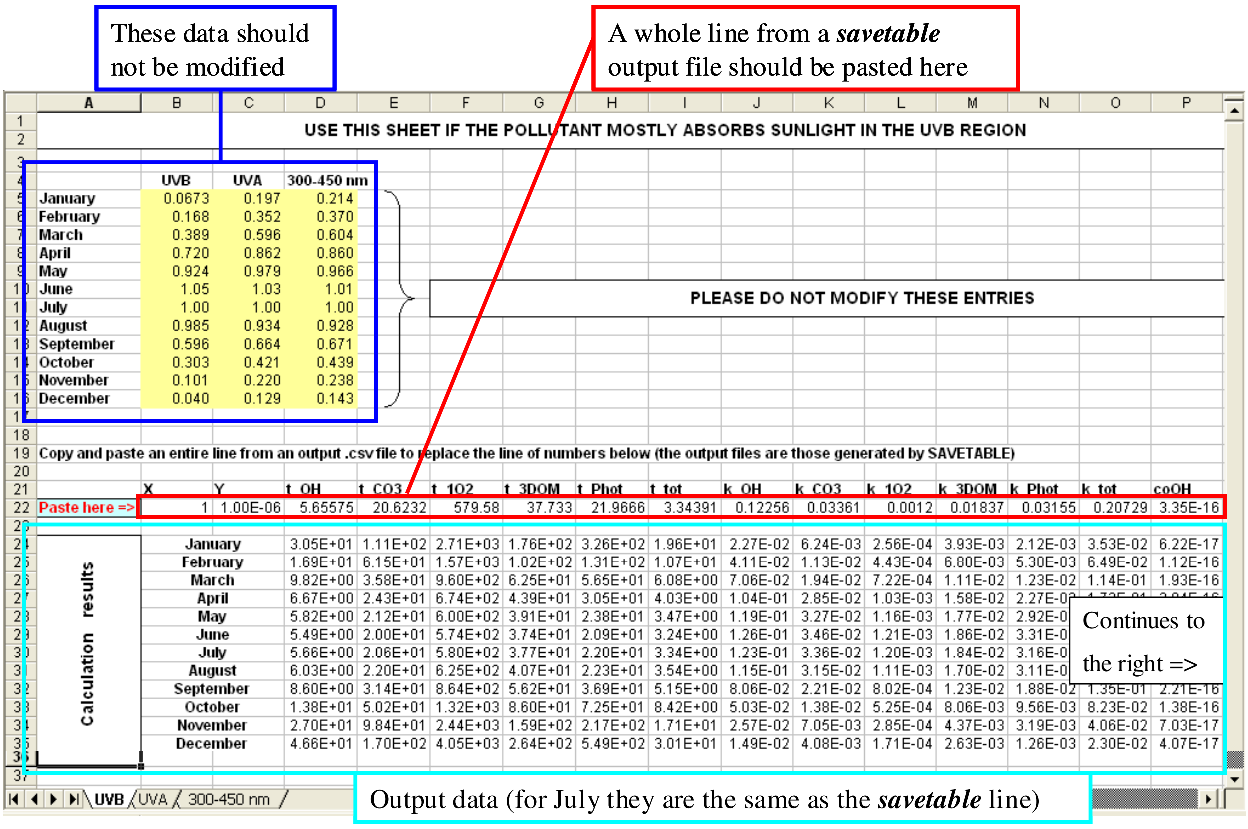

	UVB	UVA	300-450 nm
January	0.0673	0.197	0.214
February	0.168	0.352	0.370
March	0.389	0.596	0.604
April	0.720	0.862	0.860
May	0.924	0.979	0.966
June	1.05	1.03	1.01
July	1.00	1.00	1.00
August	0.985	0.934	0.928
September	0.596	0.664	0.671
October	0.303	0.421	0.439
November	0.101	0.220	0.238
December	0.040	0.129	0.143

	X	Y	t_OH	t_CO3	t_1O2	t_3DOM	t_Phot	t_tot	k_OH	k_CO3	k_1O2	k_3DOM	k_Phot	k_tot	coOH
Paste here =>	1	1.00E-06	5.65575	20.6232	579.58	37.733	21.9666	3.34391	0.12256	0.03361	0.0012	0.01837	0.03155	0.20729	3.35E-16

January	3.05E+01	1.11E+02	2.71E+03	1.76E+02	3.26E+02	1.96E+01	2.27E-02	6.24E-03	2.56E-04	3.93E-03	2.12E-03	3.53E-02	6.22E-17
February	1.69E+01	6.15E+01	1.57E+03	1.02E+02	1.31E+02	1.07E+01	4.11E-02	1.13E-02	4.43E-04	6.80E-03	5.30E-03	6.49E-02	1.12E-16
March	9.82E+00	3.58E+01	9.60E+02	6.25E+01	5.65E+01	6.08E+00	7.06E-02	1.94E-02	7.22E-04	1.11E-02	1.23E-02	1.14E-01	1.93E-16
April	6.67E+00	2.43E+01	6.74E+02	4.39E+01	3.05E+01	4.03E+00	1.04E-01	2.85E-02	1.03E-03	1.58E-02	2.27E-0[illegible]	[illegible]	[illegible]
May	5.82E+00	2.12E+01	6.00E+02	3.91E+01	2.38E+01	3.47E+00	1.19E-01	3.27E-02	1.16E-03	1.77E-02	2.92E-0[illegible]	[illegible]	[illegible]
June	5.49E+00	2.00E+01	5.74E+02	3.74E+01	2.09E+01	3.24E+00	1.26E-01	3.46E-02	1.21E-03	1.86E-02	3.31E-0[illegible]	[illegible]	[illegible]
July	5.66E+00	2.06E+01	5.80E+02	3.77E+01	2.20E+01	3.34E+00	1.23E-01	3.36E-02	1.20E-03	1.84E-02	3.16E-0[illegible]	[illegible]	[illegible]
August	6.03E+00	2.20E+01	6.25E+02	4.07E+01	2.23E+01	3.54E+00	1.15E-01	3.15E-02	1.11E-03	1.70E-02	3.11E-0[illegible]	[illegible]	[illegible]
September	8.60E+00	3.14E+01	8.64E+02	5.62E+01	3.69E+01	5.15E+00	8.06E-02	2.21E-02	8.02E-04	1.23E-02	1.88E-02	1.35E-01	2.21E-16
October	1.38E+01	5.02E+01	1.32E+03	8.60E+01	7.25E+01	8.42E+00	5.03E-02	1.38E-02	5.25E-04	8.06E-03	9.56E-03	8.23E-02	1.38E-16
November	2.70E+01	9.84E+01	2.44E+03	1.59E+02	2.17E+02	1.71E+01	2.57E-02	7.05E-03	2.85E-04	4.37E-03	3.19E-03	4.06E-02	7.03E-17
December	4.66E+01	1.70E+02	4.05E+03	2.64E+02	5.49E+02	3.01E+01	1.49E-02	4.08E-03	1.71E-04	2.63E-03	1.26E-03	2.30E-02	4.07E-17

In this way, one gets the approximate monthly trend of the pollutant transformation kinetics (or, as an alternative, the kinetics of intermediate formation or the steady-state concentrations of photoinduced transients). Also note that in the figure of the previous page, the view of the sheet is cut at the right margin (the actual sheet continues to the right to include all the output variables).

Some important issues should be considered when using *APEX_Season.xls*:

1) The time unit is no longer the SSD, but an average sunny day of the month under consideration.
2) Data processing is approximate, in particular as far as the direct photolysis of the pollutant is concerned.
3) The corrections are approximately valid only under mid-latitude conditions. In different regions one would need different correction factors.

Anyway, the combination of the seasonal correction described here and of the correction for the solar zenith angle (see Section 6.3) can give insight into the effect of seasonality on the photochemistry of surface waters. To additionally correct for the solar zenith angle in a given month one should: *(i)* take the relevant ψ value from Figure 2 of section 6.3, *(ii)* multiply by ψ the water depth to obtain the optical path length d to be inserted in ***savetable***, *(iii)* run ***savetable***, and *(iv)* paste a line of the *.csv* output file into *APEX_Season.xls*, looking at the relevant value for that month.

APPENDIX

Model errors

Input data of substrate reactivity and intermediate formation are (or should be) affected by errors. Model assumptions/equations have an uncertainty, and the model output is affected by error as well.

The following table reports the names of variables that are affected by errors, and the names of variables representing ***absolute errors***. The colour code highlights input data (light yellow), and ***output data*** related to the degradation kinetics of the substrate P (light green), steady-state concentrations of reactive transients (pink), intermediate formation kinetics and yield (grey), role of photochemical processes in P transformation (orange), and role of photochemical processes in intermediate (I) formation (light blue).

Quantity	*Description*	*Absol. error*
kP_OH	Reaction rate constant (2nd order) between P and $^{\bullet}$OH (M^{-1} s^{-1})	er_kP_OH
kP_CO3	Reaction rate constant (2nd order) between P and $CO_3^{-\bullet}$ (M^{-1} s^{-1})	er_kP_CO3
kP_DOM	Reaction rate constant (2nd order) between P and ^{3}CDOM* (M^{-1} s^{-1})	er_kP_DOM
kP_1O2	Reaction rate constant (2nd order) between P and $^1O_2$ (M^{-1} s^{-1})	er_kP_1O2
fi_P	Photolysis quantum yield of P (unitless)	er_fi_P
y_OH	Yield of the intermediate I *via* the $^{\bullet}$OH pathway (unitless)	er_y_OH
y_CO3	Yield of the intermediate I *via* the $CO_3^{-\bullet}$ pathway (unitless)	er_y_CO3
y_1O2	Yield of the intermediate I *via* the $^1O_2$ pathway (unitless)	er_y_1O2
y_DOM	Yield of the intermediate I *via* the ^{3}CDOM* pathway (unitless)	er_y_DOM
y_Phot	Yield of the intermediate I *via* direct photolysis (unitless)	er_y_Phot
t_OH	Half-life time of P degradation by $^{\bullet}$OH (SSD)	er_t_OH
t_CO3	Half-life time of P degradation by $CO_3^{-\bullet}$ (SSD)	er_t_CO3
t_1O2	Half-life time of P degradation by $^1O_2$ (SSD)	er_t_1O2
t_3DOM	Half-life time of P degradation by ^{3}CDOM* (SSD)	er_t_3DOM
t_Phot	Half-life time of P degradation by photolysis (SSD)	er_t_Phot
t_tot	Overall half-life time of P degradation (SSD)	er_t_tot
k_OH	First-order rate constant of P degradation by $^{\bullet}$OH (SSD^{-1})	er_k_OH
k_CO3	First-order rate constant of P degradation by $CO_3^{-\bullet}$ (SSD^{-1})	er_k_CO3
k_1O2	First-order rate constant of P degradation by $^1O_2$ (SSD^{-1})	er_k_1O2
k_3DOM	First-order rate constant of P degradation by ^{3}CDOM* (SSD^{-1})	er_k_3DOM
k_Phot	First-order rate constant of P degradation by photolysis (SSD^{-1})	er_k_Phot
k_tot	Overall first-order rate constant of P degradation (SSD^{-1})	er_k_tot
coOH	Steady-state [$^{\bullet}$OH] (mol L^{-1}, 22 W m^{-2} UV irradiance)	er_coOH
coCO3	Steady-state [$CO_3^{-\bullet}$] (mol L^{-1}, 22 W m^{-2} UV irradiance)	er_coCO3
co1O2	Steady-state [$^1O_2$] (mol L^{-1}, 22 W m^{-2} UV irradiance)	er_co1O2
co3DOM	Steady-state [^{3}CDOM*] (mol L^{-1}, 22 W m^{-2} UV irradiance)	er_co3DOM
f_OH	First-order rate constant of I formation by $^{\bullet}$OH (SSD^{-1})	er_f_OH
f_CO3	First-order rate constant of I formation by $CO_3^{-\bullet}$ (SSD^{-1})	er_f_CO3
f_1O2	First-order rate constant of I formation by $^1O_2$ (SSD^{-1})	er_f_1O2
f_3DOM	First-order rate constant of I formation by ^{3}CDOM* (SSD^{-1})	er_f_3DOM
f_Phot	First-order rate constant of I formation by photolysis (SSD^{-1})	er_f_Phot
f_tot	Overall first-order rate constant of I formation (SSD^{-1})	er_f_tot
y_tot	Overall formation yield of I from P (unitless)	er_y_tot
role_OH_P	Role of $^{\bullet}$OH in P degradation (fraction of total reaction, unitless)	er_role_OH_P
role_CO3_P	Role of $CO_3^{-\bullet}$ in P degradation (fraction of total reaction, unitless)	er_role_CO3_P
role_1O2_P	Role of $^1O_2$ in P degradation (fraction of total reaction, unitless)	er_role_1O2_P
role_3DOM_P	Role of ^{3}CDOM* in P degradation (fraction, unitless)	er_role_3DOM_P
role_Phot_P	Role of direct photolysis in P degradation (fraction, unitless)	er_role_Phot_P
role_OH_I	Role of $^{\bullet}$OH in I formation (fraction of total reaction, unitless)	er_role_OH_I

role_CO3_I	Role of $CO_3^{-\bullet}$ in I formation (fraction of total reaction, unitless)	er_role_CO3_I
role_1O2_I	Role of $^1O_2$ in I formation (fraction of total reaction, unitless)	er_role_1O2_I
role_3DOM_I	Role of ^{3}CDOM* in I formation (fraction of total reaction, unitless)	er_role_3DOM_I
role_Phot_I	Role of photolysis in I formation (fraction of total reaction, unitless)	er_role_Phot_I

The equations that compute the generation and scavenging of reactive transients by (C)DOM, nitrate and nitrite are affected by errors. Therefore, there are a number of constants that represent the ***relative errors*** on the quantum yields of $^\bullet$OH production by nitrate, nitrite and CDOM, on $^\bullet$OH scavenging by DOM, and on quantum yields of $CO_3^{-\bullet}$, $^1O_2$ and ^{3}CDOM* generation by CDOM.[1,10,11] They are listed in the table below.

Name of constant	***Value***	***Description***
reler_NO3_OH	0.05	Relative error on quantum yield of $^\bullet$OH production by nitrate
reler_NO2_OH	0.08	Relative error on quantum yield of $^\bullet$OH production by nitrite
reler_CDOM_OH	0.13	Relative error on quantum yield of $^\bullet$OH production by CDOM
reler_DOM_OH	0.15	Relative error on $^\bullet$OH scavenging by DOM
reler_CDOM_CO3	0.32	Relative error on $CO_3^{-\bullet}$ production by CDOM
reler_CDOM_1O2	0.27	Relative error on quantum yield of $^1O_2$ production by CDOM
reler_CDOM_3DOM	0.22	Relative error on quantum yield of ^{3}CDOM* production by CDOM

Absolute errors on the input data (reaction rate constants and quantum yield of P phototransformation, intermediate formation yields) should be provided by the user. Based on these errors and on those reported in the above table, ***Apex_Errors.xls*** computes the absolute errors of the output variables. Before reporting the equations by which absolute errors are calculated, the Table below describes other variables that are used to compute the errors.

Name of variable	***Description***
ROH_NO3	Formation rate of $^\bullet$OH by nitrate
ROH_NO2	Formation rate of $^\bullet$OH by nitrite
ROH_CDOM	Formation rate of $^\bullet$OH by CDOM
ROH_TOT	Total formation rate of $^\bullet$OH
carbonateyieldCO3_CDOM	Quantum yield of $CO_3^{-\bullet}$ generation by CDOM

1) Steady-state [$^\bullet$OH]

$$er\_coOH = coOH\left\{\frac{\begin{bmatrix} reler\_NO3\_OH \times ROH\_NO3 + reler\_NO2\_OH \times ROH\_NO2 + \\ + reler\_CDOM\_OH \times ROH\_CDOM \end{bmatrix}}{ROH\_TOT} + reler\_DOM\_OH\right\}$$

2) Steady-state [$CO_3^{-\bullet}$]

$$er\_coCO3 = \frac{coCO3 * er\_coOH}{coOH}$$

3) Steady-state [$^1O_2$]

$$er\_co1O2 = co1O2 \times reler\_CDOM\_1O2$$

4) Steady-state [^{3}CDOM*]

$$er\_co3DOM = co3DOM \times reler\_CDOM\_3DOM$$

5) Half-life time of P ($^{\bullet}$OH) and first-order rate constant of P transformation ($^{\bullet}$OH).

$$er\_k\_OH = k\_OH \times \left(\frac{er\_kP\_OH}{kP\_OH} + \frac{er\_coOH}{coOH} \right)$$

$$er\_t\_OH = t\_OH \times \frac{er\_k\_OH}{k\_OH}$$

6) Half-life time of P ($CO_3^{-\bullet}$) and first-order rate constant of P transformation ($CO_3^{-\bullet}$).

$$er\_k\_CO3 = k\_CO3 \times \left(\frac{er\_kP\_CO3}{kP\_CO3} + \frac{er\_coCO3}{coCO3} \right)$$

$$er\_t\_CO3 = t\_CO3 \times \frac{er\_k\_CO3}{k\_CO3}$$

7) Half-life time of P ($^1O_2$) and first-order rate constant of P transformation ($^1O_2$).

$$er\_k\_1O2 = k\_1O2 \times \left(\frac{er\_kP\_1O2}{kP\_1O2} + reler\_CDOM\_1O2 \right)$$

$$er\_t\_1O2 = t\_1O2 \times \frac{er\_k\_1O2}{k\_1O2}$$

8) Half-life time of P (^{3}CDOM*) and first-order rate constant of P transformation (^{3}CDOM*).

$$er\_k\_3DOM = k\_3DOM \times \left(\frac{er\_kP\_DOM}{kP\_DOM} + reler\_CDOM\_3CDOM \right)$$

$$er\_t\_3DOM = t\_3DOM \times \frac{er\_k\_3DOM}{k\_3DOM}$$

9) Half-life time of P (photolysis) and first-order rate constant of P transf. (photolysis).

$$er\_k\_Phot = k\_Phot \times \left(\frac{er\_fi\_P}{fi\_P} \right)$$

$$er\_t\_Phot = t\_Phot \times \frac{er\_k\_Phot}{k\_Phot}$$

10) Half-life time of P (total) and first-order rate constant of P transformation (total).

$$er\_k\_tot = er\_k\_OH + er\_k\_CO3 + er\_k\_1O2 + er\_k\_3DOM + er\_k\_Phot$$

$$er\_t\_tot = t\_tot \times \frac{er\_k\_tot}{k\_tot}$$

11) Intermediate formation rate constant ($^{\bullet}$OH)

$$er\_f\_OH = f\_OH \times \left(\frac{er\_y\_OH}{y\_OH} + \frac{er\_k\_OH}{k\_OH} \right)$$

12) Intermediate formation rate constant ($CO_3^{-\bullet}$)

$$er\_f\_CO3 = f\_CO3 \times \left(\frac{er\_y\_CO3}{y\_CO3} + \frac{er\_k\_CO3}{k\_CO3} \right)$$

13) Intermediate formation rate constant ($^1O_2$)

$$er\_f\_1O2 = f\_1O2 \times \left(\frac{er\_y\_1O2}{y\_1O2} + \frac{er\_k\_1O2}{k\_1O2} \right)$$

14) Intermediate formation rate constant (^{3}CDOM*)

$$er\_f\_3DOM = f\_3DOM \times \left(\frac{er\_y\_3DOM}{y\_3DOM} + \frac{er\_k\_3DOM}{k\_3DOM} \right)$$

15) Intermediate formation rate constant (photolysis)

$$er\_f\_Phot = f\_Phot \times \left(\frac{er\_y\_Phot}{y\_Phot} + \frac{er\_k\_Phot}{k\_Phot} \right)$$

16) Intermediate formation rate constant (total)

$$er\_f\_tot = er\_f\_OH + er\_f\_CO3 + er\_f\_1O2 + er\_f\_3DOM + er\_f\_Phot$$

17) Intermediate formation yield (total)

$$er\_y\_tot = y\_tot \times \left(\frac{er\_f\_tot}{f\_tot} + \frac{er\_k\_tot}{k\_tot} \right)$$

18) Fraction of substrate (P) degradation accounted for by $^{\bullet}$OH

$$er\_role\_OH\_P = role\_OH\_P \times \left(\frac{er\_k\_OH}{k\_OH} \right)$$

19) Fraction of substrate (P) degradation accounted for by $CO_3^{-\bullet}$

$$er\_role\_CO3\_P = role\_CO3\_P \times \left(\frac{er\_k\_CO3}{k\_CO3} \right)$$

20) Fraction of substrate (P) degradation accounted for by $^1O_2$

$$er\_role\_1O2\_P = role\_1O2\_P \times \left(\frac{er\_k\_1O2}{k\_1O2} \right)$$

21) Fraction of substrate (P) degradation accounted for by ^{3}CDOM*

$$er\_role\_3DOM\_P = role\_3DOM\_P \times \left(\frac{er\_k\_3DOM}{k\_3DOM} \right)$$

22) Fraction of substrate (P) degradation accounted for by photolysis

$$er\_role\_Phot\_P = role\_Phot\_P \times \left(\frac{er\_k\_Phot}{k\_Phot} \right)$$

23) Fraction of intermediate (I) formation accounted for by $^{\bullet}$OH

$$er\_role\_OH\_I = role\_OH\_I \times \left(\frac{er\_f\_OH}{f\_OH} \right)$$

24) Fraction of intermediate (I) formation accounted for by $CO_3^{-\bullet}$

$$er\_role\_CO3\_I = role\_CO3\_I \times \left(\frac{er\_f\_CO3}{f\_CO3} \right)$$

25) Fraction of intermediate formation accounted for by $^1O_2$

$$er\_role\_1O2\_I = role\_1O2\_I \times \left(\frac{er\_f\_1O2}{f\_1O2} \right)$$

26) Fraction of intermediate formation accounted for by ^{3}CDOM*

$$er\_role\_3DOM\_I = role\_3DOM\_I \times \left(\frac{er\_f\_3DOM}{f\_3DOM} \right)$$

27) Fraction of intermediate formation accounted for by photolysis

$$er\_role\_Phot\_I = role\_Phot\_I \times \left(\frac{er\_f\_Phot}{f\_Phot} \right)$$
